# Supplementary material for: Place-specific factors associated with adverse maternal and perinatal outcomes in Southern Mozambique: a retrospective cohort study
Source: BMJ Open. 2019 Feb 3;9(2):e024042. doi: 10.1136/bmjopen-2018-024042 (PMC6367983; doi:10.1136/bmjopen-2018-024042)
Supplement: Supplementary file 1 [file bmjopen-2018-024042supp001.pdf]

VARIABLE DEFINITIONS:

Variable	Variable Name
FERTRATE	Fertility rate
FAMSUPP_RT	Family support
WRAAGE_RT	Average age of reproductive age woman
UNEMP_RT	Reproductive age women's unemployment rate
ROQI_IMPACT	Flood proneness
PRVTRANS_RT	Private transportation score
LATRINE_RT	Latrine score
WALK_MRD	Isolation
HHH_AVAIL	Household head’s availability
WATER_RT	Water source score
MARRIED_RT	Reproductive age women's marital status score
PUBDRV	Access to secondary health facilities
PUB	Access to primary health facilities
WRAEDUC	Reproductive age women’s education
PUBDRVDRV	Access to tertiary health facilities
HHHEDUC	Household head’s education
FINAUTPRE_RT	Financial autonomy in pregnancy
COMSUPP_RT	Community group support

****

Choose 1 of 18 Summary

Highest Adjusted R-Squared Results

AdjR2	AICc	JB	K(BP)	VIF	SA	Model
0.16	-118.08	0.39	0.15	1.00	0.02	-FERTRATE***
0.10	-115.69	0.18	0.76	1.00	0.04	+ROQI_IMPACT**
0.08	-114.90	0.66	0.05	1.00	0.11	-UNEMP_RT**

Passing Models

AdjR2	AICc	JB	K(BP)	VIF	SA	Model

****

Choose 2 of 18 Summary

Highest Adjusted R-Squared Results

AdjR2	AICc	JB	K(BP)	VIF	SA	Model
0.35	-125.40	0.77	0.03	1.06	0.13	-UNEMP_RT*** -FERTRATE***
0.29	-122.56	0.03	0.26	2.46	0.37	+WRAAGE_RT** -FERTRATE***
0.27	-121.23	0.91	0.35	1.05	0.01	-FAMSUPP_RT** -FERTRATE***

Passing Models

AdjR2	AICc	JB	K(BP)	VIF	SA	Model

****

Choose 3 of 18 Summary

Highest Adjusted R-Squared Results

AdjR2	AICc	JB	K(BP)	VIF	SA	Model
0.46	-130.66	0.26	0.64	3.68	0.71	-LATRINE_RT*** +WRAAGE_RT*** -FERTRATE***
0.44	-129.33	0.56	0.55	1.50	0.08	-PRVTRANS_RT*** -FAMSUPP_RT*** -FERTRATE***
0.44	-129.23	0.01	0.17	1.65	0.19	-LATRINE_RT*** -FAMSUPP_RT*** -FERTRATE***

Passing Models

AdjR2	AICc	JB	K(BP)	VIF	SA	Model

****

Choose 4 of 18 Summary

Highest Adjusted R-Squared Results

AdjR2	AICc	JB	K(BP)	VIF	SA	Model
0.60	-139.23	0.53	0.04	3.69	0.64	-LATRINE_RT*** -FAMSUPP_RT*** +WRAAGE_RT*** -FERTRATE***
0.52	-132.83	0.18	0.84	3.75	0.94	+ROQI_IMPACT** -LATRINE_RT*** +WRAAGE_RT*** -FERTRATE***
0.52	-132.54	0.46	0.82	1.65	0.15	+PUBDRV** -PRVTRANS_RT*** -FAMSUPP_RT*** -FERTRATE***

Passing Models

AdjR2	AICc	JB	K(BP)	VIF	SA	Model

```
0.602028 -139.231246 0.531046 0.039391 3.694394 0.643045  -LATRINE_RT***  -FAMSUPP_RT***
+WRAAGE_RT***  -FERTRATE***
0.522176 -132.831155 0.175090 0.844169 3.754031 0.939831  +ROQI_IMPACT**  -LATRINE_RT***
+WRAAGE_RT***  -FERTRATE***
0.518237 -132.543818 0.458059 0.818336 1.650636 0.148576  +PUBDRV**  -PRVTRANS_RT***  -FAMSUPP_RT***
-FERTRATE***
0.515228 -132.325907 0.781122 0.499719 3.851258 0.772143  -LATRINE_RT**  -UNEMP_RT**  +WRAAGE_RT***
-FERTRATE***
0.513983 -132.236119 0.325629 0.334396 1.612012 0.103168  -WATER_RT**  -PRVTRANS_RT***
-FAMSUPP_RT***  -FERTRATE***
0.510264 -131.969343 0.166281 0.246145 1.982255 0.420674  -LATRINE_RT**  -PRVTRANS_RT**
-FAMSUPP_RT***  -FERTRATE***
******************************************************************************
Choose 5 of 18 Summary
                                    Highest Adjusted R-Squared Results

AdjR2    AICc   JB K(BP)  VIF   SA   Model

 0.65 -141.61 0.54  0.02 3.77 0.67  +ROQI_IMPACT***  -LATRINE_RT***  -FAMSUPP_RT**  +WRAAGE_RT***
-FERTRATE***
 0.64 -140.61 0.63  0.35 3.91 0.98  +WALK_MRD***  +ROQI_IMPACT***  -LATRINE_RT***  +WRAAGE_RT***
-FERTRATE***
 0.63 -139.66 0.49  0.27 3.70 0.30  -LATRINE_RT***  -FAMSUPP_RT***  +WRAAGE_RT***  -FERTRATE***
+HHH_AVAIL*
                                                         Passing Models

AdjR2    AICc        JB       K(BP)    VIF      SA         Model

0.648492 -141.614920 0.536501 0.021780 3.774488 0.668519  +ROQI_IMPACT***  -LATRINE_RT***
-FAMSUPP_RT**  +WRAAGE_RT***  -FERTRATE***
0.638286 -140.613195 0.625085 0.345632 3.908376 0.976892  +WALK_MRD***  +ROQI_IMPACT***
-LATRINE_RT***  +WRAAGE_RT***  -FERTRATE***
0.615682 -138.491555 0.529587 0.076835 3.726831 0.442333  -LATRINE_RT***  +MARRIED_RT**
-FAMSUPP_RT***  +WRAAGE_RT***  -FERTRATE***
0.594995 -136.656610 0.367722 0.363750 3.541431 0.366122  +PUBDRV***  -PRVTRANS_RT***  -FAMSUPP_RT***
 +WRAAGE_RT**  -FERTRATE***
0.582505 -135.593509 0.556140 0.003931 3.357147 0.143597  -WATER_RT***  -PRVTRANS_RT***
-FAMSUPP_RT***  +WRAAGE_RT**  -FERTRATE***
0.568548 -134.442595 0.687727 0.113630 3.178226 0.277763  +WALK_MRD***  +ROQI_IMPACT***
+MARRIED_RT**  +WRAAGE_RT***  -FERTRATE***
0.564733 -134.134426 0.626979 0.464601 3.857918 0.255002  -LATRINE_RT**  -UNEMP_RT**  +WRAAGE_RT***
-FERTRATE***  +HHH_AVAIL**
0.563092 -134.002788 0.353655 0.096116 3.259366 0.415519  +PUBDRVDRV**  -PRVTRANS_RT***
-FAMSUPP_RT***  +WRAAGE_RT**  -FERTRATE***
0.552392 -133.155941 0.795824 0.613240 3.500445 0.197948  +PUBDRV***  +ROQI_IMPACT**  -UNEMP_RT**
+WRAAGE_RT***  -FERTRATE***
0.523659 -130.978388 0.575476 0.009866 3.319401 0.552068  -WATER_RT***  +MARRIED_RT**  -FAMSUPP_RT**
+WRAAGE_RT**  -FERTRATE***
******************************************************************************
Choose 6 of 18 Summary
                                           Highest Adjusted R-Squared Results

AdjR2    AICc   JB K(BP)  VIF   SA   Model

 0.69 -144.18 0.38  0.55 3.91 0.92  +WALK_MRD**  +ROQI_IMPACT***  -LATRINE_RT***  -FAMSUPP_RT**
+WRAAGE_RT***  -FERTRATE***
 0.67 -142.16 0.70  0.10 3.78 0.21  +ROQI_IMPACT***  -LATRINE_RT***  -FAMSUPP_RT**  +WRAAGE_RT***
-FERTRATE***  +HHH_AVAIL**
```

```
 0.67 -141.32 0.49  0.49 4.07 0.94  +PUBDRV  +ROQI_IMPACT**  -LATRINE_RT***  -FAMSUPP_RT***
+WRAAGE_RT***  -FERTRATE***
                                                                Passing Models

AdjR2    AICc        JB       K(BP)    VIF      SA         Model

0.692863 -144.175618 0.379263 0.549371 3.908531 0.923074  +WALK_MRD**  +ROQI_IMPACT***
-LATRINE_RT***  -FAMSUPP_RT**  +WRAAGE_RT***  -FERTRATE***
0.674630 -142.157235 0.695520 0.098209 3.778934 0.213202  +ROQI_IMPACT***  -LATRINE_RT***
-FAMSUPP_RT**  +WRAAGE_RT***  -FERTRATE***  +HHH_AVAIL**
0.635329 -138.166134 0.568820 0.898220 3.550214 0.205750  +PUBDRV***  +ROQI_IMPACT**  -PRVTRANS_RT***
 -FAMSUPP_RT***  +WRAAGE_RT***  -FERTRATE***
0.598989 -134.841346 0.651598 0.008895 3.478494 0.553360  -WATER_RT***  -PRVTRANS_RT**  +MARRIED_RT**
 -FAMSUPP_RT***  +WRAAGE_RT**  -FERTRATE***
0.597255 -134.690338 0.545878 0.001749 3.326038 0.523384  +WALK_MRD**  -PRVTRANS_RT***  +MARRIED_RT**
 -FAMSUPP_RT***  +WRAAGE_RT**  -FERTRATE***
0.592810 -134.306174 0.781534 0.033482 3.269749 0.690837  +ROQI_IMPACT***  -WATER_RT***
-FAMSUPP_RT**  +WRAAGE_RT***  -FERTRATE***  +HHH_AVAIL**
******************************************************************************
Choose 7 of 18 Summary
                                                 Highest Adjusted R-Squared Results

AdjR2    AICc   JB K(BP)  VIF   SA   Model

 0.71 -143.42 0.43  0.45 3.98 0.79  +WALK_MRD**  +ROQI_IMPACT***  -LATRINE_RT***  +MARRIED_RT
-FAMSUPP_RT**  +WRAAGE_RT***  -FERTRATE***
 0.70 -143.13 0.47  0.47 3.93 0.45  +WALK_MRD*  +ROQI_IMPACT***  -LATRINE_RT***  -FAMSUPP_RT**
+WRAAGE_RT***  -FERTRATE***  +HHH_AVAIL
 0.70 -142.49 0.35  0.68 4.31 0.64  +WALK_MRD**  +ROQI_IMPACT**  -LATRINE_RT***  -PRVTRANS_RT
-FAMSUPP_RT***  +WRAAGE_RT***  -FERTRATE***
                                                                       Passing Models

AdjR2    AICc        JB       K(BP)    VIF      SA         Model

0.645867 -136.803684 0.761213 0.081722 3.329961 0.135850  +WALK_MRD***  +ROQI_IMPACT**
-PRVTRANS_RT***  +MARRIED_RT**  -FAMSUPP_RT**  +WRAAGE_RT***  -FERTRATE***
******************************************************************************
Choose 8 of 18 Summary
                                                       Highest Adjusted R-Squared Results

AdjR2    AICc   JB K(BP)  VIF   SA   Model

 0.71 -141.63 0.41  0.29 4.38 0.96  +WALK_MRD**  +ROQI_IMPACT**  -LATRINE_RT**  -PRVTRANS_RT
+MARRIED_RT  -FAMSUPP_RT***  +WRAAGE_RT***  -FERTRATE***
 0.71 -141.02 0.43  0.39 4.04 0.43  +WALK_MRD**  +ROQI_IMPACT***  -LATRINE_RT***  +MARRIED_RT
-FAMSUPP_RT**  +WRAAGE_RT***  -FERTRATE***  +HHH_AVAIL
 0.70 -140.48 0.54  0.63 4.31 0.66  +WALK_MRD**  +ROQI_IMPACT**  -LATRINE_RT***  -PRVTRANS_RT
-FAMSUPP_RT**  +WRAAGE_RT***  -FERTRATE***  +HHH_AVAIL
       Passing Models
AdjR2 AICc JB K(BP) VIF SA   Model
******************************************************************************
Choose 9 of 18 Summary
                                                             Highest Adjusted R-Squared Results

AdjR2    AICc   JB K(BP)  VIF   SA   Model

 0.71 -138.32 0.47  0.33 4.39 0.63  +WALK_MRD**  +ROQI_IMPACT**  -LATRINE_RT***  -PRVTRANS_RT
+MARRIED_RT  -FAMSUPP_RT**  +WRAAGE_RT***  -FERTRATE***  +HHH_AVAIL
 0.71 -138.12 0.43  0.35 4.38 0.68  +WALK_MRD**  +ROQI_IMPACT**  -LATRINE_RT***  -PRVTRANS_RT
```

```
+MARRIED_RT  -FAMSUPP_RT**  +WRAAGE_RT***  -FERTRATE***  +WRAEDUC
 0.71 -138.07 0.43  0.56 4.41 0.95  +WALK_MRD**  +PUBDRVDRV  +ROQI_IMPACT**  -LATRINE_RT**
-PRVTRANS_RT  +MARRIED_RT  -FAMSUPP_RT***  +WRAAGE_RT***  -FERTRATE***
       Passing Models
AdjR2 AICc JB K(BP) VIF SA   Model
******************************************************************************
Choose 10 of 18 Summary
                                                                Highest Adjusted R-Squared
Results
AdjR2    AICc   JB K(BP)  VIF   SA   Model

 0.71 -135.47 0.51  0.49 6.61 0.80  +WALK_MRD***  +ROQI_IMPACT**  -LATRINE_RT**  -PRVTRANS_RT*
+MARRIED_RT*  -FAMSUPP_RT**  +WRAAGE_RT***  -FERTRATE***  -HHHEDUC  +WRAEDUC
 0.71 -134.98 0.43  0.43 4.39 0.80  +WALK_MRD**  +ROQI_IMPACT**  -LATRINE_RT***  -PRVTRANS_RT
+MARRIED_RT  -FAMSUPP_RT**  +WRAAGE_RT***  -FERTRATE***  +HHH_AVAIL  +WRAEDUC
 0.71 -134.76 0.44  0.59 4.67 0.74  +WALK_MRD**  +PUBDRVDRV  +ROQI_IMPACT**  -LATRINE_RT**
-PRVTRANS_RT  +MARRIED_RT  -FAMSUPP_RT***  +FINAUTPRE_RT  +WRAAGE_RT***  -FERTRATE***
       Passing Models
AdjR2 AICc JB K(BP) VIF SA   Model
******************************************************************************
************** Exploratory Regression Global Summary (NEW_OUTC) **************
```

Percentage of Search Criteria Passed

Search Criterion	Cutoff	Trials	# Passed	% Passed
Min Adjusted R-Squared	> 0.50	199139	25678	12.89
Max Coefficient p-value	< 0.05	199139	61	0.03
Max VIF Value	< 4.50	199139	99568	50.00
Min Jarque-Bera p-value	> 0.10	199139	191512	96.17
Min Spatial Autocorrelation p-value	> 0.10	51	43	84.31

Summary of Variable Significance

Variable	% Significant	% Negative	% Positive
FERTRATE	99.82	100.00	0.00
FAMSUPP_RT	44.71	99.88	0.12
WRAAGE_RT	43.32	41.63	58.37
UNEMP_RT	27.99	92.68	7.32
ROQI_IMPACT	23.78	0.00	100.00
PRVTRANS_RT	19.97	94.84	5.16
LATRINE_RT	14.39	67.86	32.14
WALK_MRD	10.09	5.22	94.78
HHH_AVAIL	3.28	2.27	97.73
WATER_RT	2.87	74.80	25.20
MARRIED_RT	2.77	19.79	80.21
PUBDRV	1.74	29.98	70.02
PUB	1.39	81.69	18.31
WRAEDUC	0.86	30.60	69.40
PUBDRVDRV	0.79	60.09	39.91
HHHEDUC	0.24	34.71	65.29
FINAUTPRE_RT	0.10	92.44	7.56
COMSUPP_RT	0.01	45.72	54.28

Summary of Multicollinearity

Variable   VIF Violations Covariates

```
PUB              8.80   34028    PUBDRV (77.21), PUBDRVDRV (31.02), HHHEDUC (12.71), WRAEDUC (9.33),
LATRINE_RT (6.14), FERTRATE (4.87), WALK_MRD (3.30), WATER_RT (3.03), FAMSUPP_RT (0.12), WRAAGE_RT
(0.03), PRVTRANS_RT (0.02)
WALK_MRD         5.89    1610    PUB (3.30), PUBDRVDRV (2.98), PUBDRV (2.34), WATER_RT (0.44),
HHHEDUC (0.21), WRAEDUC (0.21), LATRINE_RT (0.11), FERTRATE (0.05), PRVTRANS_RT (0.02), FAMSUPP_RT
(0.01)
PUBDRV          14.41   63274    PUBDRVDRV (90.54), PUB (77.21), HHHEDUC (22.77), WRAEDUC (16.51),
LATRINE_RT (12.61), FERTRATE (6.57), WATER_RT (6.36), WALK_MRD (2.34), FAMSUPP_RT (0.12), WRAAGE_RT
(0.03), PRVTRANS_RT (0.01)
PUBDRVDRV        8.68   37253    PUBDRV (90.54), PUB (31.02), HHHEDUC (14.17), WRAEDUC (10.57),
LATRINE_RT (7.69), WATER_RT (5.32), FERTRATE (3.03), WALK_MRD (2.98), FAMSUPP_RT (0.02), PRVTRANS_RT
(0.01), WRAAGE_RT (0.01)
ROQI_IMPACT      1.94     0      --------


WATER_RT         5.29    6578    LATRINE_RT (7.08), PUBDRV (6.36), PUBDRVDRV (5.32), PUB (3.03),
HHHEDUC (2.00), WRAEDUC (1.66), FERTRATE (1.13), WALK_MRD (0.44), FAMSUPP_RT (0.00), WRAAGE_RT (0.00)

LATRINE_RT       6.10   13218    PUBDRV (12.61), PUBDRVDRV (7.69), WATER_RT (7.08), HHHEDUC (7.08),
WRAEDUC (6.79), PUB (6.14), FERTRATE (5.99), WALK_MRD (0.11), WRAAGE_RT (0.02)

PRVTRANS_RT      5.04     8      PUB (0.02), WALK_MRD (0.02), PUBDRVDRV (0.01), PUBDRV (0.01),
FAMSUPP_RT (0.00)

MARRIED_RT       1.68     0      --------


UNEMP_RT         3.69     0      --------


FAMSUPP_RT       5.19     54     PUBDRV (0.12), PUB (0.12), HHHEDUC (0.04), WRAEDUC (0.04), PUBDRVDRV
(0.02), WALK_MRD (0.01), WATER_RT (0.00), PRVTRANS_RT (0.00)

COMSUPP_RT       1.86     0      --------


FINAUTPRE_RT     2.26     0      --------


WRAAGE_RT        4.75     38     HHHEDUC (0.10), WRAEDUC (0.10), FERTRATE (0.09), PUB (0.03), PUBDRV
(0.03), LATRINE_RT (0.02), PUBDRVDRV (0.01), WATER_RT (0.00)

FERTRATE         5.42    8320    PUBDRV (6.57), LATRINE_RT (5.99), PUB (4.87), WRAEDUC (3.78),
HHHEDUC (3.69), PUBDRVDRV (3.03), WATER_RT (1.13), WRAAGE_RT (0.09), WALK_MRD (0.05)

HHH_AVAIL        2.62     0      --------


HHHEDUC          8.78   28637    WRAEDUC (58.81), PUBDRV (22.77), PUBDRVDRV (14.17), PUB (12.71),
LATRINE_RT (7.08), FERTRATE (3.69), WATER_RT (2.00), WALK_MRD (0.21), WRAAGE_RT (0.10), FAMSUPP_RT
(0.04)
WRAEDUC          8.89   24552    HHHEDUC (58.81), PUBDRV (16.51), PUBDRVDRV (10.57), PUB (9.33),
LATRINE_RT (6.79), FERTRATE (3.78), WATER_RT (1.66), WALK_MRD (0.21), WRAAGE_RT (0.10), FAMSUPP_RT
(0.04)

------------------------------------------------------------------------------
```

Summary of Residual Normality (JB)

```
      JB    AdjR2        AICc    K(BP)      VIF       SA   Model

0.999981 0.011731  -92.024434 0.002361 4.176647 0.053210  -PUB  +WALK_MRD  -PUBDRVDRV  +ROQI_IMPACT
-WATER_RT  +MARRIED_RT  -FAMSUPP_RT  +COMSUPP_RT  -WRAAGE_RT  +HHHEDUC
0.999979 0.520437 -123.545871 0.883158 4.638126 0.884202  +PUBDRV  -LATRINE_RT*  -UNEMP_RT*
-FINAUTPRE_RT  +WRAAGE_RT***  -FERTRATE***  -HHHEDUC  +WRAEDUC
0.999975 0.099522 -106.528531 0.002699 2.039475 0.005442  -PUBDRVDRV  +ROQI_IMPACT*  -WATER_RT
-FAMSUPP_RT  -WRAAGE_RT  +HHHEDUC
```

Summary of Residual Spatial Autocorrelation (SA)

```
      SA    AdjR2        AICc       JB    K(BP)      VIF   Model

0.976892 0.638286 -140.613195 0.625085 0.345632 3.908376  +WALK_MRD***  +ROQI_IMPACT***
-LATRINE_RT***  +WRAAGE_RT***  -FERTRATE***
0.958004 0.713906 -141.625323 0.409253 0.288293 4.380150  +WALK_MRD**  +ROQI_IMPACT**  -LATRINE_RT**
-PRVTRANS_RT  +MARRIED_RT  -FAMSUPP_RT***  +WRAAGE_RT***  -FERTRATE***
0.954297 0.708834 -138.071313 0.431612 0.559128 4.414522  +WALK_MRD**  +PUBDRVDRV  +ROQI_IMPACT**
-LATRINE_RT**  -PRVTRANS_RT  +MARRIED_RT  -FAMSUPP_RT***  +WRAAGE_RT***  -FERTRATE***
```

```
Table Abbreviations
AdjR2 Adjusted R-Squared
AICc  Akaike's Information Criterion
JB    Jarque-Bera p-value
K(BP) Koenker (BP) Statistic p-value
VIF   Max Variance Inflation Factor
SA    Global Moran's I p-value
Model Variable sign (+/-)
Model Variable significance (* = 0.10, ** = 0.05, *** = 0.01)
```
