# Supplementary material for: Place-specific factors associated with adverse maternal and perinatal outcomes in Southern Mozambique: a retrospective cohort study
Source: BMJ Open. 2019 Feb 3;9(2):e024042. doi: 10.1136/bmjopen-2018-024042 (PMC6367983; doi:10.1136/bmjopen-2018-024042)
Supplement: Supplementary file 2 [file bmjopen-2018-024042supp002.pdf]

Summary of OLS Results - Model Variables

Variable	Coefficient [a]	StdError	t-Statistic	Probability [b]	Robust_SE	Robust_t	Robust_Pr [b]	VIF [c]
Intercept	-0.194483	0.198020	-0.982142	0.334442	0.198192	-0.981289	0.334855	--------
WALK_MRD	0.033353	0.014641	2.278046	0.030557*	0.015198	2.194592	0.036652*	2.065976
ROQI_IMPACT	0.023936	0.007537	3.175867	0.003620*	0.006860	3.489274	0.001620*	1.353110
LATRINE_RT	-0.003094	0.000722	-4.284566	0.000194*	0.000677	-4.572118	0.000088*	1.923468
FAMSUPP_RT	-0.001274	0.000514	-2.480549	0.019399*	0.000602	-2.115857	0.043382*	1.526477
WRAAGE_RT	0.034653	0.008260	4.195438	0.000247*	0.008113	4.271314	0.000201*	2.933353
FERTRATE	-0.222607	0.029423	-7.565753	0.000000*	0.030839	-7.218404	0.000000*	3.908531

OLS Diagnostics

Input Features:	LOCALITY_COMP_PREG	Dependent Variable:	NEW_OUTC
Number of Observations:	35	Akaike's Information Criterion (AICc) [d]:	-144.175618
Multiple R-Squared [d]:	0.747063	Adjusted R-Squared [d]:	0.692863
Joint F-Statistic [e]:	13.783283	Prob(>F), (6,28) degrees of freedom:	0.000000*
Joint Wald Statistic [e]:	139.475044	Prob(>chi-squared), (6) degrees of freedom:	0.000000*
Koenker (BP) Statistic [f]:	4.956762	Prob(>chi-squared), (6) degrees of freedom:	0.549371
Jarque-Bera Statistic [g]:	1.939051	Prob(>chi-squared), (2) degrees of freedom:	0.379263

Notes on Interpretation

* An asterisk next to a number indicates a statistically significant p-value (p < 0.01).

[a] Coefficient: Represents the strength and type of relationship between each explanatory variable and the dependent variable.

[b] Probability and Robust Probability (Robust_Pr): Asterisk (*) indicates a coefficient is statistically significant (p < 0.01); if the Koenker (BP) Statistic [f] is statistically significant, use the Robust Probability column (Robust_Pr) to determine coefficient significance.

[c] Variance Inflation Factor (VIF): Large Variance Inflation Factor (VIF) values (> 7.5) indicate redundancy among explanatory variables.

[d] R-Squared and Akaike's Information Criterion (AICc): Measures of model fit/performance.

[e] Joint F and Wald Statistics: Asterisk (*) indicates overall model significance (p < 0.01); if the Koenker (BP) Statistic [f] is statistically significant, use the Wald Statistic to determine overall model significance.

[f] Koenker (BP) Statistic: When this test is statistically significant (p < 0.01), the relationships modeled are not consistent (either due to non-stationarity or heteroskedasticity). You should rely on the Robust Probabilities (Robust_Pr) to determine coefficient significance and on the Wald Statistic to determine overall model significance.

[g] Jarque-Bera Statistic: When this test is statistically significant (p < 0.01) model predictions are biased (the residuals are not normally distributed).

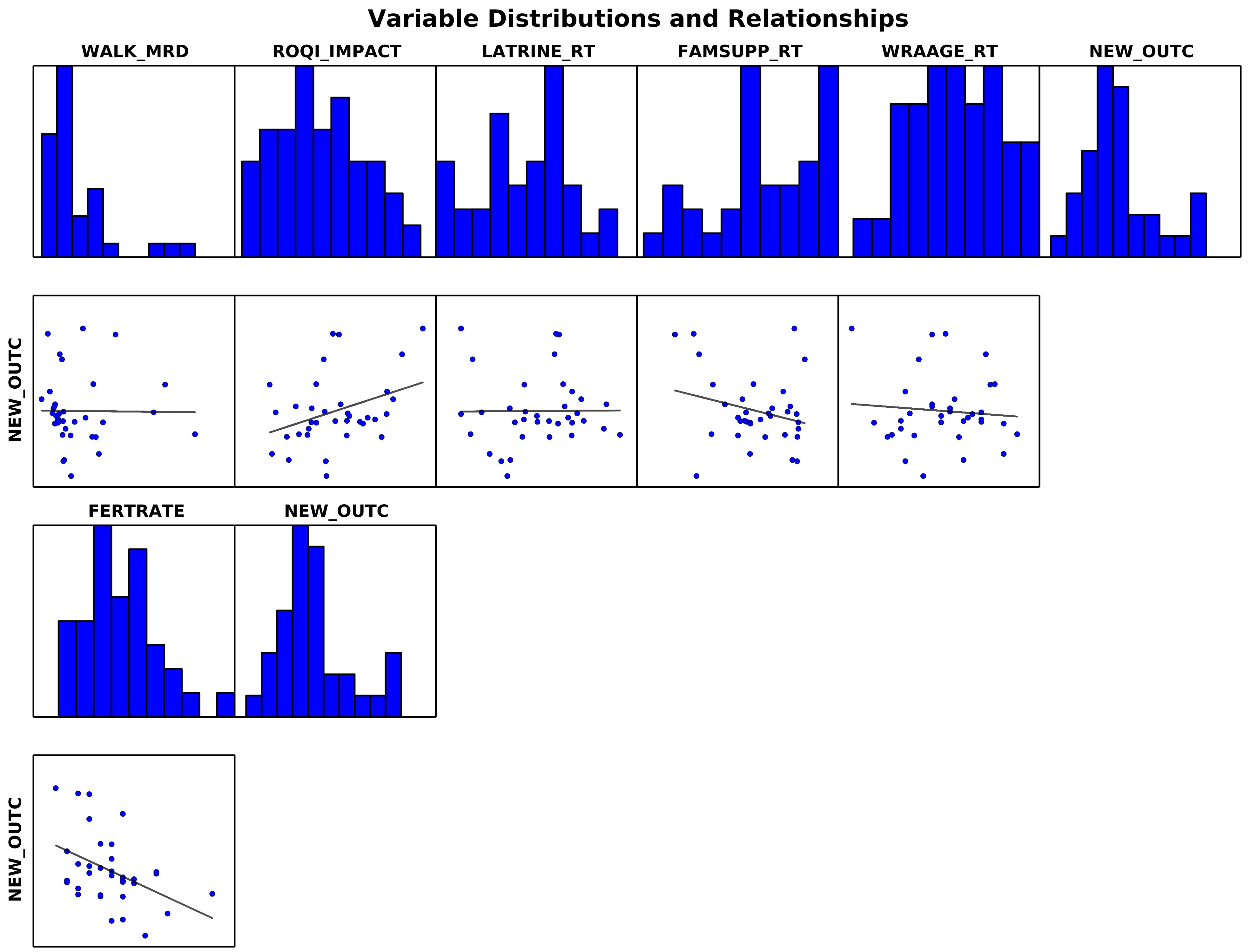
Variable Distributions and Relationships
WALK_MRD
ROQI_IMPACT
LATRINE_RT
FAMSUPP_RT
WRAAGE_RT
NEW_OUTC
NEW_OUTC
FERTRATE
NEW_OUTC
NEW_OUTC

Variable Distributions and Relationships (Cont.)

The above graphs are Histograms and Scatterplots for each explanatory variable and the dependent variable. The histograms show how each variable is distributed. OLS does not require variables to be normally distributed. However, if you are having trouble finding a properly-specified model, you can try transforming strongly skewed variables to see if you get a better result.

Each scatterplot depicts the relationship between an explanatory variable and the dependent variable. Strong relationships appear as diagonals and the direction of the slant indicates if the relationship is positive or negative. Try transforming your variables if you detect any non-linear relationships. For more information see the Regression Analysis Basics documentation.

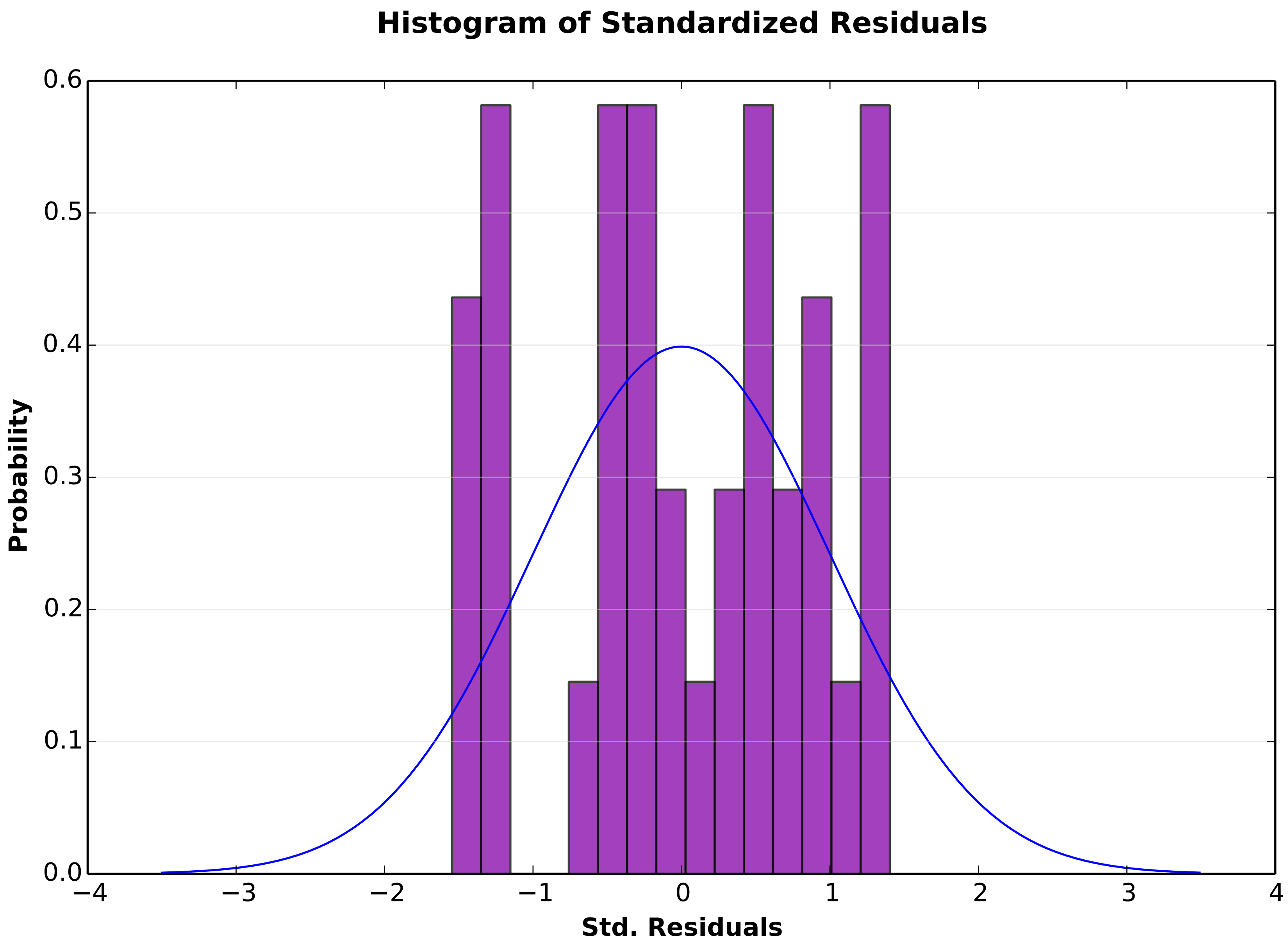

Ideally the histogram of your residuals would match the normal curve, indicated above in blue. If the histogram looks very different from the normal curve, you may have a biased model. If this bias is significant it will also be represented by a statistically significant Jarque-Bera p-value (*).

Residual vs. Predicted Plot

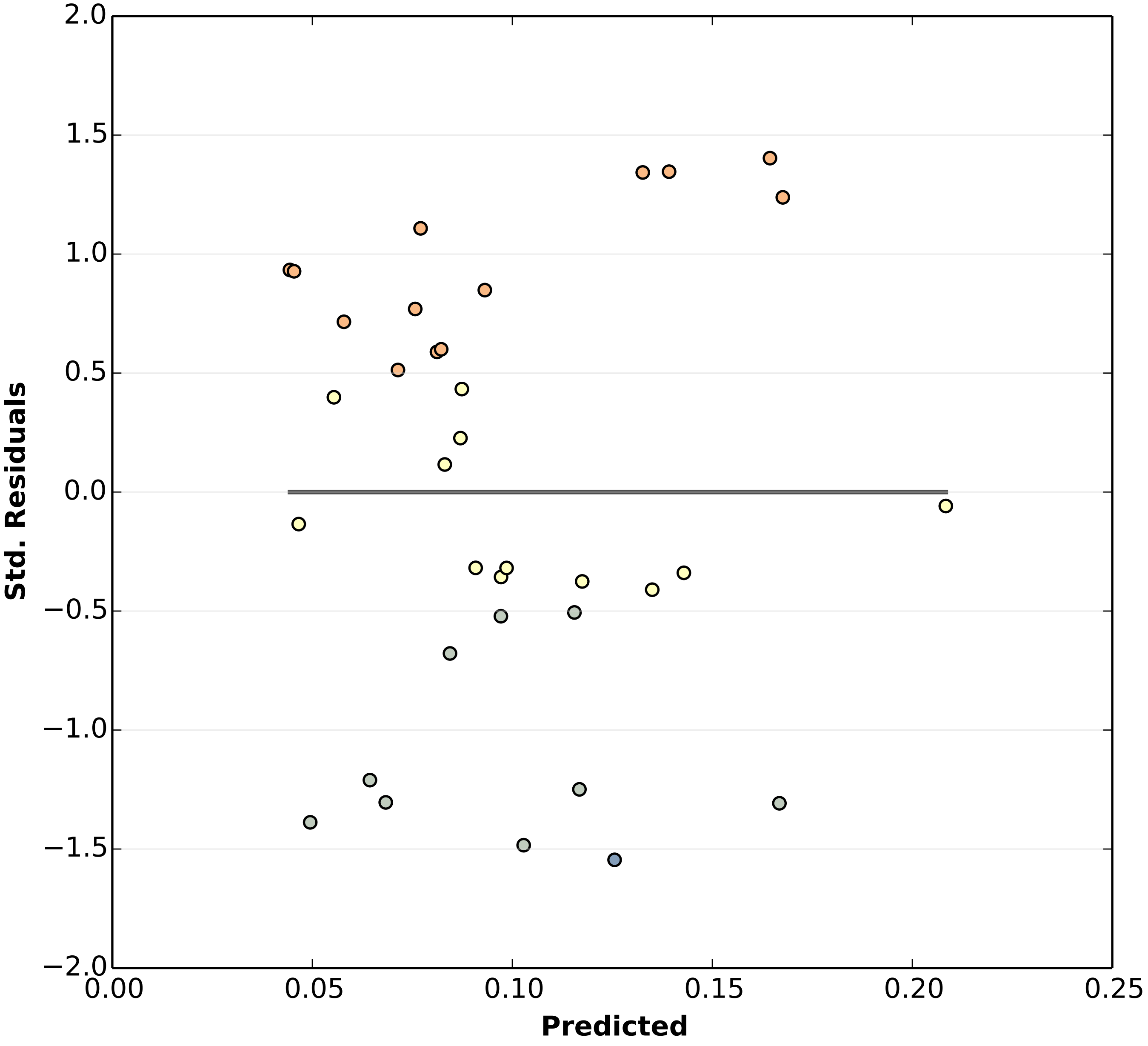

This is a graph of residuals (model over and under predictions) in relation to predicted dependent variable values. For a properly specified model, this scatterplot will have little structure, and look random (see graph on the right). If there is a structure to this plot, the type of structure may be a valuable clue to help you figure out what's going on.

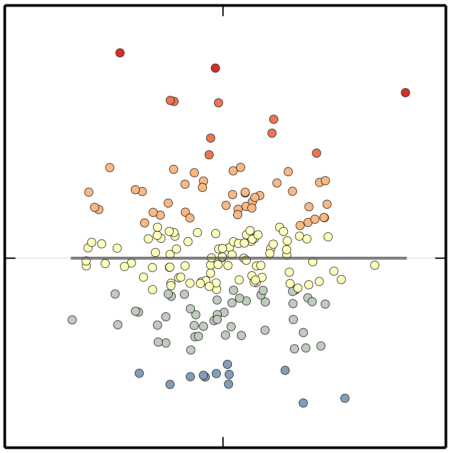

Random Residuals

Ordinary Least Squares Parameters

Parameter Name	Input Value
Input Features	LOCALITY_COMP_PREG
Unique ID Field	UNI_ID
Output Feature Class	None
Dependent Variable	NEW_OUTC
Explanatory Variables	WALK_MRD
	ROQI_IMPACT
	LATRINE_RT
	FAMSUPP_RT
	WRAAGE_RT
	FERTRATE
Selection Set	False
