# Supplementary material for: Place-specific factors associated with adverse maternal and perinatal outcomes in Southern Mozambique: a retrospective cohort study
Source: BMJ Open. 2019 Feb 3;9(2):e024042. doi: 10.1136/bmjopen-2018-024042 (PMC6367983; doi:10.1136/bmjopen-2018-024042)
Supplement: Supplementary file 3 [file bmjopen-2018-024042supp003.pdf]

```
*****************************************************************************
*            Semiparametric Geographically Weighted Regression              *
*                        Release 1.0.90 (GWR 4.0.90)                        *
*                              12 May 2015                                  *
*                (Originally coded by T. Nakaya: 1 Nov 2009)                *
*                                                                           *
*             Tomoki Nakaya(1), Martin Charlton(2), Chris Brunsdon (2)      *
*             Paul Lewis (2), Jing Yao (3), A Stewart Fotheringham (4)      *
*                       (c) GWR4 development team                           *
* (1) Ritsumeikan University, (2) National University of Ireland, Maynooth, *
*         (3) University of Glasgow, (4) Arizona State University           *
*****************************************************************************

Program began at 2018-04-07 9:13:44 AM

*****************************************************************************
Session: MOZ_GWR_2018
Session control file: C:\Users\Prestige\OneDrive\CANADA\UBC\PRE-EMPT\MOM\Papers\Spatial EPI of
Maternal and Neonatal deaths\DATA\GWR_moz_2018.ctl
*****************************************************************************
Data filename: C:\Users\Prestige\OneDrive\CANADA\UBC\PRE-EMPT\MOM\Papers\Spatial EPI of Maternal and
Neonatal deaths\ForGWR.csv
Number of areas/points: 35

Model settings---------------------------------
Model type: Gaussian
Geographic kernel: adaptive Gaussian
Method for optimal bandwidth search: fixed value
Criterion for optimal bandwidth: AICc
Number of varying coefficients: 7
Number of fixed coefficients:   0

Modelling options---------------------------------
Standardisation of independent variables: On
Testing geographical variability of local coefficients: On
Local to Global Variable selection: OFF
Global to Local Variable selection: OFF
Prediction at non-regression points: On

Variable settings---------------------------------
Area key: field1: OBJECTID
Easting (x-coord): field14 : POINT_X
Northing (y-coord): field15: POINT_Y
Cartesian coordinates: Euclidean distance
Dependent variable: field3: NEW_OUTC
Offset variable is not specified
Intercept: varying (Local) intercept
Independent variable with varying (Local) coefficient: field4: WALK_MRD
Independent variable with varying (Local) coefficient: field5: RoQI_IMPACT
Independent variable with varying (Local) coefficient: field6: LATRINE_RT
Independent variable with varying (Local) coefficient: field7: FAMSUPP_RT
Independent variable with varying (Local) coefficient: field8: WRAAGE_RT
Independent variable with varying (Local) coefficient: field9: FERTRATE
Number of prediction at non-regression points   98
*****************************************************************************

*****************************************************************************
  Global regression result
*****************************************************************************
```

< Diagnostic information >

Residual sum of squares:	0.018003
Number of parameters:	7

(Note: this num does not include an error variance term for a Gaussian model)

ML based global sigma estimate:	0.022680
Unbiased global sigma estimate:	0.025357
-2 log-likelihood:	-165.714080
Classic AIC:	-149.714080
AICc:	-144.175618
BIC/MDL:	-137.271295
CV:	0.000937
R square:	0.747063
Adjusted R square:	0.681487

Variable	Estimate	Standard Error	t(Est/SE)
Intercept	0.100460	0.004298	23.374311
WALK_MRD	0.014010	0.006150	2.278046
RoQI_IMPACT	0.015719	0.004950	3.175867
LATRINE_RT	-0.024529	0.005725	-4.284566
FAMSUPP_RT	-0.012849	0.005180	-2.480549
WRAAGE_RT	0.030268	0.007214	4.195438
FERTRATE	-0.063174	0.008350	-7.565753

****

GWR (Geographically weighted regression) bandwidth selection

****

Bandwidth search <fixed value>: 30

****

GWR (Geographically weighted regression) result

****

Bandwidth and geographic ranges

Bandwidth size: 30.000000

Coordinate	Min	Max	Range
X-coord	3616601.398000	3768895.758000	152294.360000
Y-coord	-2920426.694000	-2791357.504000	129069.190000

Diagnostic information

Residual sum of squares:	0.015258
Effective number of parameters (model: trace(S)):	8.181591
Effective number of parameters (variance: trace(S'S)):	7.198744
Degree of freedom (model: n - trace(S)):	26.818409
Degree of freedom (residual: n - 2trace(S) + trace(S'S)):	25.835562
ML based sigma estimate:	0.020880
Unbiased sigma estimate:	0.024302
-2 log-likelihood:	-171.503374
Classic AIC:	-153.140192
AICc:	-145.606817
BIC/MDL:	-138.859623
CV:	0.000880
R square:	0.785624
Adjusted R square:	0.706519

****

<< Geographically varying (Local) coefficients >>

****

Estimates of varying coefficients have been saved in the following file.
Listwise output file: C:\Users\Prestige\OneDrive\CANADA\UBC\PRE-EMPT\MOM\Papers\Spatial EPI of Maternal and Neonatal deaths\DATA\GWR_moz_2018_listwise.csv

Summary statistics for varying (Local) coefficients

Variable	Mean	STD
Intercept	0.101035	0.000664
WALK_MRD	0.012786	0.001721
RoQI_IMPACT	0.015974	0.000361
LATRINE_RT	-0.024667	0.000923
FAMSUPP_RT	-0.014028	0.001883
WRAAGE_RT	0.029602	0.001537
FERTRATE	-0.062852	0.001075

Variable	Min	Max	Range
Intercept	0.099979	0.101892	0.001913
WALK_MRD	0.010322	0.015769	0.005447
RoQI_IMPACT	0.015421	0.016749	0.001328
LATRINE_RT	-0.025996	-0.023045	0.002951
FAMSUPP_RT	-0.016372	-0.011305	0.005068
WRAAGE_RT	0.027676	0.031744	0.004068
FERTRATE	-0.064533	-0.061240	0.003293

Variable	Lwr Quartile	Median	Upr Quartile
Intercept	0.100351	0.101371	0.101613
WALK_MRD	0.011107	0.012520	0.014249
RoQI_IMPACT	0.015689	0.015875	0.016267
LATRINE_RT	-0.025567	-0.024590	-0.024033
FAMSUPP_RT	-0.015926	-0.013778	-0.012218
WRAAGE_RT	0.028076	0.029603	0.031222
FERTRATE	-0.063924	-0.062449	-0.061996

Variable	Interquartile R	Robust STD
Intercept	0.001262	0.000936
WALK_MRD	0.003142	0.002329
RoQI_IMPACT	0.000579	0.000429
LATRINE_RT	0.001534	0.001137
FAMSUPP_RT	0.003708	0.002748
WRAAGE_RT	0.003146	0.002332
FERTRATE	0.001927	0.001429

(Note: Robust STD is given by (interquartile range / 1.349))

****
GWR ANOVA Table
****

Source	SS	DF	MS	F
Global Residuals	0.018	28.000		
GWR Improvement	0.003	2.164	0.001	
GWR Residuals	0.015	25.836	0.001	2.147056

****
Geographical variability tests of local coefficients
****

Variable F DOF for F test DIFF of Criterion

Intercept	2.000828	0.142	26.818	0.176197
WALK_MRD	7.975741	0.195	26.818	-1.224025
RoQI_IMPACT	0.748443	0.185	26.818	0.530483
LATRINE_RT	3.943637	0.125	26.818	-0.156581
FAMSUPP_RT	15.587840	0.164	26.818	-2.551618
WRAAGE_RT	2.703057	0.215	26.818	0.073263
FERTRATE	2.022504	0.157	26.818	0.190761

Note: positive value of diff-Criterion (AICc, AIC, BIC/MDL or CV) suggests no spatial variability in terms of model selection criteria.

F test: in case of no spatial variability, the F statistics follows the F distribution of DOF for F test.

****

Program terminated at 2018-04-07 9:13:45 AM
