# Supplementary material for: Place-specific factors associated with adverse maternal and perinatal outcomes in Southern Mozambique: a retrospective cohort study
Source: BMJ Open. 2019 Feb 3;9(2):e024042. doi: 10.1136/bmjopen-2018-024042 (PMC6367983; doi:10.1136/bmjopen-2018-024042)
Supplement: Author's manuscript [file bmjopen-2018-024042.draft_revisions.pdf]

BMJ Open

BMJ Open is committed to open peer review. As part of this commitment we make the peer review history of every article we publish publicly available.

When an article is published we post the peer reviewers' comments and the authors' responses online. We also post the versions of the paper that were used during peer review. These are the versions that the peer review comments apply to.

The versions of the paper that follow are the versions that were submitted during the peer review process. They are not the versions of record or the final published versions. They should not be cited or distributed as the published version of this manuscript.

BMJ Open is an open access journal and the full, final, typeset and author-corrected version of record of the manuscript is available on our site with no access controls, subscription charges or pay-per-view fees (http://bmjopen.bmj.com).

If you have any questions on BMJ Open's open peer review process please email editorial.bmjopen@bmj.com

BMJ Open

The place-specific factors associated with adverse maternal, and perinatal outcomes in southern Mozambique

Journal:	*BMJ Open*
Manuscript ID	bmjopen-2018-024042
Article Type:	Research
Date Submitted by the Author:	14-May-2018

Complete List of Authors:	Makanga, Prestige; Midlands State University Faculty of Science and Technology, Surveying and Geomatics<br>Sacoor, Charfudin; Centro de Investigacao em Saude de Manhica, Department of Demography<br>Schuurman, Nadine; Simon Fraser Univ, Geography<br>Lee, Tang; University of British Columbia, Obstetrics & Gynaecology<br>Vilanculo, Faustino; Centro de Investigacao em Saude de Manhica<br>Munguambe, Khatia ; Centro de Investigacao em Saude de Manhica<br>Boene, Helena; Centro de Investigacao em Saude de Manhica<br>Ukah, UV; University of British Columbia, Obstetrics & Gynaecology<br>Vidler, Marianne; University of British Columbia, Obstetrics & Gynaecology<br>Magee, Laura; Kings College London, Obstetrics and Gynaecology<br>Sevene, Esperanca; Centro de Investigacao em Saude de Manhica; Universidade Eduardo Mondlane<br>von Dadelszen, Peter ; Kings College London, Obstetrics and Gynaecology<br>Firoz, Tabassum; Yale New Haven Health System

Keywords:	Maternal health, Global health, Spatial epidemiology, Geostatistics,, Health geography

SCHOLARONE™
Manuscripts

Title: The place-specific factors associated with adverse maternal, and perinatal outcomes in southern Mozambique

Corresponding author: Makanga, Prestige Tatenda
Postal address: Midlands State University Faculty of Science and Technology, Surveying and Geomatics
P. Bag 9055
Senga Township
Gweru, Midlands, ZW
E-mail: makangap@staff.msu.ac.zw
Telephone: +263 54 60409 / +263 54 60450/ +263 54 60667

**Co-Author**	**Institution**
Makanga, Prestige Tatenda	Midlands State University Faculty of Science and Technology, Surveying and Geomatics<br>Gweru, Midlands, ZW
Sacoor, Charfudin	Centro de Investigacao em Saude de Manhica, Department of Demography<br>Rua 12, Bairro Cambeve<br>Manhica, MZ 1929<br>+25821810002
Schuurman, Nadine	Simon Fraser Univ, Geography<br>Vancouver, BC, CAN
Lee, Tang	University of British Columbia, Obstetrics & Gynaecology<br>Vancouver, BC, CAN
Vilanculo, Faustino Carlos	Centro de Investigacao em Saude de Manhica, Manhica, Maputo, MZ
Munguambe, Khatia	Centro de Investigacao em Saude de Manhica, Manhica, Maputo, MZ
Boene, Helena	Centro de Investigacao em Saude de Manhica, Manhica, Maputo, MZ
Ukah, Vivian	University of British Columbia, Obstetrics & Gynaecology<br>Vancouver, BC, CAN
Vidler, Marianne	University of British Columbia, Obstetrics & Gynaecology<br>Vancouver, BC, CAN

For peer review only

Magee, Laura A — Kings College London, Obstetrics and Gynaecology
London, London, UK

Sevene, Esperanca — Centro de Investigacao em Saude de Manhica, Manhica, Maputo, MZ
Universidade Eduardo Mondlane, Maputo, MZ

von Dadelszen, Peter — King's College London School of Medical Education
London, London, UK

Firoz, Tabassum — Yale New Haven Health
New Haven, CT, USA

*CLIP working group — Centro de Investigacao em Saude de Manhica
Manhica, Maputo, MZ
University of British Columbia, Vancouver, BC, CAN

Keywords: Maternal health, global health, health geography, geostatistics, spatial epidemiology

Word count: 4458 words

Summary

What is already known about this subject:

- That maternal and perinatal health outcomes are a function of a broad set of social, cultural, economic and environmental determinants, in addition to some the clinical characteristics for these women and their babies
- That action to improve maternal and perinatal health should address both distal and proximal factors. However, much of this action is dominated by clinical interventions that do not address the other determinants.
- That global health strategies and intervention need to move beyond national averages of disease burden that mask much if the health inequities and inequalities. New methods for further dis-aggregating health data are needed to understand the nuances of factors that lead to negative health outcomes

New findings:

- That mixed method geographical approaches help to elucidate the linkages between maternal and perinatal health and the associated determinants by identifying new local determinants of maternal and perinatal health as they are understood in their local contexts
- That dis-aggregate small area spatial analyses help to identify and measure the place specific factors that are related to adverse maternal and perinatal

For peer review only

outcomes

- Exploring the spatial structure of associations between maternal and perinatal health and its associated determinants helps to know what factor matter in specific places, and the extent to which their effect on health outcomes changes across different geographies

How it might impact on clinical practice in the foreseeable future:

- This study could affect clinical practice by helping to tailor interventions that are informed by what is known to elevate risk the most in specific places. The study could potentially help to improve the impact of clinical care by providing a suite of spatial methods and tools that will complement clinical approaches as a means for improving global maternal and child health

For peer review only

The place-specific factors associated with adverse maternal, and perinatal outcomes in southern Mozambique

Authors: *Makanga, P.T., Sacoor, C., Schuurman, N., Lee, T., Vilanculo, F., Munguambe, K., Boene, H., Ukah, U.V., Vidler, M., Magee, L.A., Sevene, E., von Dadelszen, P., Firoz, T and the CLIP Working Group**

Abstract

Objectives: To identify and measure the place specific determinants that are associated with adverse maternal and perinatal outcomes in the southern region of Mozambique.

Setting: Study conducted during the baseline phase of a community level interventions that was led by community health worker

Participants: A household census identified 50,493 households that were home to 80,483 women of reproductive age (age 12 - 49). Of these women, 14,617 had been pregnant in the 12 months prior to the census, of which 9,172 (61.6%) had completed their pregnancies. For the mother, there were 18 deaths (204.6 MMR) of which the verbal autopsy identified that 38% were from direct, and 62% from indirect causes. For the baby, there were 288 (3.0%) miscarriages, 466 (4.9%) stillbirths, and 8,796 (92.1%) livebirths of which there were 117 neonatal deaths.

Primary and secondary outcome measures: A combined fetal, maternal and neonatal outcome was calculated

Results: A total of six variables were statistically significant ($p \leq 0.05$) in explaining the combined outcome. These included; geographic isolation, flood proneness, access to an improved latrine, average age of reproductive age woman, family support and fertility rates. The performance of the OLS model was an adjusted R2 = 0.69. Three of the variables (isolation, latrine score and family support) showed significant geographic variability in their effect on rates of adverse outcome. Accounting for this modest non-stationary effect through Geographically Weighted Regression increased the adjusted R2 to 0.71.

Conclusions: The community exploration was successful in identifying context-specific determinants of maternal health. The results highlight the need for designing targeted interventions that address the place specific social determinants of maternal health in the study area. The geographic process of identifying and measuring these determinants, therefore, has implications for multisectoral collaboration.

Trial registration: (CLIP, NCT01911494)

Strengths

- This study's methods draw from the value to mixing methods and validates the importance of variables through firstly seeking community perspectives, followed by

For peer review only

expert opinion through a Delphi consensus and lastly a rigorous and thorough process of selecting the most important statistical variables.

- The study's strength is also in the small area analyses that are implemented. This helps to granuralize the data and reveal patterns that are normally masked in national averages. Wider use of such approaches will help to elucidate linkages between disease outcomes and associated determinants in future studies.

Limitation

- The main limitation of the study is that the key statistical association that have been uncovered in this research, particularly those from the geographically weighted regression, are specific to this region of Mozambique and may need to be validated before use in other settings. However, the principles behind the choice of methods could be replicated in other settings to reveal local (rather than generalizable) associations.

Introduction

Improving maternal health has been a global health priority for the past four decades. As global attention shifts from surviving pregnancy and childbirth to ensuring that women thrive throughout their life course (1,2), much remains to be done to lessen the harmful consequences of pregnancy and childbirth. Maternal deaths are only a small portion of the global maternal burden of ill-health; it is estimated that for each death; nearly 20 additional women suffer from life-long disabilities as a result of severe pregnancy-related morbidity (3,4). In line with that, Sustainable Development Goal 3 not only aims to improve maternal care but also focuses on lifelong well-being for women and children, particularly in low and middle income countries where the maternal mortality rate is up to 14 times more prevalent than in high income countries (5).

The known risk factors leading to maternal and perinatal death and morbidity exist both at the level of the individual women and at the level of her community. Some of these factors, although experienced at the individual level, are a function (at least in part) of the broader socio-cultural environment, making it hard to separate the two. Individual-level factors include maternal age (6), level of education for women and their partners (7), contraceptive use (8), birth spacing (9), marital status, social standing, self-esteem, and psycho-social stress (10). Environmental and community-level factors that elevate the risk for adverse pregnancy outcomes include physical isolation from health facilities (11), living in war zones (12,13), natural disasters (14), and religion (13,15) among others.

For peer review only

MMR estimates in Mozambique vary from approximately 250 to 408 deaths per 100,000 live births(16–18), and is among the top 20 countries with the highest MMRs globally. There has been approximately 50% reduction in MMR from 541/100,000 live births since 1990, largely due to falling rates of maternal death resulting from direct (as opposed to indirect) obstetric causes (16). In contrast, there has been a relative increase in maternal deaths from indirect causes (such as HIV, malaria) over the same period, as fewer obstetric interventions address these conditions.

Previous studies conducted in Mozambique have documented some of the determinants of maternal health in the country. In southern Mozambique, women who suffered severe maternal morbidities reported that lack of money for transportation and poor road infrastructure and long distances to health facilities caused delays in reaching health facilities, when they sought emergency pregnancy-related care (17,19). Flooding can isolate communities from obstetric care for months at a time (20). A cultural acceptance of the male decision maker's absence from matters concerning the woman's pregnancy may contribute to increasing the vulnerability of pregnant women (21) who may not be empowered to make decisions concerning their pregnancies (19). Prevailing misconceptions around the causes of pregnancy-related illness (22) are likely to influence women's choices to to access care .

Emerging global maternal health strategies acknowledge the broad nature of the associated determinants of health, and call for multi-sectoral approaches to complement health system enablers for improving maternal, fetal and child health along the continuum of care (1,23). These strategies mirror the aim of the Sustainable Development Goals (SDGs) to "draw on contributions from indigenous peoples, civil society, the private sector and other stakeholders, in line with national circumstances, policies and priorities" (2). Further to that, the SDGs call for greater measurement of disaggregate subnational trends in life-course health outcomes and associated determinants (2). This drive to understand the granular population health trends and associated determinants will likely help to better elucidate the place-specific nature of these associations with maternal and perinatal health outcomes.

The aim of this study was to identify and measure the community specific determinants that are associated with maternal and perinatal ill-health in the southern region of Mozambique. In line with the recommendations from the SDGs, the study sought to gain a local understanding of these determinants and how their associations with adverse maternal outcomes varied geographically.

Design and methods

Study setting

The study was conducted as part of the feasibility study for the Community-Level Interventions in Pre-Eclampsia Trial (CLIP, NCT01911494) in Mozambique. CLIP was a community-based cluster randomized controlled trial, aimed at reducing all-cause maternal and perinatal

For peer review only - http://bmjopen.bmj.com/site/about/guidelines.xhtml

mortality and morbidity in the study region. CLIP was led by the University of British Columbia (UBC), in partnership with the Centro de Investigação em Saúde de Manhiça (CISM) in Mozambique. The feasibility study for CLIP was conducted in 12 clusters made up of 36 smaller administrative regions termed localities within two provinces in the southern part of the country. Ethics approval for the study was obtained by the research and ethics boards at UBC, Simon Fraser University and CISM.

Methods

There were four core aspects of this project that are summarized in Figure 1: 1) gathering data on community perspectives of the determinants of maternal health, 2) prioritizing variables through a Delphi consensus, 3) collecting primary empirical data on the variables, and 4) conducting spatial and statistical analyses to explore the association of these variables with adverse maternal outcomes.

Community perspectives on the determinants of maternal health

Ten focus groups discussions (FGDs) were conducted in four of the 12 clusters in the CLIP study area: Messano, 3 de Fevereiro, Ilha Josina + Calanga and Chongoene. These FGDs involved pregnant women, women of reproductive age, matrons (local birth attendants), male partners, community leaders and community-based health workers. Using purposive sampling combined with snowball sampling techniques, participants for the FGDs were recruited with the assistance of community gatekeepers. The FGDs covered topics regarding the socio-cultural, environmental and economic factors thought to be related to adverse maternal events. .

Semi-structured interviews were conducted with the chiefs in all 12 administrative posts in the study region to better understand the historical context (e.g. civil wars, natural disasters, foreign aid, microfinance etc.) of the communities and how these could impact maternal health.

The FGDs and interviews were conducted in a local language (Changana) following a guide of open-ended questions reflecting the topics, were audio-recorded, transcribed and translated verbatim into Portuguese before a final translation into English.

The full details concerning data collection, coding of the data and thematic analysis have been previously published (24).

Prioritizing variables

A Delphi consensus meeting by teleconference was conducted to prioritize the variables for statistical analysis. The Delphi technique helps with “achieving convergence of opinion concerning real-world knowledge solicited from experts within certain topic areas” (25). The panel of 17 experts had a range of relevant backgrounds, including obstetrics (N=2),

For peer review only

epidemiology (N=2), demography (N=2), health geography (N=1), environmental health (N=1), spatial statistics (N=1), health equity (N=1), health systems research (N=1), medical anthropology (N=4), and mobile health (N=2). A questionnaire was sent to ## members of the Delphi group that could not make the call.

Data collection

The context-specific variables identified for consideration of their association with a combined maternal and perinatal adverse outcome were collected through a household census conducted as part of the CLIP feasibility study (26). The census included information on all women who had been pregnant in the 12 months prior to the census, as well as women of reproductive age who had died. Data collected included individual-level variables (e.g. age, education, and pregnancy history), as well as community characteristics (e.g. availability of the household head and community support initiatives). All reports of maternal, fetal, or perinatal deaths were followed-up with verbal autopsy (27) to classify the cause.

The census identified 50,493 households that were home to 80,483 women of reproductive age (age 12 - 49). Of these women, 14,617 had been pregnant in the 12 months prior to the census, of which 9,172 (61.6%) had completed their pregnancies. For the mother, there were 18 deaths (204.6 MMR) of which the verbal autopsy identified that 38% were from direct, and 62% from indirect causes. For the baby, there were 288 (3.0%) miscarriages, 466 (4.9%) stillbirths, and 8,796 (92.1%) livebirths of which there were 117 neonatal deaths. A full description of the health and socio-demographic profile of the women of reproductive age in the study area has been published (26).

In addition, we collected five geographical variables using geographical information systems. There were three travel times to: (1) primary health facilities, (2) secondary health facilities, and (3) tertiary health facilities, using mixed transport modes for public transport and ambulances. Walking times to the nearest main road (4) were calculated to measure the degree to which communities were geographically isolated. Finally, an indicator for flood proneness (5) was designed based on flood and precipitation records from the previous year (28). These variables and other community-level estimates for the variables captured in the census were calculated for each of the administrative units in the study area as described in Table 1. Both the census and geographical data were aggregated into community-level averages for each of the chosen variables.

Statistical analysis

The primary outcome for this study was a combined maternal and perinatal outcome which were maternal, fetal and neonatal deaths. A composite outcome was chosen as powering the study for maternal death alone would have required a prohibitively large sample size. There is

For peer review only - http://bmjopen.bmj.com/site/about/guidelines.xhtml

clinical plausibility in combining the three outcomes as both fetal and early neonatal outcomes are related to the woman's condition during the antenatal period, while her environment and socio-cultural circumstances have an impact on late neonatal outcomes (29,30).

The spatial statistics module within ArcGIS software (31) was used for exploratory regression to further prioritize variables and to create the global ordinary least squares (OLS) regression model. The exploratory regression exercise evaluated different combinations of our explanatory variables for their fit for an OLS model, and how these explained trends in our outcome variable. This method implements the exploration by screening variables in a forward stepwise sequence, exploring how different combinations of variables fit and perform in the regression model. Using criteria that assessed *p* values significance, multi-collinearity measured by the variance inflation factor (VIF), normality of residuals, and clustering of residuals in space (Table 2), we selected the variables that best explained the outcome and met the criteria of a well specified regression model and explored these through a more rigorous OLS modelling exercise.

For peer review only

Table 1: Community level variables potentially associated with the rates of adverse maternal outcomes

Community level variable	Description *(Variables calculated for reproductive age women with completed pregnancies)*
**Census Variables**	
1. Age of reproductive age woman	Average age of reproductive age woman
2. Household head's education	Average number of years that household heads (man or woman) have spent in school *(No schooling = 0; At least Primary = 7; At least secondary = 12; At least a degree = 16; Graduate = 18, post grad = 20)*
3. Household head's availability	Percentage of households where the household head lives in the house
4. Water source score	Percentage of households that have an improved water source
5. Latrine score	Percentage of households that have an improved latrine
6. Private transportation score	Percentage of reproductive age women who live in a house where someone owns a private car
7. Reproductive age women's education	Average number of years that reproductive age women have spent in school *(No Schooling = 0, grade 5 = 5, grade 7 = 7, grade 10 = 10, grade 12 = 12, Bachelors = 16, Graduate = 18, post grad = 20)*
8. Fertility rate	Average number of children born to each woman in the community that had a completed pregnancy
9. Reproductive age women's marital status score	Percentage of reproductive age women in a marital union (monogamous or polygamous) relative to total with completed pregnancies
10. Reproductive age women's unemployment rate	Proportion of reproductive age women that do not work compared with total reproductive age women with a completed pregnancy
11. Family support	Percentage of reproductive age women that would receive financial, transport and emotional help from family or neighbors for a pregnancy related need
12. Community group support	Percentage of reproductive age women that would receive financial, transport and emotional help from a community-based group for a pregnancy related need
13. Financial autonomy in pregnancy	Percentage households where the reproductive age woman is empowered to make financial decisions concerning her pregnancy
**Geospatial variables**	
14. Access to primary health facilities	Average travel time to the nearest primary health facility, using public transport
15. Access to secondary health	Average travel time to the nearest secondary health facility, using a mix of public transport and an

For peer review only

facilities	ambulance
16. Access to tertiary health facilities	Average travel time to the nearest tertiary health facility, using a mix of public transport and an ambulance
17. Isolation	Average walking time to the nearest main road
18. Flood proneness	The difference between the road quality indicator (RoQI) score on a typical day in the dry season and on the worst day in the wet season. RoQI scores range between 0 and 100 and are a function of the quality of roads in a community

For peer review only

Table 2: Criteria for variable selection prior to regression modeling

Criteria	Description	Threshold
Coefficient p-value	The confidence interval required for p values of coefficients	< 0.05
Variance inflation factor	Measures redundancy of multi-collinearity between the explanatory variables	< 7.5
Jarque Bera p-value	Measures whether the model residuals are normally distributed.	> 0.1
Spatial autocorrelation p-value	Check for spatial clustering of model residuals	> 0.1

Global regression model

The performance of the OLS models chosen from the exploratory regression were assessed based on the magnitude of the adjusted R^2 values. In addition, we checked for significance of p values for the model coefficients. Multi-collinearity between different variables in a model was checked using the lower VIF threshold of five. The Koenker statistic ($p < 0.01$) was used to check if the relationships being modelled were consistent (either due to non-stationarity or heteroskadisticity), while the Wald statistic was used to assess overall model significance. The Jarque Bera test ($p < 0.01$) was used to check if model predictions were biased (i.e. if the model residuals were normally distributed). The model that performed best and met these criteria was selected for further analysis to create a locally-specified model.

Local regression model

The Geographically Weighted Regression (GWR) technique was used to develop a second model, which extended the output from OLS, accounting for spatial structure to estimate local rather than global model parameters (32,33). As part of the modelling process, the spatial weights based on the geographic proximity of observation are applied to give more weight to values that were closer together. GWR4 software (34) was used for this part of the project. The geographic variability test was conducted to assess if there was significant non-stationarity in the coefficients after applying GWR. This test compares the geographically varying parameter with those in the fixed global model, where a negative difference (abbreviated "DIFF OF CRITERION" in GWR4), indicates significant variation in parameter estimates across space (34). We also assessed the performance of the GWR model using the newly calculated values of the adjusted R^2.

For peer review only

Results

Community perspectives and choice of variables

The full list of community-level variables that were considered is presented in Table 1. These variables, gathered from the results of focus group discussions, semi-structured interviews, and the Delphi consensus, represent the local perspectives, expert views and *a priori* study knowledge, reported in full separately (19,24), and summarized below.

From the focus groups, unemployment was consistently mentioned by women of reproductive age as an important factor contributing to ill health in pregnancy. Most women mentioned that there were no jobs although some were involved in domestic work and subsistence agriculture. Unemployment was said to have resulted in financial constraints that limited women's ability to access transport and care, especially to buy medications and pay for costs incurred in facilities. Women described that at times they walked long distances to the facilities because they could not afford transport.

Poor relationships with spouses and difficult relationships with in-laws were cited by women as factors that impact their wellbeing in pregnancy. However, neighbours were identified as a vital source of support for pregnant women. In the absence of government health workers in some rural communities, women relied on matrons for advice during pregnancy, assistance during childbirth and accompanying them to health facilities. Matrons mentioned that they were involved in mediating marital problems and reconciling couples. Women identified that informal community groups were important because without them, women in these communities could not organize structured activities like informal money savings scheme that could potentially be used for pregnancy related emergencies requiring money.

Administrative post chiefs described that the localities had faced several natural disasters including floods, droughts and cyclones. Many of the administrative post chiefs acknowledged the negative impact of extreme weather conditions and poor road infrastructure on adverse maternal outcomes.

Several study areas were sandy and, therefore, required large 4x4 vehicles for transport. Other areas were described as muddy or had potholes that limited transport options in the region. An important consideration for accessing roads was seasonality, particularly the rainy season when many regions were prone to floods.

For peer review only

Statistical analysis

Descriptive statistics

The geographic pattern for the rates of the combined adverse maternal and perinatal outcome is shown in Figure 2; the clusters of Ilha Josina + Calanga, Mazivila, and Chissano were the localities with the highest rates of outcomes.

For women with completed pregnancies, community-level scores for census and geographical variables are summarized in Table 3. The average age of women with complete pregnancies was 26. Ninety-one percent of households reported that the head of household lived in the household. There was large variability in the percentage of households with an improved water source, with the lowest locality level score being 11.9% while the highest was 99%. The number of households with an improved toilet facility was very low, with the best locality level score for this variable being 31.6%. An average of 5.6% of all households reported owning a private vehicle. Most women reported being in a marital union (70.9%). The reported rates of unemployment were surprisingly low (mean 11%), given how this was perceived as an important risk factors in the focus group discussions. The proportion of women who indicated that they would receive either financial, transport or emotional help from family or a neighbour in the event of a pregnancy-related need was 85.6%, while only 3.6% would receive the same from community groups. Twenty-two percent of households indicated that the woman was empowered to make financial decisions concerning her pregnancy.

The average travel time to the primary health facilities was 0.6 hours using public transport. For women who were referred to secondary facilities, this was calculated to take an average of 1.2 hours, assuming that they used public transport to primary facilities and an ambulance to secondary facilities. For tertiary facilities, it was calculated to take and average of 2.1 hours using the same combination of transport modes. The most isolated communities required women to walk 2.0 hours to the nearest main road, while the closest were less than 6 minutes (0.1 hours) away. The ease of travel through communities reduced by an average of 6.8% because of flooding and precipitation during the 12 months prior to the household census.

For peer review only

Table 3: Summary statistics

Community level variable	Min	Max	Mean	Std Dev
**Census Variables**				
1. Average age of Reproductive age women (Years)	24.30	28.00	26.36	0.90
2. Household head's education (Years)	3.60	7.30	5.47	0.88
3. Household head's availability (proportion)	0.80	1.00	0.91	0.07
4. Water source score (%)	11.90	99.00	54.68	25.97
5. Latrine score (%)	0.00	31.60	15.48	8.35
6. Private transportation score (%)	0.00	12.30	5.57	3.05
7. Reproductive age women's education (Years)	3.80	7.20	5.29	0.95
8. Fertility rate (No. per woman)	2.40	3.80	2.89	0.29
9. Reproductive age women's marital status score (%)	52.40	88.90	70.85	8.47
10. Reproductive age women's unemployment rate (proportion)	0.00	0.40	0.11	0.10
11. Family support (%)	61.30	100.0 0	85.61	10.46
12. Community support (%)	0.00	17.30	3.59	3.86
13. Financial autonomy in pregnancy (%)	0.00	45.90	22.15	8.43
**Geospatial variables**				
14. Access to primary health facilities (Hrs)	0.18	1.75	0.61	0.36
15. Access to secondary health facilities (Hrs)	0.29	2.80	1.20	0.54
16. Access to tertiary health facilities (Hrs)	0.97	4.32	2.08	0.74
17. Isolation (travel time to nearest main road in hours)	0.10	2.01	0.54	0.42
18. Flood proneness (%)	5.61	8.27	6.75	0.67
**Rate of adverse outcomes per live birth (Maternal + Neonatal + Miscarriages + Stillbirths)**	**0.01**	**0.21**	**0.10**	**0.05**

Global model

Through exploratory regression, we identified six variables that met pre-specified criteria for inclusion in the model. The resulting OLS model is illustrated in Table 4. The adjusted R^2 was 0.69. A full record of the diagnosis for the OLS is provided as supplementary material. The graduated colour classification maps describing the magnitude of the variables across the study area is presented in figure 3.

For peer review only

Table 4: OLS model

Variable	Coefficient	Std Error	t-Statistic	VIF
Intercept	-0.194483	0.19802	-0.98214	--------
Isolation (Hrs)	0.033353*	0.01464	2.27805	2.06598
Flood proneness (%)	0.023936**	0.00754	3.17587	1.35311
Latrine score (%)	-0.003094***	0.00072	-4.28457	1.92347
Family support (%)	-0.001274*	0.00051	-2.48055	1.52648
Age of reproductive age woman (Years)	0.034653***	0.00826	4.19544	2.93335
Fertility rate (No. of children)	-0.222607***	0.02942	-7.56575	3.90853

* $p \le 0.05$; ** $p \le 0.01$; *** $p \le 0.001$
Multiple $R^2 = 0.75$ Adj $R^2 = 0.69$

The OLS model shows that as the degree of isolation increases, there is an effect of increasing rates of adverse outcomes ($p \le 0.05$). This effect is such that every 1 hour increase in walking time to the nearest main road results in a 3.3% increase in the rates of the combined outcome. There is significantly more isolation in the western region of the study area, particularly for the Magude, Ilha Josina, and Calanga clusters, making women in these areas more vulnerable to the effect of isolation.

Communities that were shown to have more fragile and flood prone road infrastructure during the year prior to data collection were also shown to have elevated rates of the adverse outcome. A 1% increase in flood proneness is shown to increase the rate of adverse outcomes by 2.4% ($p \le 0.01$). Regions in the Ilha Josina + Calanga, Mazivila and Chaimite clusters were the most affected by flooding and precipitation.

Higher rates of availability of an improved latrine are associated with lower rates of the adverse outcome ($p \le 0.001$), with a 1% increase in improved latrines estimated to decrease the outcome by approximately 0.3%. Regions in the Ilha Josina + Calanga, Magude and Chaimite clusters had the lowest rates of improved latrines, while 3 de Fevereiro and Chongoene has the highest rates.

Family support emerged as an important characteristic in reducing the rates of adverse outcomes by 0.1% with a 1% increase in rates of women who would get either transport, financial or emotional help from family or neighbors in the event of a pregnancy related need ($p \le 0.05$). Although the levels of family support are relatively high for all localities (mean 85.6%) there is a north-south divide between the communities that have higher and lower rates

For peer review only

of family support with Maluana + Maciana, Ilha Josina + Calanga and 3 de Fevereiro having the highest rates while Mazivila, Chissano and the northern region of Magude have lower rates.

Average age of women with a completed pregnancy was positively associated with rates of the adverse outcome ($p \leq 0.001$), while fertility rates are negatively associated with the rates of adverse outcomes ($p \leq 0.001$). A one-year increase of the average age of the woman had an effect of increasing the rate of adverse outcomes by 3.5%. An increase in the average fertility rate by 0.1 decreases the outcome by 2.2%. A closer look at the age aggregated data for age and fertility rates (Figure 4) reveals a bi-directional trend in the both relationships with the combined outcome. Rates of the combined outcome are relatively higher for the younger ages of 12 years old and decrease until age 20, where they begin to rise until age 49. A similar pattern exists for fertility rates with women who have lowest fertility rates experiencing higher rates of adverse outcomes that decline until fertility rates of approximately 2.2 before rising again for higher fertility rates.

Local model

The GWR model indicated that the effect on the combined outcome was geographically non-stationary for 3 of the 6 variables (isolation, latrine score and family support) as indicated by the negative DIFF OF CRITERION values in Table 5. There was no non-stationarity for the effect of flood proneness, age of the woman and fertility rate. The performance of the local model improved modestly from the global model to an adjusted R^2 value of 0.7, explaining a further 2% of the variability in the outcome. A graduated color classification was used to describe the magnitude of the effect in each of the localities. The transcripts of the GWR results are available as supplementary material.

Table 5: Results of the geographic variability test

VARIABLE	DIFF OF CRITERION
Intercept	0.176197
Isolation	-1.224025
Flood proneness	0.530483
Latrine score	-0.156581
Family support	-2.551618
Age of reproductive age woman	0.073263
Fertility rate	0.190761

For peer review only

The general direction of the effect in the global model is preserved in the local model (Figure 5). Isolation has a general effect of increasing risk for adverse outcomes across the entire study area. However, the effect is more pronounced by a factor of approximately 53% in the western region of Magude compared to the east in Chongoene. A similar pattern is observed for the effect of flood proneness on the combined outcome, with the western regions of the study area being at greater risk, though the magnitude of the differential effect is much smaller (8%). This is also even though the same areas in the west had lower level of proneness to floods (Figure 3).

Proportion of household with an improved latrine was associated with decreasing the adverse outcomes for all regions under study, though this effect is greatest in magnitude for the eastern region of the study area by a factor of about 12%. Family support has a greater effect of reducing the rates of adverse outcomes in the eastern regions by a factor of about 45%. Average woman's age had an effect of increasing the outcome by up to 15% more in the west than the east. The variability in the association of fertility rates to the rates of adverse outcomes was also non-stationary with the highest effect 5% more than the lowest.

Discussion

This study has explored the place-specific factors that are associated with rates of adverse maternal and perinatal outcomes. Information gathered through focus group discussions and semi-structured interviews enabled us to measure the context specific determinants that were thought to be related to adverse maternal and perinatal outcomes. Some of the variables from the FGDs and interviews were indeed significantly associated with the rates of our combined outcome and include family support, geographic isolation and access to an improved latrine facility. Other noteworthy variables include flood proneness, average age of reproductive age women with completed pregnancy and fertility rates. The effect and the magnitude of the effect of these determinants of the outcome varied between communities though the direction of the effect was largely constant.

Fertility rates were the only variable where the direction of the effect is contrary to common expectation. However, fertility rate was the most significant variable both in the global model ($p \leq 0.001$) and as a single variable in the exploratory regression, so the observed pattern is unlikely a result of effect modification. Instead it is possible that there are other pervasive factors are at play. The graph for fertility rates plotted against the rates of adverse outcomes (Figure 4) indicated that adolescent women with completed pregnancies, also have lower fertility rates but tend have high rates of the adverse outcome (26,35). This trend could have possibly skewed the direction of the effect of this variable. This pattern would be possible as the most completed pregnancies (61%) were from women with fertility rates less than 2.2; therefore, community-level averages would naturally carry more weight from these women's records.

For peer review only

This is the first time that the place-specific socio-cultural and environmental factors related to adverse maternal outcomes have been explored in this region of Mozambique. Similar methods have been used in the USA (36). However, most of these studies emphasize health systems-related variables and how they relate to adverse maternal outcomes. This project's approach of going into communities to meet with local stakeholders is aligned with upcoming strategies within the SDGs for improving maternal health (United Nations, 2015). Core to these new global health strategies is an emphasis on multi-sectoral interventions that broadly consult multiple local stakeholders to understand the context specific factors that may be related to population level health trends. The value of geographical techniques to these new strategies is demonstrated in two ways in this project.

First, we used GIS to design new indicators for some of the context specific variables perceived to be related to adverse maternal outcomes. Our measure of isolation for example (distance from highway) was designed based on local knowledge from the FGDs and interviews and implemented in a GIS. Other GIS variables on access to care were generated based on an understanding from the focus group discussions and relevant literature that most women would either walk or use public transport to primary facilities and be driven to higher level facilities (37–39) .

Second, the use of geographically-explicit techniques, such as GWR, enhances the ability to elucidate the spatial structure of the determinants of health and is in line with a drive within global health (expressed through the SDGs) to measure more granular subnational trends in health (2). Evidence from the local regression model developed for this project highlights that different determinants matter to different extents in different places. This understanding is valuable, particularly for designing and targeting population level interventions for improving maternal and child health because it allows for action in specific places to be informed by what is known to elevate risk the most. This is critical for achieving impact and improving health in a resource limited settings like Mozambique (40,41).

A key limitation for this study is that the results can only be applied at the population level and should not be used to predict outcomes at the individual woman level. This phenomenon is termed ecological fallacy (42) and is a key drawback for conducting population-level studies like this one. Furthermore, this project created a combined outcome for maternal, fetal and neonatal outcomes. The implication for this is that the results may not mirror the actual associations with any of the three outcomes in the combined outcome if considered separately. However, the observed patterns address more broadly, an approach to improving maternal health along the continuum of care that includes thinking about fetal, newborn and child survival together (5). Furthermore, the combined outcome better captures the true impact on families, as tragedies of death of mothers, fetuses and infants do not happen in isolation.

For peer review only

Conclusions

The geographic perspective contributes to new strategies for improving global maternal and perinatal health by providing the tools required to understand local contexts and associated determinants. It also helps to elucidate the geographic structure of associations between these determinants and adverse maternal and perinatal health outcomes. This is crucially important for targeting interventions and can help to operationalize some of the key strategies within the new SDG. While the patterns that characterize the findings of this project are specific to the region of southern Mozambique, and may not be transferable to other settings, this research design could certainly be translated to help with understanding the local factors that elevate risk for adverse maternal and perinatal outcomes. It was outside the purview of this research to explore how this evidence could be translated into action on these community specific social determinants, and future work should address this knowledge gap.

Acknowledgements

Funding: This work was part funded by Grand Challenges Canada- Stars in Global Health program (Grant 0197) and was conducted as part of the PRE-EMPT (Pre-eclampsia/Eclampsia, Monitoring, Prevention and Treatment) initiative supported by the Bill & Melinda Gates Foundation.

*CLIP Working Group: Eusébio Macete, Anifa Vala, Felizarda Amose, Rosa Pires, Zefanias Nhamirre, Marta Macamo, Rogério Chiaú, Analisa Matavele, Ariel Nhancolo, Silvestre Cutana, Ernesto Mandlate, Salésio Macuacua, Cassimo Bique, Sibone Mocumbi, Emília Gonçálves, Sónia Maculuve, Ana Ilda Biz, Dulce Mulungo, Orvalho Augusto, Paulo Filimone, Vivalde Nobela, Corsino Tchavana, Cláudio Nkumbula, Jeffrey Bone, Dustin Dunsmuir, Sharla K Drebit, Chirag Kariya, Mai-Lei Woo Kinshella, Jing Li, Mansun Lui, Beth A. Payne, Asif R Khowaja, Diane Sawchuck, Sumedha Sharma, Domena K. Tu, Ugochi V. Ukah,

References

1. United Nations. The Global strategy for women's, children's and adolescents' health (2016–2030) [Internet]. New York; 2015. Available from: http://www.who.int/life-course/partners/global-strategy/globalstrategyreport2016-2030-lowres.pdf?ua=1

2. United Nations. Transforming our world: the 2030 Agenda for Sustainable Development. See Relat Glob Initiat Transnatl Organ Crime"Organized Crime Cross-Cut Threat Sustain Dev January 2015. 2015;

3. Firoz T, Chou D, von Dadelszen P, Agrawal P, Vanderkruik R, Tunçalp O, et al. Measuring maternal health: focus on maternal morbidity. Bull World Health Organ. 2013;91(10):794–6.

4. Alvarez JL, Gil R, Hernández V, Gil A. Factors associated with maternal mortality in Sub-Saharan Africa: an ecological study. BMC Public Health. 2009;9(1):1–8.

5. World Health Organization. Strategies towards ending preventable maternal mortality (EPMM). 2015;

6. Olowonyo T, Oshin S, Obasanjo-Bello I. Registering in a health facility for delivery protects against maternal mortality in a developing country setting. J Obstet Gynaecol. 2005;25(7):638–41.

7. Wall LL. Dead Mothers and Injured Wives: The Social Context of Maternal Morbidity and Mortality among the Hausa of Northern Nigeria. Stud Fam Plann. 1998;29(4):341–59.

8. Ahmed S, Li Q, Liu L, Tsui AO. Maternal deaths averted by contraceptive use: an analysis of 172 countries. The Lancet. 2012;380(9837):111–25.

9. Conde-Agudelo A, Rosas-Bermúdez A, Kafury-Goeta AC. Effects of birth spacing on maternal health: a systematic review. Am J Obstet Gynecol. 2007;196(4):297–308.

10. Ronsmans C, Graham WJ. Maternal mortality: who, when, where, and why. The Lancet. 2006;368(9542):1189–200.

11. Grzybowski S, Stoll K, Kornelsen J. Distance matters: a population based study examining access to maternity services for rural women. BMC Health Serv Res. 2011;11(1):147.

12. Jambai A, MacCormack C. Maternal Health, War, and Religious Tradition: Authoritative Knowledge in Pujehun District, Sierra Leone. Med Anthropol Q. 1996;10(2):270–86.

13. O'Hare BAM, Southall DP. First do no harm: the impact of recent armed conflict on maternal and child health in Sub-Saharan Africa. JRSM. 2007 Dec 1;100(12):564–70.

14. Nour NN. Maternal health considerations during disaster relief. Rev Obstet Gynecol. 2011;4(1):22.

15. Evjen-Olsen B, Hinderaker SG, Lie RT, Bergsjø P, Gasheka P, Kvåle G. Risk factors for maternal death in the highlands of rural northern Tanzania: a case-control study. BMC Public Health. 2008;8(1):1.

16. Bailey PE, Keyes E, Moran AC, Singh K, Chavane L, Chilundo B. The triple threat of pregnancy, HIV infection and malaria: reported causes of maternal mortality in two nationwide health facility assessments in Mozambique, 2007 and 2012. BMC Pregnancy Childbirth. 2015;15(1):1.

For peer review only

17. David E, Machungo F, Zanconato G, Cavaliere E, Fiosse S, Sululu C, et al. Maternal near miss and maternal deaths in Mozambique: a cross-sectional, region-wide study of 635 consecutive cases assisted in health facilities of Maputo province. BMC Pregnancy Childbirth. 2014;14(1):1–8.

18. Ministerio da Saude (MISAU). INdEIaIII. MocËambique InqueÂ rito DemograÂ fico e de SauÂde 2011. Calverton, Maryland, USA:: MISAU, INE and ICFI., 2013. Calverton, Maryland, USA; 2011.

19. Munguambe K, Boene H, Vidler M, Bique C, Sawchuck D, Firoz T, et al. Barriers and facilitators to health care seeking behaviours in pregnancy in rural communities of southern Mozambique. Reprod Health. 2016;13(1):83–97.

20. Jamisse L, Songane F, Libombo A, Bique C, Faúndes A. Reducing maternal mortality in Mozambique: challenges, failures, successes and lessons learned. Int J Gynecol Obstet. 2004;85(2):203–12.

21. Audet CM, Chire YM, Vaz LM, Bechtel R, Carlson-Bremer D, Wester CW, et al. Barriers to male involvement in antenatal care in rural Mozambique. Qual Health Res. 2015;1049732315580302.

22. Boene H, Vidler M, Sacoor C, Nhama A, Nhacolo A, Bique C, et al. Community perceptions of pre-eclampsia and eclampsia in southern Mozambique. Reprod Health. 2016;13(1):27–37.

23. Kuruvilla S, Bustreo F, Kuo N, Mishra C, Taylor K, Fogstad H, et al. The Global strategy for women's, children's and adolescents' health (2016–2030): a roadmap based on evidence and country experience. Bull World Health Organ. 2016;94(5):398–400.

24. Firoz T, Vidler M, Makanga P, Boene H, Sevene E, Magee L, et al. Community Perspectives on the Determinants of Maternal Health in Rural Southern Mozambique: A Qualitative Study. BMC Reprod Health. 2016;In Press.

25. Hsu C-C, Sandford BA. The Delphi technique: making sense of consensus. Pract Assess Res Eval. 2007;12(10):1–8.

26. Sacoor C, Payne B, Augusto O, Vilanculo F, Nhacolo A, Vidler M, et al. Health and socio-demographic profile of women of reproductive age in rural communities of southern Mozambique. PLOS ONE. 2018 Feb 2;13(2):e0184249.

27. World Health Organization. Verbal autopsy standards: the 2012 WHO verbal autopsy instrument. Geneva WHO. 2012;

For peer review only

28. Global Flood Observatory. Dartmouth Flood Observatory - Space-based Measurement, Mapping, and Modeling of Surface Water For Research, Humanitarian, and Water Management Applications [Internet]. 2016 [cited 2016 Jan 17]. Available from: http://floodobservatory.colorado.edu/

29. Oza S, Lawn JE, Hogan DR, Mathers C, Cousens SN. Neonatal cause-of-death estimates for the early and late neonatal periods for 194 countries: 2000_2013. Bull World Health Organ. 2015;93:19–28.

30. Gardosi J, Madurasinghe V, Williams M, Malik A, Francis A. Maternal and fetal risk factors for stillbirth: population based study. BMJ [Internet]. 2013 Jan 24;346. Available from: http://www.bmj.com/content/346/bmj.f108.abstract

31. ESRI. About ArcGIS. Envrionmental Systems Research Institute [Internet]. 2018 [cited 2018 Apr 5]; Available from: http://www.esri.com/arcgis/about-arcgis

32. Brunsdon C, Fotheringham AS, Charlton ME. Geographically weighted regression: a method for exploring spatial nonstationarity. Geogr Anal. 1996;28(4):281–98.

33. Fotheringham AS, Brunsdon C, Charlton M. Geographically weighted regression: the analysis of spatially varying relationships. John Wiley & Sons; 2003.

34. Nakaya T. GWR4 User Manual: Windows application for geographically weighted regression modelling [Internet]. 2014. Available from: https://geodacenter.asu.edu/drupal_files/gwr/GWR4manual.pdf

35. Pizzol D, Di Gennaro F, Boscardin C, Putoto G, Cuppini E, Pita G, et al. Teenage pregnancies in Mozambique: the experience of "Servicios Amigos dos Adolescentes" clinics in Beira. Afr J AIDS Res. 2018 Jan 2;17(1):32–6.

36. Shoff C, Yang T-C, Matthews S. What has geography got to do with it? Using GWR to explore place-specific associations with prenatal care utilization. GeoJournal. 2012 Jun 1;77(3):331–41.

37. Blanford JI, Kumar S, Luo W, MacEachren AM. It'sa long, long walk: accessibility to hospitals, maternity and integrated health centers in Niger. Int J Health Geogr. 2012;11(1):1–15.

38. Makanga PT, Schuurman N, Sacoor C, Boene HE, Vilanculo F, Vidler M, et al. Seasonal variation in geographical access to maternal health services in regions of southern Mozambique. Int J Health Geogr. 2017;16(1):1.

For peer review only

39. Munjanja SP, Magure T, Kandawasvika G. 11 Geographical Access, Transport and Referral Systems. Matern Perinat Health Dev Ctries. 2012;139.

40. Sartorius B, Sartorius K. Identifying and Targeting Mortality Disparities: A Framework for Sub-Saharan Africa Using Adult Mortality Data from South Africa. Plos One [Internet]. 2013 Aug;8(8). Available from: ://WOS:000323221500083

41. Friberg IK, Kinney MV, Lawn JE, Kerber KJ, Odubanjo MO, Bergh A-M, et al. Sub-Saharan Africa's mothers, newborns, and children: how many lives could be saved with targeted health interventions? PLoS Med. 2010;7(6):e1000295.

42. Dummer TJB. Health geography: supporting public health policy and planning. Can Med Assoc J. 2008 Apr 22;178(9):1177–80.

For peer review only

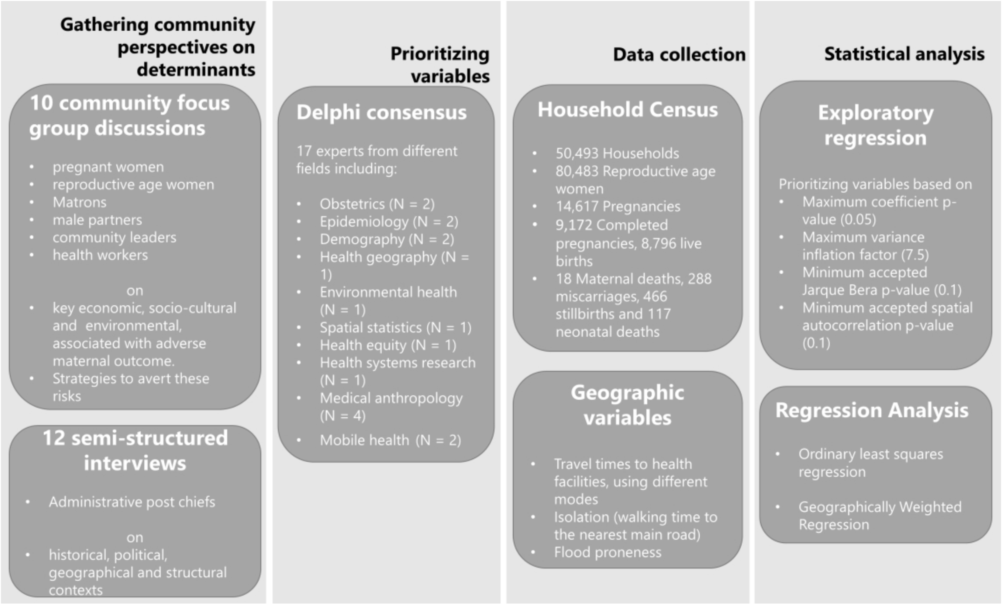

Figure 1: Overview of methods used in the study

389x235mm (300 x 300 DPI)

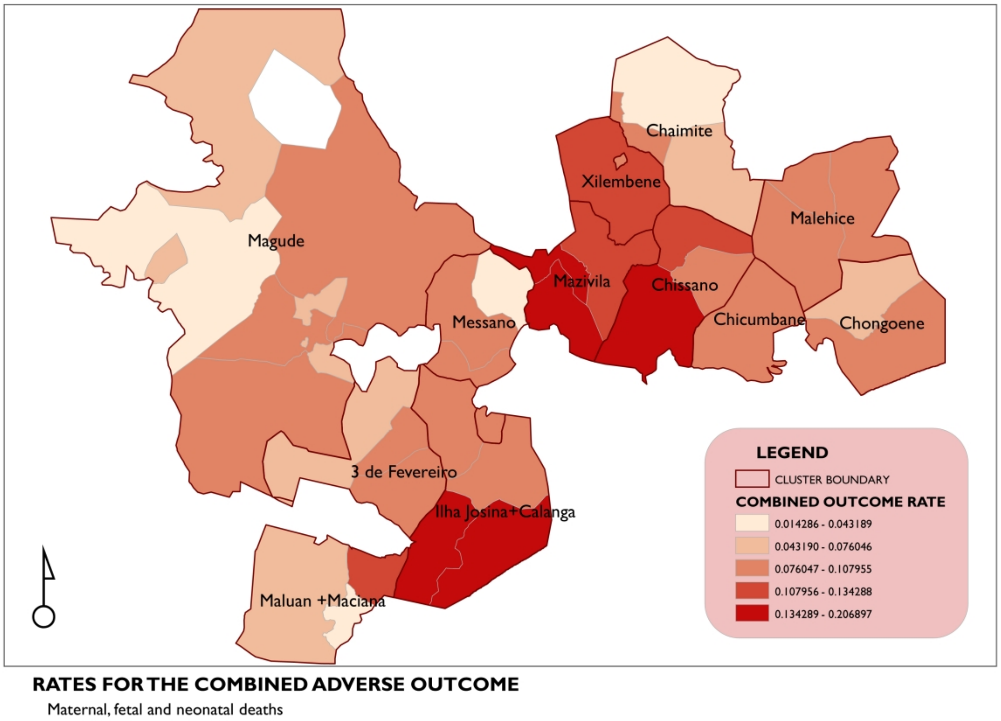

Figure 2: Geographic pattern for the rates of the combined adverse outcomes

312x227mm (300 x 300 DPI)

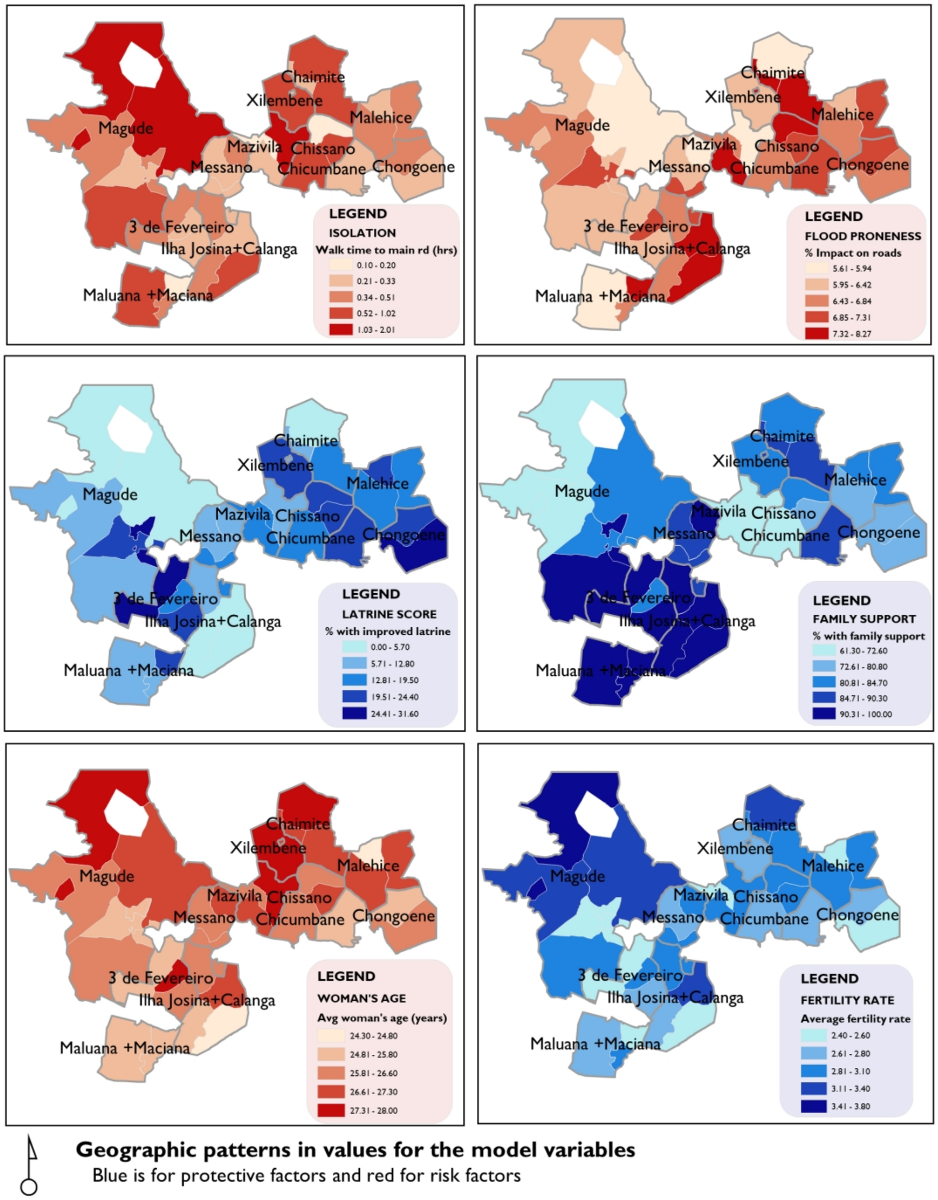

Figure 3: Geographic patterns in values for the model variables

169x217mm (300 x 300 DPI)

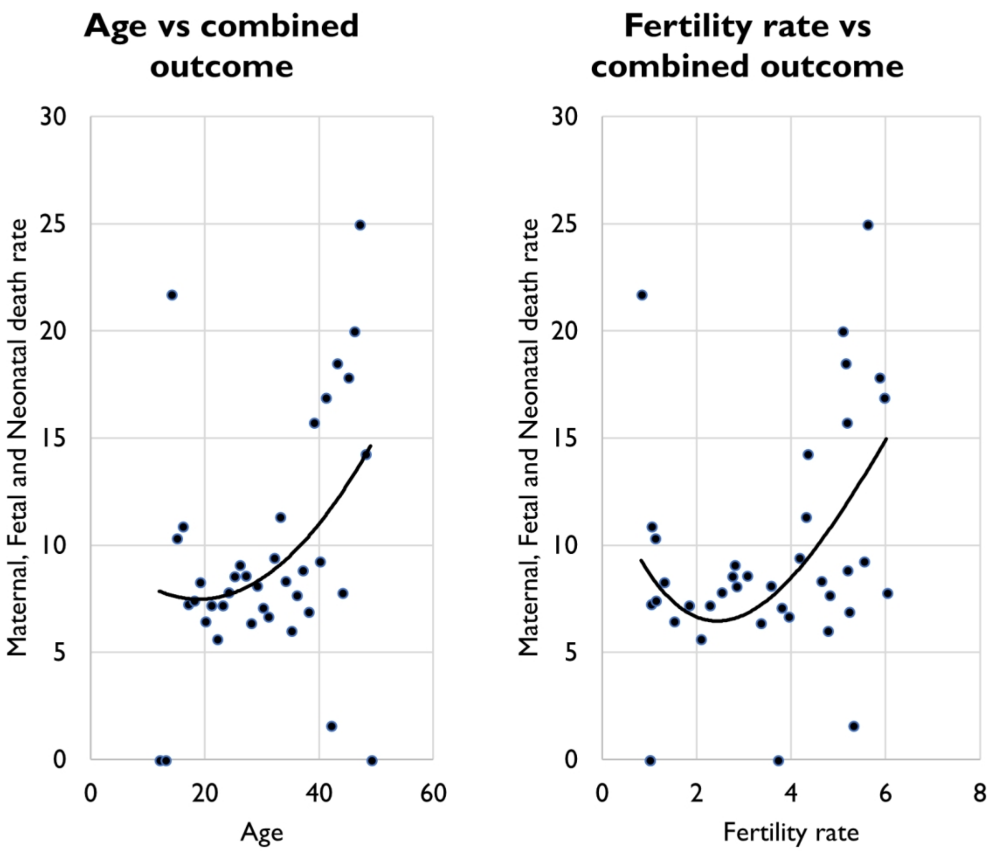

Figure 4: Age and fertility rates compared with adverse outcomes. The classic J shape graph for both

142x127mm (300 x 300 DPI)

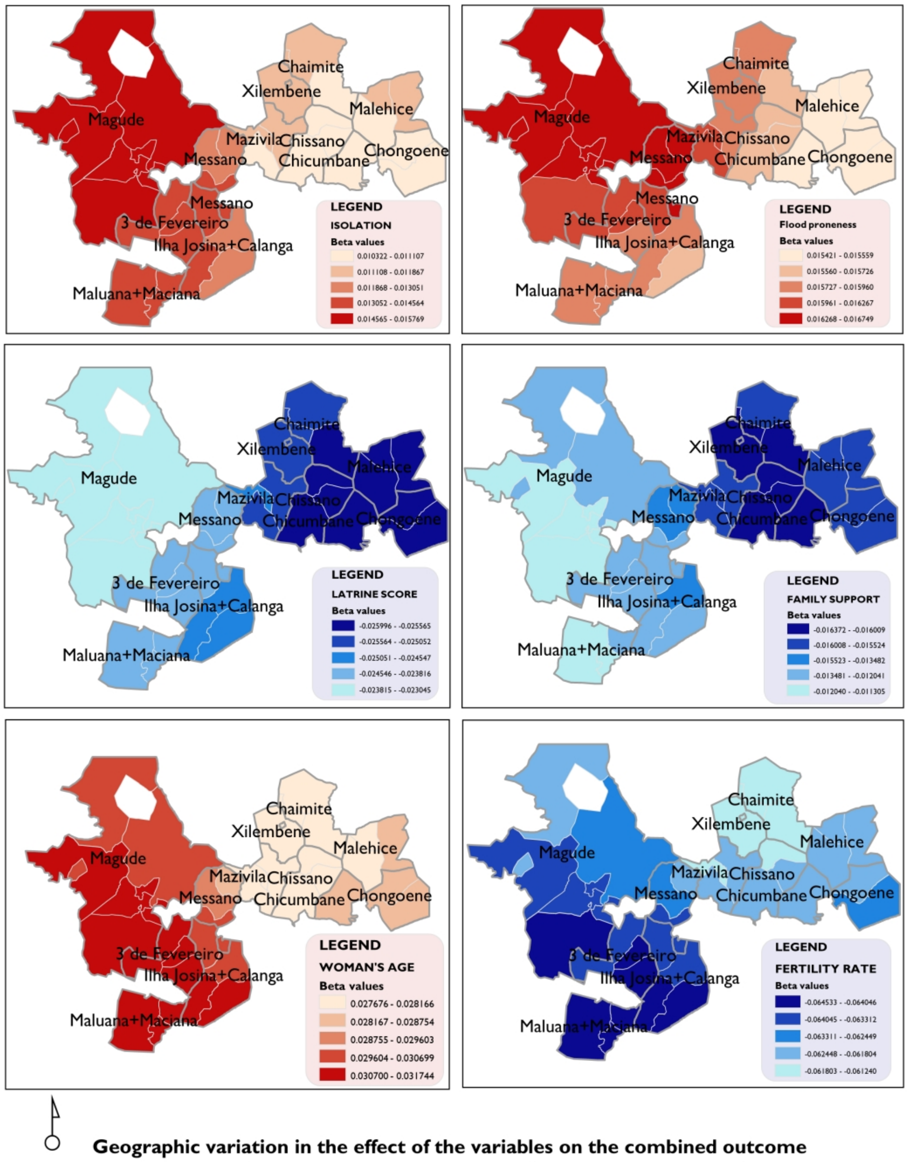

Geographic variation in the effect of the variables on the combined outcome
Darkening shade of blue indicates increasing protective effect while darkening shade of red indicates increasing risk effect.

Figure 5: Geographic variation of beta coefficients

211x279mm (300 x 300 DPI)

```
VARIABLE DEFINITIONS:

Variable        Variable Name
FERTRATE        Fertility rate
FAMSUPP_RT      Family support
WRAAGE_RT       Average age of reproductive age woman
UNEMP_RT        Reproductive age women's unemployment rate
ROQI_IMPACT     Flood proneness
PRVTRANS_RT     Private transportation score
LATRINE_RT      Latrine score
WALK_MRD        Isolation
HHH_AVAIL       Household head's availability
WATER_RT        Water source score
MARRIED_RT      Reproductive age women's marital status score
PUBDRV          Access to secondary health facilities
PUB                Access to primary health facilities
WRAEDUC         Reproductive age women's education
PUBDRVDRV       Access to tertiary health facilities
HHHEDUC         Household head's education
FINAUTPRE_RT    Financial autonomy in pregnancy
COMSUPP_RT      Community group support

******************************************************************************
Choose 1 of 18 Summary
     Highest Adjusted R-Squared Results
AdjR2    AICc   JB K(BP)  VIF   SA   Model
 0.16 -118.08 0.39  0.15 1.00 0.02  -FERTRATE***
 0.10 -115.69 0.18  0.76 1.00 0.04  +ROQI_IMPACT**
 0.08 -114.90 0.66  0.05 1.00 0.11  -UNEMP_RT**
       Passing Models
AdjR2 AICc JB K(BP) VIF SA   Model
******************************************************************************
Choose 2 of 18 Summary
            Highest Adjusted R-Squared Results
AdjR2    AICc   JB K(BP)  VIF   SA   Model
 0.35 -125.40 0.77  0.03 1.06 0.13  -UNEMP_RT***  -FERTRATE***
 0.29 -122.56 0.03  0.26 2.46 0.37  +WRAAGE_RT**  -FERTRATE***
 0.27 -121.23 0.91  0.35 1.05 0.01  -FAMSUPP_RT**  -FERTRATE***
       Passing Models
AdjR2 AICc JB K(BP) VIF SA   Model
******************************************************************************
Choose 3 of 18 Summary
                     Highest Adjusted R-Squared Results
AdjR2    AICc   JB K(BP)  VIF   SA   Model
 0.46 -130.66 0.26  0.64 3.68 0.71  -LATRINE_RT***  +WRAAGE_RT***
-FERTRATE***
 0.44 -129.33 0.56  0.55 1.50 0.08  -PRVTRANS_RT***  -FAMSUPP_RT***
-FERTRATE***
 0.44 -129.23 0.01  0.17 1.65 0.19  -LATRINE_RT***  -FAMSUPP_RT***
-FERTRATE***
       Passing Models
AdjR2 AICc JB K(BP) VIF SA   Model
******************************************************************************
Choose 4 of 18 Summary
                            Highest Adjusted R-Squared Results
AdjR2    AICc   JB K(BP)  VIF   SA   Model
 0.60 -139.23 0.53  0.04 3.69 0.64  -LATRINE_RT***  -FAMSUPP_RT***
+WRAAGE_RT***  -FERTRATE***
 0.52 -132.83 0.18  0.84 3.75 0.94  +ROQI_IMPACT**  -LATRINE_RT***
+WRAAGE_RT***  -FERTRATE***
```

```
 0.52 -132.54 0.46  0.82 1.65 0.15  +PUBDRV**  -PRVTRANS_RT***  -FAMSUPP_RT***
-FERTRATE***
                                                Passing Models
AdjR2    AICc        JB       K(BP)    VIF      SA         Model
0.602028 -139.231246 0.531046 0.039391 3.694394 0.643045  -LATRINE_RT***
-FAMSUPP_RT***  +WRAAGE_RT***  -FERTRATE***
0.522176 -132.831155 0.175090 0.844169 3.754031 0.939831  +ROQI_IMPACT**
-LATRINE_RT***  +WRAAGE_RT***  -FERTRATE***
0.518237 -132.543818 0.458059 0.818336 1.650636 0.148576  +PUBDRV**
-PRVTRANS_RT***  -FAMSUPP_RT***  -FERTRATE***
0.515228 -132.325907 0.781122 0.499719 3.851258 0.772143  -LATRINE_RT**
-UNEMP_RT**  +WRAAGE_RT***  -FERTRATE***
0.513983 -132.236119 0.325629 0.334396 1.612012 0.103168  -WATER_RT**
-PRVTRANS_RT***  -FAMSUPP_RT***  -FERTRATE***
0.510264 -131.969343 0.166281 0.246145 1.982255 0.420674  -LATRINE_RT**
-PRVTRANS_RT**  -FAMSUPP_RT***  -FERTRATE***
******************************************************************************
Choose 5 of 18 Summary
                                   Highest Adjusted R-Squared Results
AdjR2    AICc   JB K(BP)  VIF   SA   Model
 0.65 -141.61 0.54  0.02 3.77 0.67  +ROQI_IMPACT***  -LATRINE_RT***
-FAMSUPP_RT**  +WRAAGE_RT***  -FERTRATE***
 0.64 -140.61 0.63  0.35 3.91 0.98  +WALK_MRD***  +ROQI_IMPACT***
-LATRINE_RT***  +WRAAGE_RT***  -FERTRATE***
 0.63 -139.66 0.49  0.27 3.70 0.30  -LATRINE_RT***  -FAMSUPP_RT***
+WRAAGE_RT***  -FERTRATE***  +HHH_AVAIL*
                                                        Passing Models
AdjR2    AICc        JB       K(BP)    VIF      SA         Model
0.648492 -141.614920 0.536501 0.021780 3.774488 0.668519  +ROQI_IMPACT***
-LATRINE_RT***  -FAMSUPP_RT**  +WRAAGE_RT***  -FERTRATE***
0.638286 -140.613195 0.625085 0.345632 3.908376 0.976892  +WALK_MRD***
+ROQI_IMPACT***  -LATRINE_RT***  +WRAAGE_RT***  -FERTRATE***
0.615682 -138.491555 0.529587 0.076835 3.726831 0.442333  -LATRINE_RT***
+MARRIED_RT**  -FAMSUPP_RT***  +WRAAGE_RT***  -FERTRATE***
0.594995 -136.656610 0.367722 0.363750 3.541431 0.366122  +PUBDRV***
-PRVTRANS_RT***  -FAMSUPP_RT***  +WRAAGE_RT**  -FERTRATE***
0.582505 -135.593509 0.556140 0.003931 3.357147 0.143597  -WATER_RT***
-PRVTRANS_RT***  -FAMSUPP_RT***  +WRAAGE_RT**  -FERTRATE***
0.568548 -134.442595 0.687727 0.113630 3.178226 0.277763  +WALK_MRD***
+ROQI_IMPACT***  +MARRIED_RT**  +WRAAGE_RT***  -FERTRATE***
0.564733 -134.134426 0.626979 0.464601 3.857918 0.255002  -LATRINE_RT**
-UNEMP_RT**  +WRAAGE_RT***  -FERTRATE***  +HHH_AVAIL**
0.563092 -134.002788 0.353655 0.096116 3.259366 0.415519  +PUBDRVDRV**
-PRVTRANS_RT***  -FAMSUPP_RT***  +WRAAGE_RT**  -FERTRATE***
0.552392 -133.155941 0.795824 0.613240 3.500445 0.197948  +PUBDRV***
+ROQI_IMPACT**  -UNEMP_RT**  +WRAAGE_RT***  -FERTRATE***
0.523659 -130.978388 0.575476 0.009866 3.319401 0.552068  -WATER_RT***
+MARRIED_RT**  -FAMSUPP_RT**  +WRAAGE_RT**  -FERTRATE***
******************************************************************************
Choose 6 of 18 Summary
                                          Highest Adjusted R-Squared Results
AdjR2    AICc   JB K(BP)  VIF   SA   Model
 0.69 -144.18 0.38  0.55 3.91 0.92  +WALK_MRD**  +ROQI_IMPACT***
-LATRINE_RT***  -FAMSUPP_RT**  +WRAAGE_RT***  -FERTRATE***
 0.67 -142.16 0.70  0.10 3.78 0.21  +ROQI_IMPACT***  -LATRINE_RT***
-FAMSUPP_RT**  +WRAAGE_RT***  -FERTRATE***  +HHH_AVAIL**
 0.67 -141.32 0.49  0.49 4.07 0.94  +PUBDRV  +ROQI_IMPACT**  -LATRINE_RT***
-FAMSUPP_RT***  +WRAAGE_RT***  -FERTRATE***
                                                               Passing Models
AdjR2    AICc        JB       K(BP)    VIF      SA         Model
```

```
0.692863 -144.175618 0.379263 0.549371 3.908531 0.923074  +WALK_MRD**
+ROQI_IMPACT***  -LATRINE_RT***  -FAMSUPP_RT**  +WRAAGE_RT***  -FERTRATE***
0.674630 -142.157235 0.695520 0.098209 3.778934 0.213202  +ROQI_IMPACT***
-LATRINE_RT***  -FAMSUPP_RT**  +WRAAGE_RT***  -FERTRATE***  +HHH_AVAIL**
0.635329 -138.166134 0.568820 0.898220 3.550214 0.205750  +PUBDRV***
+ROQI_IMPACT**  -PRVTRANS_RT***  -FAMSUPP_RT***  +WRAAGE_RT***  -FERTRATE***
0.598989 -134.841346 0.651598 0.008895 3.478494 0.553360  -WATER_RT***
-PRVTRANS_RT**  +MARRIED_RT**  -FAMSUPP_RT***  +WRAAGE_RT**  -FERTRATE***
0.597255 -134.690338 0.545878 0.001749 3.326038 0.523384  +WALK_MRD**
-PRVTRANS_RT***  +MARRIED_RT**  -FAMSUPP_RT***  +WRAAGE_RT**  -FERTRATE***
0.592810 -134.306174 0.781534 0.033482 3.269749 0.690837  +ROQI_IMPACT***
-WATER_RT***  -FAMSUPP_RT**  +WRAAGE_RT***  -FERTRATE***  +HHH_AVAIL**
******************************************************************************
Choose 7 of 18 Summary
                                                 Highest Adjusted R-Squared
Results
AdjR2    AICc   JB K(BP)  VIF   SA   Model
 0.71 -143.42 0.43  0.45 3.98 0.79  +WALK_MRD**  +ROQI_IMPACT***
-LATRINE_RT***  +MARRIED_RT  -FAMSUPP_RT**  +WRAAGE_RT***  -FERTRATE***
 0.70 -143.13 0.47  0.47 3.93 0.45  +WALK_MRD*  +ROQI_IMPACT***
-LATRINE_RT***  -FAMSUPP_RT**  +WRAAGE_RT***  -FERTRATE***  +HHH_AVAIL
 0.70 -142.49 0.35  0.68 4.31 0.64  +WALK_MRD**  +ROQI_IMPACT**
-LATRINE_RT***  -PRVTRANS_RT  -FAMSUPP_RT***  +WRAAGE_RT***  -FERTRATE***
                                                                     Passing
Models
AdjR2    AICc        JB       K(BP)     VIF      SA        Model
0.645867 -136.803684 0.761213 0.081722 3.329961 0.135850  +WALK_MRD***
+ROQI_IMPACT**  -PRVTRANS_RT***  +MARRIED_RT**  -FAMSUPP_RT**  +WRAAGE_RT***
-FERTRATE***
******************************************************************************
Choose 8 of 18 Summary
                                                       Highest Adjusted
R-Squared Results
AdjR2    AICc   JB K(BP)  VIF   SA   Model
 0.71 -141.63 0.41  0.29 4.38 0.96  +WALK_MRD**  +ROQI_IMPACT**  -LATRINE_RT**
-PRVTRANS_RT  +MARRIED_RT  -FAMSUPP_RT***  +WRAAGE_RT***  -FERTRATE***
 0.71 -141.02 0.43  0.39 4.04 0.43  +WALK_MRD**  +ROQI_IMPACT***
-LATRINE_RT***  +MARRIED_RT  -FAMSUPP_RT**  +WRAAGE_RT***  -FERTRATE***
+HHH_AVAIL
 0.70 -140.48 0.54  0.63 4.31 0.66  +WALK_MRD**  +ROQI_IMPACT**
-LATRINE_RT***  -PRVTRANS_RT  -FAMSUPP_RT**  +WRAAGE_RT***  -FERTRATE***
+HHH_AVAIL
       Passing Models
AdjR2 AICc JB K(BP) VIF SA   Model
******************************************************************************
Choose 9 of 18 Summary
                                                             Highest Adjusted
R-Squared Results
AdjR2    AICc   JB K(BP)  VIF   SA   Model
 0.71 -138.32 0.47  0.33 4.39 0.63  +WALK_MRD**  +ROQI_IMPACT**
-LATRINE_RT***  -PRVTRANS_RT  +MARRIED_RT  -FAMSUPP_RT**  +WRAAGE_RT***
-FERTRATE***  +HHH_AVAIL
 0.71 -138.12 0.43  0.35 4.38 0.68  +WALK_MRD**  +ROQI_IMPACT**
-LATRINE_RT***  -PRVTRANS_RT  +MARRIED_RT  -FAMSUPP_RT**  +WRAAGE_RT***
-FERTRATE***  +WRAEDUC
 0.71 -138.07 0.43  0.56 4.41 0.95  +WALK_MRD**  +PUBDRVDRV  +ROQI_IMPACT**
-LATRINE_RT**  -PRVTRANS_RT  +MARRIED_RT  -FAMSUPP_RT***  +WRAAGE_RT***
-FERTRATE***
       Passing Models
AdjR2 AICc JB K(BP) VIF SA   Model
```

For peer review only

```
******************************************************************************
Choose 10 of 18 Summary
                                                                       Highest
Adjusted R-Squared Results
AdjR2    AICc   JB K(BP)  VIF   SA   Model
 0.71 -135.47 0.51  0.49 6.61 0.80  +WALK_MRD***  +ROQI_IMPACT**
-LATRINE_RT**  -PRVTRANS_RT*  +MARRIED_RT*  -FAMSUPP_RT**  +WRAAGE_RT***
-FERTRATE***  -HHHEDUC  +WRAEDUC
 0.71 -134.98 0.43  0.43 4.39 0.80  +WALK_MRD**  +ROQI_IMPACT**
-LATRINE_RT***  -PRVTRANS_RT  +MARRIED_RT  -FAMSUPP_RT**  +WRAAGE_RT***
-FERTRATE***  +HHH_AVAIL  +WRAEDUC
 0.71 -134.76 0.44  0.59 4.67 0.74  +WALK_MRD**  +PUBDRVDRV  +ROQI_IMPACT**
-LATRINE_RT**  -PRVTRANS_RT  +MARRIED_RT  -FAMSUPP_RT***  +FINAUTPRE_RT
+WRAAGE_RT***  -FERTRATE***
       Passing Models
AdjR2 AICc JB K(BP) VIF SA   Model
******************************************************************************
************** Exploratory Regression Global Summary (NEW_OUTC) **************

              Percentage of Search Criteria Passed
                   Search Criterion Cutoff Trials # Passed % Passed
             Min Adjusted R-Squared > 0.50 199139    25678    12.89
            Max Coefficient p-value < 0.05 199139       61     0.03
                      Max VIF Value < 4.50 199139    99568    50.00
            Min Jarque-Bera p-value > 0.10 199139   191512    96.17
Min Spatial Autocorrelation p-value > 0.10     51       43    84.31

------------------------------------------------------------------------------

        Summary of Variable Significance
Variable        % Significant % Negative % Positive
FERTRATE                99.82     100.00      0.00
FAMSUPP_RT              44.71      99.88      0.12
WRAAGE_RT               43.32      41.63     58.37
UNEMP_RT                27.99      92.68      7.32
ROQI_IMPACT             23.78       0.00    100.00
PRVTRANS_RT             19.97      94.84      5.16
LATRINE_RT              14.39      67.86     32.14
WALK_MRD                10.09       5.22     94.78
HHH_AVAIL                3.28       2.27     97.73
WATER_RT                 2.87      74.80     25.20
MARRIED_RT               2.77      19.79     80.21
PUBDRV                   1.74      29.98     70.02
PUB                           1.39      81.69      18.31
WRAEDUC                  0.86      30.60     69.40
PUBDRVDRV                0.79      60.09     39.91
HHHEDUC                  0.24      34.71     65.29
FINAUTPRE_RT             0.10      92.44      7.56
COMSUPP_RT               0.01      45.72     54.28

------------------------------------------------------------------------------


Summary of Multicollinearity
Variable          VIF Violations Covariates
PUB                 8.80   34028    PUBDRV (77.21), PUBDRVDRV (31.02),
HHHEDUC (12.71), WRAEDUC (9.33), LATRINE_RT (6.14), FERTRATE (4.87), WALK_MRD
(3.30), WATER_RT (3.03), FAMSUPP_RT (0.12), WRAAGE_RT (0.03), PRVTRANS_RT
(0.02)
WALK_MRD         5.89    1610    PUB (3.30), PUBDRVDRV (2.98), PUBDRV (2.34),
```

For peer review only

```
WATER_RT (0.44), HHHEDUC (0.21), WRAEDUC (0.21), LATRINE_RT (0.11), FERTRATE
(0.05), PRVTRANS_RT (0.02), FAMSUPP_RT (0.01)
PUBDRV          14.41   63274    PUBDRVDRV (90.54), PUB (77.21), HHHEDUC
(22.77), WRAEDUC (16.51), LATRINE_RT (12.61), FERTRATE (6.57), WATER_RT
(6.36), WALK_MRD (2.34), FAMSUPP_RT (0.12), WRAAGE_RT (0.03), PRVTRANS_RT
(0.01)
PUBDRVDRV        8.68   37253    PUBDRV (90.54), PUB (31.02), HHHEDUC (14.17),
WRAEDUC (10.57), LATRINE_RT (7.69), WATER_RT (5.32), FERTRATE (3.03), WALK_MRD
(2.98), FAMSUPP_RT (0.02), PRVTRANS_RT (0.01), WRAAGE_RT (0.01)
ROQI_IMPACT      1.94     0      --------
WATER_RT         5.29    6578    LATRINE_RT (7.08), PUBDRV (6.36), PUBDRVDRV
(5.32), PUB (3.03), HHHEDUC (2.00), WRAEDUC (1.66), FERTRATE (1.13), WALK_MRD
(0.44), FAMSUPP_RT (0.00), WRAAGE_RT (0.00)
LATRINE_RT       6.10   13218    PUBDRV (12.61), PUBDRVDRV (7.69), WATER_RT
(7.08), HHHEDUC (7.08), WRAEDUC (6.79), PUB (6.14), FERTRATE (5.99), WALK_MRD
(0.11), WRAAGE_RT (0.02)
PRVTRANS_RT      5.04     8      PUB (0.02), WALK_MRD (0.02), PUBDRVDRV
(0.01), PUBDRV (0.01), FAMSUPP_RT (0.00)
MARRIED_RT       1.68     0      --------
UNEMP_RT         3.69     0      --------
FAMSUPP_RT       5.19     54     PUBDRV (0.12), PUB (0.12), HHHEDUC (0.04),
WRAEDUC (0.04), PUBDRVDRV (0.02), WALK_MRD (0.01), WATER_RT (0.00),
PRVTRANS_RT (0.00)
COMSUPP_RT       1.86     0      --------
FINAUTPRE_RT     2.26     0      --------
WRAAGE_RT        4.75     38     HHHEDUC (0.10), WRAEDUC (0.10), FERTRATE
(0.09), PUB (0.03), PUBDRV (0.03), LATRINE_RT (0.02), PUBDRVDRV (0.01),
WATER_RT (0.00)
FERTRATE         5.42    8320    PUBDRV (6.57), LATRINE_RT (5.99), PUB (4.87),
WRAEDUC (3.78), HHHEDUC (3.69), PUBDRVDRV (3.03), WATER_RT (1.13), WRAAGE_RT
(0.09), WALK_MRD (0.05)
HHH_AVAIL        2.62     0      --------
HHHEDUC          8.78   28637    WRAEDUC (58.81), PUBDRV (22.77), PUBDRVDRV
(14.17), PUB (12.71), LATRINE_RT (7.08), FERTRATE (3.69), WATER_RT (2.00),
WALK_MRD (0.21), WRAAGE_RT (0.10), FAMSUPP_RT (0.04)
WRAEDUC          8.89   24552    HHHEDUC (58.81), PUBDRV (16.51), PUBDRVDRV
(10.57), PUB (9.33), LATRINE_RT (6.79), FERTRATE (3.78), WATER_RT (1.66),
WALK_MRD (0.21), WRAAGE_RT (0.10), FAMSUPP_RT (0.04)

------------------------------------------------------------------------------


Summary of Residual Normality (JB)
      JB    AdjR2        AICc      K(BP)       VIF       SA    Model
0.999981 0.011731  -92.024434 0.002361 4.176647 0.053210  -PUB  +WALK_MRD
-PUBDRVDRV  +ROQI_IMPACT  -WATER_RT  +MARRIED_RT  -FAMSUPP_RT  +COMSUPP_RT
-WRAAGE_RT  +HHHEDUC
0.999979 0.520437 -123.545871 0.883158 4.638126 0.884202  +PUBDRV
-LATRINE_RT*  -UNEMP_RT*  -FINAUTPRE_RT  +WRAAGE_RT***  -FERTRATE***  -HHHEDUC
+WRAEDUC
0.999975 0.099522 -106.528531 0.002699 2.039475 0.005442  -PUBDRVDRV
+ROQI_IMPACT*  -WATER_RT  -FAMSUPP_RT  -WRAAGE_RT  +HHHEDUC

------------------------------------------------------------------------------

                                                              Summary of
Residual Spatial Autocorrelation (SA)
      SA    AdjR2        AICc       JB     K(BP)       VIF    Model
0.976892 0.638286 -140.613195 0.625085 0.345632 3.908376  +WALK_MRD***
+ROQI_IMPACT***  -LATRINE_RT***  +WRAAGE_RT***  -FERTRATE***
```

For peer review only

```
0.958004 0.713906 -141.625323 0.409253 0.288293 4.380150  +WALK_MRD**
+ROQI_IMPACT**  -LATRINE_RT**  -PRVTRANS_RT  +MARRIED_RT  -FAMSUPP_RT***
+WRAAGE_RT***  -FERTRATE***
0.954297 0.708834 -138.071313 0.431612 0.559128 4.414522  +WALK_MRD**
+PUBDRVDRV  +ROQI_IMPACT**  -LATRINE_RT**  -PRVTRANS_RT  +MARRIED_RT
-FAMSUPP_RT***  +WRAAGE_RT***  -FERTRATE***

------------------------------------------------------------------------------

Table Abbreviations
AdjR2 Adjusted R-Squared
AICc  Akaike's Information Criterion
JB    Jarque-Bera p-value
K(BP) Koenker (BP) Statistic p-value
VIF   Max Variance Inflation Factor
SA    Global Moran's I p-value
Model Variable sign (+/-)
Model Variable significance (* = 0.10, ** = 0.05, *** = 0.01)

------------------------------------------------------------------------------
```

For peer review only

Summary of OLS Results - Model Variables

Variable	Coefficient [a]	StdError	t-Statistic	Probability [b]	Robust_SE	Robust_t	Robust_Pr [b]	VIF [c]
Intercept	-0.194483	0.198020	-0.982142	0.334442	0.198192	-0.981289	0.334855	--------
WALK_MRD	0.033353	0.014641	2.278046	0.030557*	0.015198	2.194592	0.036652*	2.065976
ROQI_IMPACT	0.023936	0.007537	3.175867	0.003620*	0.006860	3.489274	0.001620*	1.353110
LATRINE_RT	-0.003094	0.000722	-4.284566	0.000194*	0.000677	-4.572118	0.000088*	1.923468
FAMSUPP_RT	-0.001274	0.000514	-2.480549	0.019399*	0.000602	-2.115857	0.043382*	1.526477
WRAAGE_RT	0.034653	0.008260	4.195438	0.000247*	0.008113	4.271314	0.000201*	2.933353
FERTRATE	-0.222607	0.029423	-7.565753	0.000000*	0.030839	-7.218404	0.000000*	3.908531

OLS Diagnostics

Input Features:	LOCALITY_COMP_PREG	Dependent Variable:	NEW_OUTC
Number of Observations:	35	Akaike's Information Criterion (AICc) [d]:	-144.175618
Multiple R-Squared [d]:	0.747063	Adjusted R-Squared [d]:	0.692863
Joint F-Statistic [e]:	13.783283	Prob(>F), (6,28) degrees of freedom:	0.000000*
Joint Wald Statistic [e]:	139.475044	Prob(>chi-squared), (6) degrees of freedom:	0.000000*
Koenker (BP) Statistic [f]:	4.956762	Prob(>chi-squared), (6) degrees of freedom:	0.549371
Jarque-Bera Statistic [g]:	1.939051	Prob(>chi-squared), (2) degrees of freedom:	0.379263

Notes on Interpretation

* An asterisk next to a number indicates a statistically significant p-value (p < 0.01).

[a] Coefficient: Represents the strength and type of relationship between each explanatory variable and the dependent variable.

[b] Probability and Robust Probability (Robust_Pr): Asterisk (*) indicates a coefficient is statistically significant (p < 0.01); if the Koenker (BP) Statistic [f] is statistically significant, use the Robust Probability column (Robust_Pr) to determine coefficient significance.

[c] Variance Inflation Factor (VIF): Large Variance Inflation Factor (VIF) values (> 7.5) indicate redundancy among explanatory variables.

[d] R-Squared and Akaike's Information Criterion (AICc): Measures of model fit/performance.

[e] Joint F and Wald Statistics: Asterisk (*) indicates overall model significance (p < 0.01); if the Koenker (BP) Statistic [f] is statistically significant, use the Wald Statistic to determine overall model significance.

[f] Koenker (BP) Statistic: When this test is statistically significant (p < 0.01), the relationships modeled are not consistent (either due to non-stationarity or heteroskedasticity). You should rely on the Robust Probabilities (Robust_Pr) to determine coefficient significance and on the Wald Statistic to determine overall model significance.

[g] Jarque-Bera Statistic: When this test is statistically significant (p < 0.01) model predictions are biased (the residuals are not normally distributed).

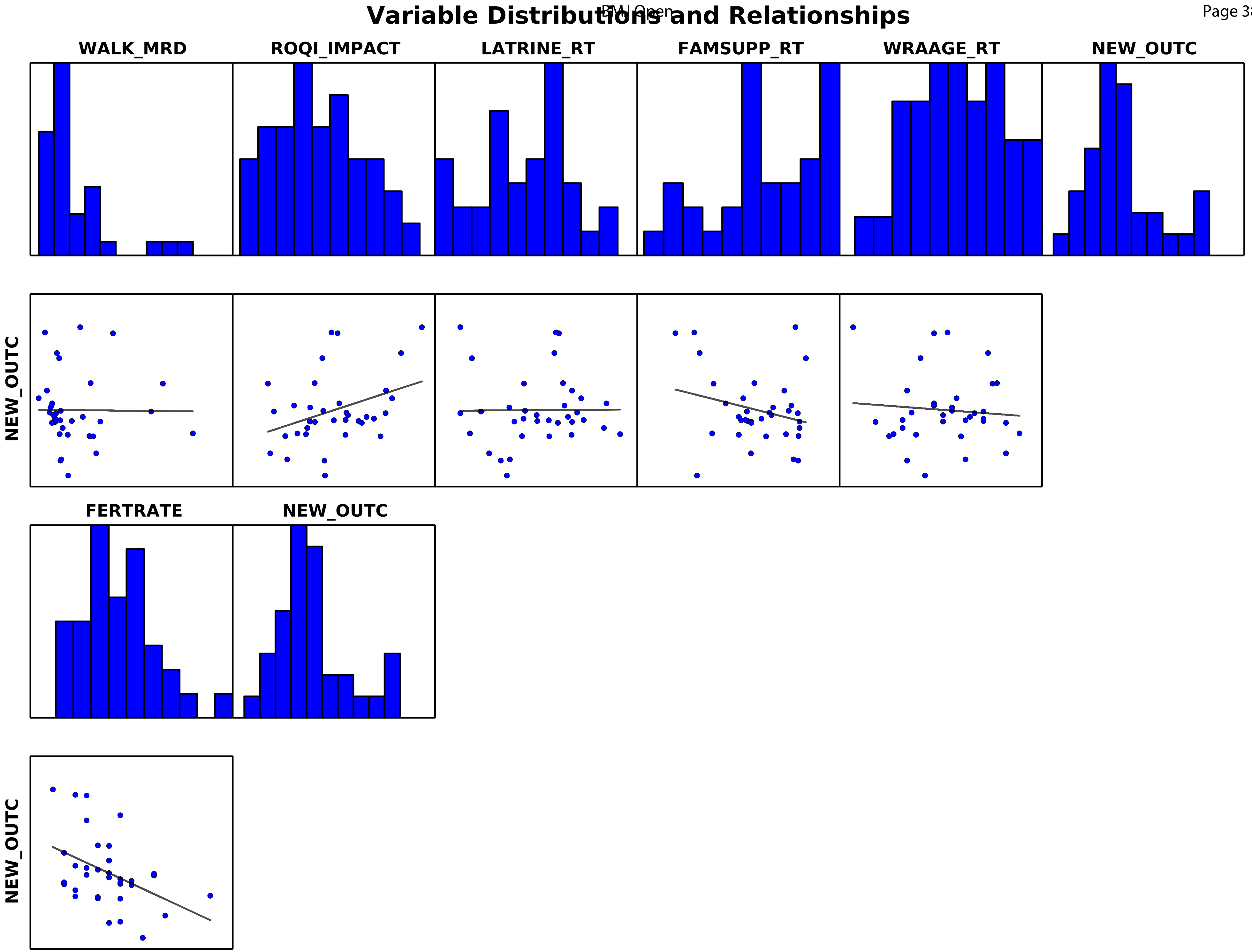
Variable Distributions and Relationships
WALK_MRD
ROQI_IMPACT
LATRINE_RT
FAMSUPP_RT
WRAAGE_RT
NEW_OUTC
NEW_OUTC
FERTRATE
NEW_OUTC
NEW_OUTC

Variable Distributions and Relationships (Cont.)

The above graphs are Histograms and Scatterplots for each explanatory variable and the dependent variable. The histograms show how each variable is distributed. OLS does not require variables to be normally distributed. However, if you are having trouble finding a properly-specified model, you can try transforming strongly skewed variables to see if you get a better result.

Each scatterplot depicts the relationship between an explanatory variable and the dependent variable. Strong relationships appear as diagonals and the direction of the slant indicates if the relationship is positive or negative. Try transforming your variables if you detect any non-linear relationships. For more information see the Regression Analysis Basics documentation.

Histogram of Standardized Residuals

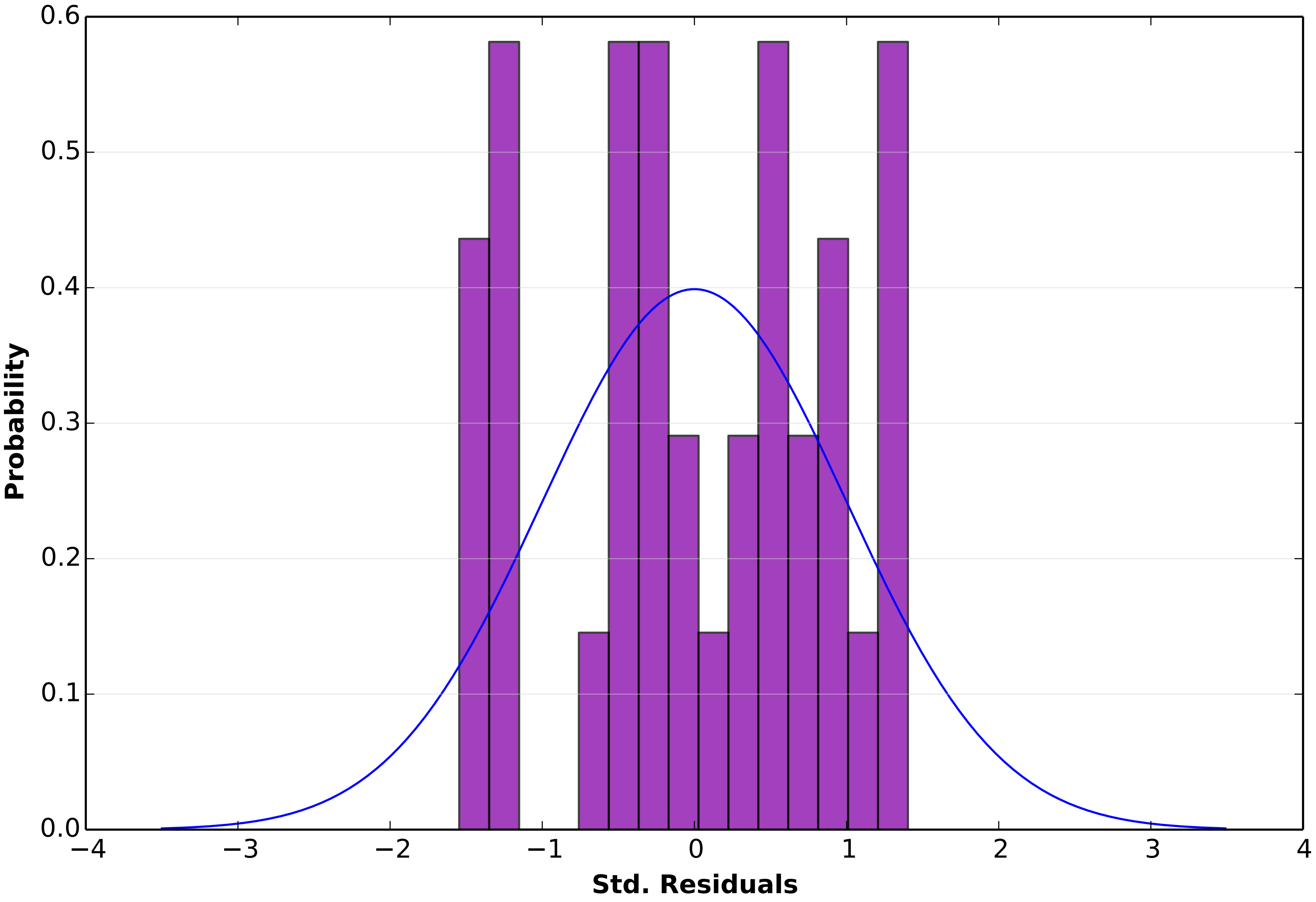

Ideally the histogram of your residuals would match the normal curve, indicated above in blue. If the histogram looks very different from the normal curve, you may have a biased model. If this bias is significant it will also be represented by a statistically significant Jarque-Bera p-value (*).

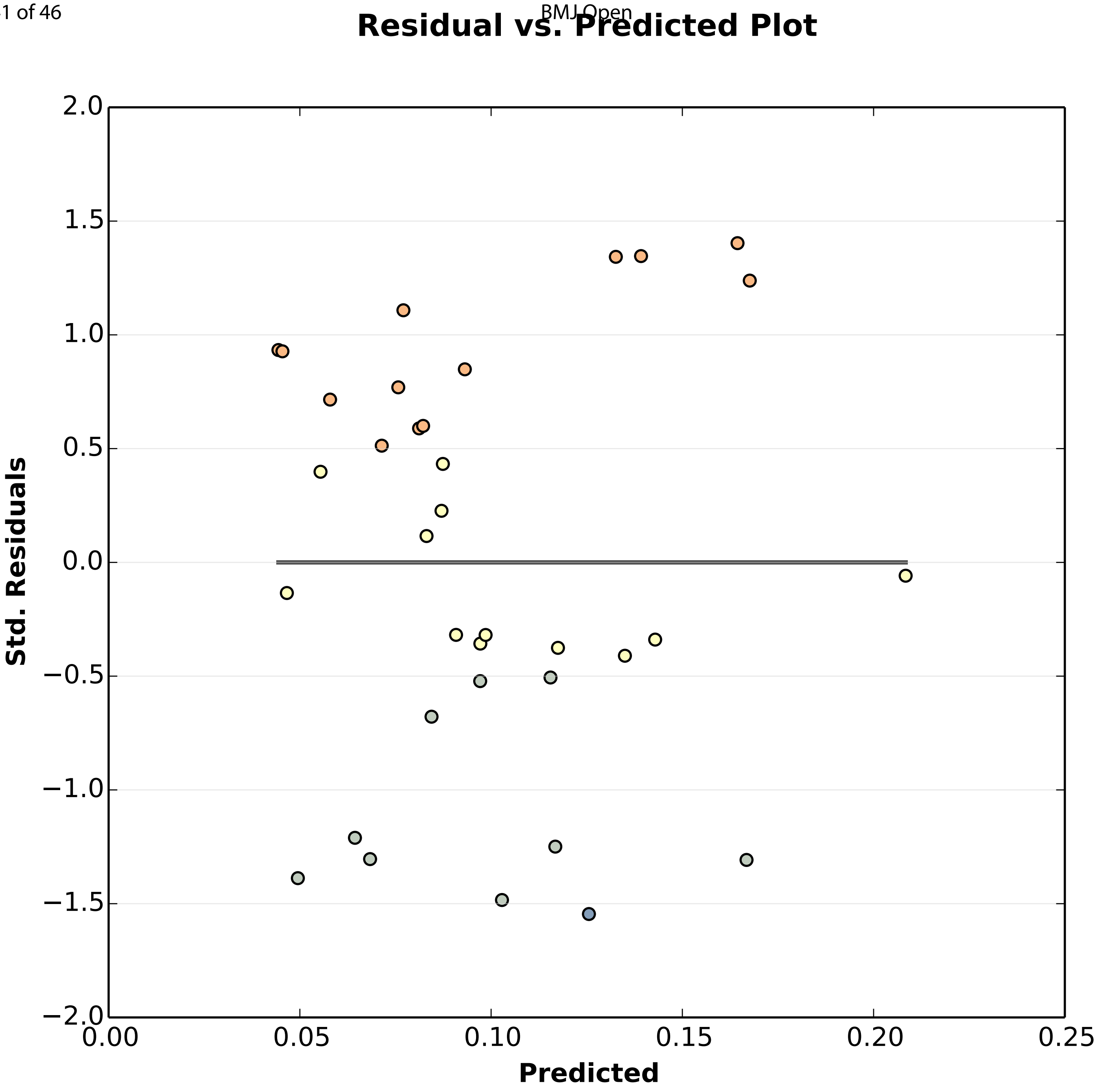

This is a graph of residuals (model over and under predictions) in relation to predicted dependent variable values. For a properly specified model, this scatterplot will have little structure, and look random (see graph on the right). If there is a structure to this plot, the type of structure may be a valuable clue to help you figure out what's going on.

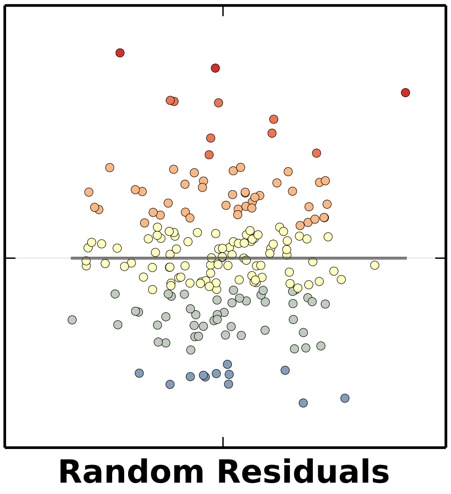

Ordinary Least Squares Parameters

Parameter Name	Input Value
Input Features	LOCALITY_COMP_PREG
Unique ID Field	UNI_ID
Output Feature Class	None
Dependent Variable	NEW_OUTC
Explanatory Variables	WALK_MRD
	ROQI_IMPACT
	LATRINE_RT
	FAMSUPP_RT
	WRAAGE_RT
	FERTRATE
Selection Set	False

```
***************************************************************************
*           Semiparametric Geographically Weighted Regression             *
*                   Release 1.0.90 (GWR 4.0.90)                           *
*                          12 May 2015                                    *
*              (Originally coded by T. Nakaya: 1 Nov 2009)               *
*                                                                         *
*           Tomoki Nakaya(1), Martin Charlton(2), Chris Brunsdon (2)     *
*           Paul Lewis (2), Jing Yao (3), A Stewart Fotheringham (4)     *
*                    (c) GWR4 development team                           *
* (1) Ritsumeikan University, (2) National University of Ireland, Maynooth, *
*        (3) University of Glasgow, (4) Arizona State University         *
***************************************************************************

Program began at 2018-04-07 9:13:44 AM

***************************************************************************
Session: MOZ_GWR_2018
Session control file:
C:\Users\Prestige\OneDrive\CANADA\UBC\PRE-EMPT\MOM\Papers\Spatial EPI of
Maternal and Neonatal deaths\DATA\GWR_moz_2018.ctl
***************************************************************************
Data filename:
C:\Users\Prestige\OneDrive\CANADA\UBC\PRE-EMPT\MOM\Papers\Spatial EPI of
Maternal and Neonatal deaths\ForGWR.csv
Number of areas/points: 35

Model settings---------------------------------
Model type: Gaussian
Geographic kernel: adaptive Gaussian
Method for optimal bandwidth search: fixed value
Criterion for optimal bandwidth: AICc
Number of varying coefficients: 7
Number of fixed coefficients:   0

Modelling options---------------------------------
Standardisation of independent variables: On
Testing geographical variability of local coefficients: On
Local to Global Variable selection: OFF
Global to Local Variable selection: OFF
Prediction at non-regression points: On

Variable settings---------------------------------
Area key: field1: OBJECTID
Easting (x-coord): field14 : POINT_X
Northing (y-coord): field15: POINT_Y
Cartesian coordinates: Euclidean distance
Dependent variable: field3: NEW_OUTC
Offset variable is not specified
Intercept: varying (Local) intercept
Independent variable with varying (Local) coefficient: field4: WALK_MRD
Independent variable with varying (Local) coefficient: field5: RoQI_IMPACT
Independent variable with varying (Local) coefficient: field6: LATRINE_RT
Independent variable with varying (Local) coefficient: field7: FAMSUPP_RT
Independent variable with varying (Local) coefficient: field8: WRAAGE_RT
Independent variable with varying (Local) coefficient: field9: FERTRATE
Number of prediction at non-regression points   98
***************************************************************************

***************************************************************************
  Global regression result
```

```
***************************************************************************
  < Diagnostic information >
Residual sum of squares:                     0.018003
Number of parameters:                         7
 (Note: this num does not include an error variance term for a Gaussian model)
ML based global sigma estimate:              0.022680
Unbiased global sigma estimate:              0.025357
-2 log-likelihood:                        -165.714080
Classic AIC:                              -149.714080
AICc:                                     -144.175618
BIC/MDL:                                  -137.271295
CV:                                          0.000937
R square:                                    0.747063
Adjusted R square:                           0.681487

Variable                  Estimate    Standard Error      t(Est/SE)
-------------------- --------------- --------------- ---------------
Intercept                   0.100460        0.004298       23.374311
WALK_MRD                    0.014010        0.006150        2.278046
RoQI_IMPACT                 0.015719        0.004950        3.175867
LATRINE_RT                 -0.024529        0.005725       -4.284566
FAMSUPP_RT                 -0.012849        0.005180       -2.480549
WRAAGE_RT                   0.030268        0.007214        4.195438
FERTRATE                   -0.063174        0.008350       -7.565753

***************************************************************************
  GWR (Geographically weighted regression) bandwidth selection
***************************************************************************

Bandwidth search <fixed value>:  30

***************************************************************************
  GWR (Geographically weighted regression) result
***************************************************************************
  Bandwidth and geographic ranges
Bandwidth size:                  30.000000
Coordinate                Min             Max           Range
--------------- --------------- --------------- ---------------
X-coord          3616601.398000  3768895.758000   152294.360000
Y-coord         -2920426.694000 -2791357.504000   129069.190000

  Diagnostic information
Residual sum of squares:                0.015258
Effective number of parameters (model: trace(S)):                    8.181591
Effective number of parameters (variance: trace(S'S)):               7.198744
Degree of freedom (model: n - trace(S)):                            26.818409
Degree of freedom (residual: n - 2trace(S) + trace(S'S)):           25.835562
ML based sigma estimate:                0.020880
Unbiased sigma estimate:                0.024302
-2 log-likelihood:                   -171.503374
Classic AIC:                         -153.140192
AICc:                                -145.606817
BIC/MDL:                             -138.859623
CV:                                     0.000880
R square:                               0.785624
Adjusted R square:                      0.706519

***********************************************************
 << Geographically varying (Local) coefficients >>
***********************************************************
```

For peer review only

Estimates of varying coefficients have been saved in the following file.
Listwise output file:
C:\Users\Prestige\OneDrive\CANADA\UBC\PRE-EMPT\MOM\Papers\Spatial EPI of Maternal and Neonatal deaths\DATA\GWR_moz_2018_listwise.csv

Summary statistics for varying (Local) coefficients

Variable	Mean	STD
Intercept	0.101035	0.000664
WALK_MRD	0.012786	0.001721
RoQI_IMPACT	0.015974	0.000361
LATRINE_RT	-0.024667	0.000923
FAMSUPP_RT	-0.014028	0.001883
WRAAGE_RT	0.029602	0.001537
FERTRATE	-0.062852	0.001075

Variable	Min	Max	Range
Intercept	0.099979	0.101892	0.001913
WALK_MRD	0.010322	0.015769	0.005447
RoQI_IMPACT	0.015421	0.016749	0.001328
LATRINE_RT	-0.025996	-0.023045	0.002951
FAMSUPP_RT	-0.016372	-0.011305	0.005068
WRAAGE_RT	0.027676	0.031744	0.004068
FERTRATE	-0.064533	-0.061240	0.003293

Variable	Lwr Quartile	Median	Upr Quartile
Intercept	0.100351	0.101371	0.101613
WALK_MRD	0.011107	0.012520	0.014249
RoQI_IMPACT	0.015689	0.015875	0.016267
LATRINE_RT	-0.025567	-0.024590	-0.024033
FAMSUPP_RT	-0.015926	-0.013778	-0.012218
WRAAGE_RT	0.028076	0.029603	0.031222
FERTRATE	-0.063924	-0.062449	-0.061996

Variable	Interquartile R	Robust STD
Intercept	0.001262	0.000936
WALK_MRD	0.003142	0.002329
RoQI_IMPACT	0.000579	0.000429
LATRINE_RT	0.001534	0.001137
FAMSUPP_RT	0.003708	0.002748
WRAAGE_RT	0.003146	0.002332
FERTRATE	0.001927	0.001429

(Note: Robust STD is given by (interquartile range / 1.349))

****
GWR ANOVA Table
****

Source	SS	DF	MS	F
Global Residuals	0.018	28.000		
GWR Improvement	0.003	2.164	0.001	
GWR Residuals	0.015	25.836	0.001	2.147056

****
Geographical variability tests of local coefficients
****

Variable	F	DOF for F test	DIFF of Criterion

For peer review only

Intercept	2.000828	0.142	26.818	0.176197
WALK_MRD	7.975741	0.195	26.818	-1.224025
RoQI_IMPACT	0.748443	0.185	26.818	0.530483
LATRINE_RT	3.943637	0.125	26.818	-0.156581
FAMSUPP_RT	15.587840	0.164	26.818	-2.551618
WRAAGE_RT	2.703057	0.215	26.818	0.073263
FERTRATE	2.022504	0.157	26.818	0.190761

Note: positive value of diff-Criterion (AICc, AIC, BIC/MDL or CV) suggests no spatial variability in terms of model selection criteria.
F test: in case of no spatial variability, the F statistics follows the F distribution of DOF for F test.

****
Program terminated at 2018-04-07 9:13:45 AM

For peer review only

BMJ Open

The place-specific factors associated with adverse maternal, and perinatal outcomes in southern Mozambique: A retrospective cohort study

Journal:	*BMJ Open*
Manuscript ID	bmjopen-2018-024042.R1
Article Type:	Research
Date Submitted by the Author:	23-Oct-2018
Complete List of Authors:	Makanga, Prestige; Midlands State University Faculty of Science and Technology, Surveying and Geomatics<br>Sacoor, Charfudin; Centro de Investigacao em Saude de Manhica, Department of Demography<br>Schuurman, Nadine; Simon Fraser Univ, Geography<br>Lee, Tang; University of British Columbia, Obstetrics & Gynaecology<br>Vilanculo, Faustino; Centro de Investigacao em Saude de Manhica<br>Munguambe, Khatia ; Centro de Investigacao em Saude de Manhica<br>Boene, Helena; Centro de Investigacao em Saude de Manhica<br>Ukah, UV; University of British Columbia, Obstetrics & Gynaecology<br>Vidler, Marianne; University of British Columbia, Obstetrics & Gynaecology<br>Magee, Laura; Kings College London, Obstetrics and Gynaecology<br>Sevene, Esperanca; Centro de Investigacao em Saude de Manhica; Universidade Eduardo Mondlane<br>von Dadelszen, Peter ; Kings College London, Obstetrics and Gynaecology<br>Firoz, Tabassum; Yale New Haven Health System
**Primary Subject Heading**:	Global health
Secondary Subject Heading:	Epidemiology, Global health, Obstetrics and gynaecology
Keywords:	Maternal health, Global health, Spatial epidemiology, Geostatistics,, Health geography

SCHOLARONE™
Manuscripts

Title: The place-specific factors associated with adverse maternal, and perinatal outcomes in southern Mozambique: A retrospective cohort study

Corresponding author: Makanga, Prestige Tatenda
Postal address: Midlands State University Faculty of Science and Technology, Surveying and Geomatics
P. Bag 9055
Senga Township
Gweru, Midlands, ZW
E-mail: makangap@staff.msu.ac.zw
Telephone: +263 54 60409 / +263 54 60450/ +263 54 60667

**Co-Author**	**Institution**
Makanga, Prestige Tatenda	Midlands State University Faculty of Science and Technology, Surveying and Geomatics<br>Gweru, Midlands, ZW
Sacoor, Charfudin	Centro de Investigacao em Saude de Manhica, Department of Demography<br>Rua 12, Bairro Cambeve<br>Manhica, MZ 1929<br>+25821810002
Schuurman, Nadine	Simon Fraser Univ, Geography<br>Vancouver, BC, CAN
Lee, Tang	University of British Columbia, Obstetrics & Gynaecology<br>Vancouver, BC, CAN
Vilanculo, Faustino Carlos	Centro de Investigacao em Saude de Manhica, Manhica, Maputo, MZ
Munguambe, Khatia	Centro de Investigacao em Saude de Manhica, Manhica, Maputo, MZ
Boene, Helena	Centro de Investigacao em Saude de Manhica, Manhica, Maputo, MZ
Ukah, Ugochinyere Vivian	University of British Columbia, Obstetrics & Gynaecology<br>Vancouver, BC, CAN

For peer review only

Vidler, Marianne	University of British Columbia, Obstetrics & Gynaecology<br>Vancouver, BC, CAN
Magee, Laura A	Kings College London, Obstetrics and Gynaecology<br>London, London, UK
Sevene, Esperanca	Centro de Investigacao em Saude de Manhica, Manhica, Maputo, MZ<br>Universidade Eduardo Mondlane, Maputo, MZ
von Dadelszen, Peter	King's College London School of Medical Education<br>London, London, UK
Firoz, Tabassum	Yale New Haven Health<br>New Haven, CT, USA
*CLIP working group	Centro de Investigacao em Saude de Manhica<br>Manhica, Maputo, MZ<br>University of British Columbia, Vancouver, BC, CAN

Keywords: Maternal health, global health, health geography, geostatistics, spatial epidemiology

Word count: 4458 words

For peer review only

The place-specific factors associated with adverse maternal, and perinatal outcomes in southern Mozambique: A retrospective cohort study

Authors: *Makanga, P.T., Sacoor, C., Schuurman, N., Lee, T., Vilanculo, F., Munguambe, K., Boene, H., Ukah, U.V., Vidler, M., Magee, L.A., Sevene, E., von Dadelszen, P., Firoz, T: On behalf of the CLIP Working Group**

Abstract

Objectives: To identify and measure the place specific determinants that are associated with adverse maternal and perinatal outcomes in the southern region of Mozambique.

Design: Retrospective cohort study. Choice of variables informed by literature and Delphi consensus.

Setting:

Study conducted during the baseline phase of a community level intervention for preeclampsia that was led by community health worker

Participants: A household census identified 50,493 households that were home to 80,483 women of reproductive age (age 12 - 49). Of these women, 14,617 had been pregnant in the 12 months prior to the census, of which 9,172 (61.6%) had completed their pregnancies.

Primary and secondary outcome measures: A combined fetal, maternal and neonatal outcome was calculated for all women with completed pregnancies

Results: A total of six variables were statistically significant ($p \leq 0.05$) in explaining the combined outcome. These included; geographic isolation, flood proneness, access to an improved latrine, average age of reproductive age woman, family support and fertility rates. The performance of the OLS model was an adjusted R2 = 0.69. Three of the variables (isolation, latrine score and family support) showed significant geographic variability in their effect on rates of adverse outcome. Accounting for this modest non-stationary effect through Geographically Weighted Regression increased the adjusted R2 to 0.71.

Conclusions: The community exploration was successful in identifying context-specific determinants of maternal health. The results highlight the need for designing targeted interventions that address the place specific social determinants of maternal health in the study area. The geographic process of identifying and measuring these determinants, therefore, has implications for multisectoral collaboration.

Trial registration: (CLIP, NCT01911494)

Summary

Strengths

For peer review only

- This study's methods draw from the value to mixing methods and validates the importance of variables through firstly seeking community perspectives, followed by expert opinion through a Delphi consensus and lastly a rigorous and thorough process of selecting the most important statistical variables.

- Small area analyses that are implemented to granuralize the data and reveal patterns that are normally masked in national averages to elucidate more locally specified linkages between health outcomes and associated determinants.

Limitation

- The key statistical association that have been uncovered in this research, particularly those from the geographically weighted regression, are specific to this region of Mozambique and may need to be validated before generalizing to other settings. Some of the variables that are significantly related with the combined outcome (e.g. latrine score) may be a symptom of a deeper socio-economic problem that this research did not have data and evidence to elucidate.

All maps were created using data generated from the study. Methods of creating the neighborhood boundaries have been published in a separate manuscript.

Introduction

Improving maternal health has been a global health priority for the past four decades. As global attention shifts from surviving pregnancy and childbirth to ensuring that women thrive throughout their life course (1,2), much remains to be done to lessen the harmful consequences of pregnancy and childbirth. Maternal deaths are only a small portion of the global maternal burden of ill-health; it is estimated that for each death; nearly 20 additional women suffer from life-long disabilities as a result of severe pregnancy-related morbidity (3,4). In line with that, Sustainable Development Goal 3 not only aims to improve maternal care but also focuses on lifelong well-being for women and children, particularly in low and middle income countries where the maternal mortality rate is up to 14 times more prevalent than in high income countries (5).

The known risk factors leading to maternal and perinatal death and morbidity exist both at the level of the individual women and at the level of her community. Some of these factors, although experienced at the individual level, are a function (at least in part) of the broader socio-cultural environment, making it hard to separate the two. Individual-level factors include maternal age (6), level of education for women and their partners (7), contraceptive use (8), birth spacing (9), marital status, social standing, self-esteem, and psycho-social stress (10). Environmental and community-level factors that elevate the risk for adverse pregnancy outcomes include physical isolation from health facilities (11), living in war zones (12,13), natural disasters (14), and religion (13,15) among others.

For peer review only

MMR estimates in Mozambique vary from approximately 250 to 408 deaths per 100,000 live births(16–18), and is among the top 20 countries with the highest MMRs globally. There has been approximately 50% reduction in MMR from 541/100,000 live births since 1990, largely due to falling rates of maternal death resulting from direct (as opposed to indirect) obstetric causes (16). In contrast, there has been a relative increase in maternal deaths from indirect causes (such as HIV, malaria) over the same period, as fewer obstetric interventions address these conditions.

Previous studies conducted in Mozambique have documented some of the determinants of maternal health in the country. In southern Mozambique, women who suffered severe maternal morbidities reported that lack of money for transportation and poor road infrastructure and long distances to health facilities caused delays in reaching health facilities, when they sought emergency pregnancy-related care (17,19). Flooding can isolate communities from obstetric care for months at a time (20). A cultural acceptance of the male decision maker's absence from matters concerning the woman's pregnancy may contribute to increasing the vulnerability of pregnant women (21) who may not be empowered to make decisions concerning their pregnancies (19). Prevailing misconceptions around the causes of pregnancy-related illness (22) are likely to influence women's choices to to access care .

Emerging global maternal health strategies acknowledge the broad nature of the associated determinants of health, and call for multi-sectoral approaches to complement health system enablers for improving maternal, fetal and child health along the continuum of care (1,23). These strategies mirror the aim of the Sustainable Development Goals (SDGs) to "draw on contributions from indigenous peoples, civil society, the private sector and other stakeholders, in line with national circumstances, policies and priorities" (2). Further to that, the SDGs call for greater measurement of disaggregate subnational trends in life-course health outcomes and associated determinants (2). This drive to understand the granular population health trends and associated determinants will likely help to better elucidate the place-specific nature of these associations with maternal and perinatal health outcomes.

The aim of this study was to identify and measure the community specific determinants that are associated with maternal and perinatal ill-health in the southern region of Mozambique. In line with the recommendations from the SDGs, the study sought to gain a local understanding of these determinants and how their associations with adverse maternal outcomes varied geographically.

Methods

Study setting

The study was conducted as part of the feasibility study for the Community-Level Interventions in Pre-Eclampsia Trial (CLIP, NCT01911494) in 2014 in Mozambique. CLIP was a community-based cluster randomized controlled trial, aimed at reducing all-cause maternal and perinatal

For peer review only

mortality and morbidity in the study region. CLIP was led by the University of British Columbia (UBC), in partnership with the Centro de Investigação em Saúde de Manhiça (CISM) in Mozambique. The feasibility study for CLIP was conducted in 36 administrative regions termed localities within two provinces in the southern part of the country. Ethics approval for the study was obtained by the research and ethics boards at UBC, Simon Fraser University and CISM.

Study Design

There were four core aspects of this project that are summarized in Figure 1: 1) gathering data on community perspectives of the determinants of maternal health, 2) prioritizing variables through a Delphi consensus, 3) collecting primary empirical data on the variables, and 4) conducting spatial and statistical analyses to explore the association of these variables with adverse maternal outcomes.

Community perspectives on the determinants of maternal health

Ten focus groups discussions (FGDs) were conducted in four of the 12 clusters in the CLIP study area: Messano, 3 de Fevereiro, Ilha Josina + Calanga and Chongoene. These FGDs involved pregnant women, women of reproductive age, matrons (local birth attendants), male partners, community leaders and community-based health workers. Using purposive sampling combined with snowball sampling techniques, participants for the FGDs were recruited with the assistance of community gatekeepers. The FGDs covered topics regarding the socio-cultural, environmental and economic factors thought to be related to adverse maternal events.

Semi-structured interviews were conducted with the chiefs in all 12 administrative posts in the study region to better understand the historical context (e.g. civil wars, natural disasters, foreign aid, microfinance etc.) of the communities and how these could impact maternal health.

The FGDs and interviews were conducted in a local language (Changana) following a guide of open-ended questions reflecting the topics, were audio-recorded, transcribed and translated verbatim into Portuguese before a final translation into English.

The full details concerning data collection, coding of the data and thematic analysis have been previously published (24).

Prioritizing variables

A Delphi consensus meeting by teleconference was conducted to prioritize the variables for statistical analysis. The Delphi technique helps with "achieving convergence of opinion concerning real-world knowledge solicited from experts within certain topic areas" (25). The panel of 17 experts had a range of relevant backgrounds, including obstetrics (N=2), epidemiology (N=2), demography (N=2), health geography (N=1), environmental health (N=1),

For peer review only

spatial statistics (N=1), health equity (N=1), health systems research (N=1), medical anthropology (N=4), and mobile health (N=2). A structured questionnaire that had been designed based on an extensive literature review was used as a guide for the delphi process during the teleconference. The same questionnaire was sent to members of the Delphi group that could not make the call. Consensus was reached after the first round as many of the variables tabled before the experts were backed by literature.

Participants and data sources

The context-specific variables identified for consideration of their association with a combined maternal and perinatal adverse outcome were collected through a household census conducted as part of the CLIP feasibility study (26). The census included information on all women who had been pregnant in the 12 months prior to the census, as well as women of reproductive age who had died. Data collected included individual-level variables (e.g. age, education, and pregnancy history), as well as community characteristics (e.g. availability of the household head and community support initiatives). All reports of maternal, fetal, or perinatal deaths were followed-up with verbal autopsy (27) to classify the cause.

The census identified 50,493 households that were home to 80,483 women of reproductive age (age 12 - 49). Of these women, 14,617 had been pregnant in the 12 months prior to the census, of which 9,172 (61.6%) had completed their pregnancies. For the mother, there were 18 deaths (204.6 MMR) of which the verbal autopsy identified that 38% were from direct, and 62% from indirect causes. For the baby, there were 288 (3.0%) miscarriages, 466 (4.9%) stillbirths, and 8,796 (92.1%) livebirths of which there were 117 neonatal deaths. A full description of the health and socio-demographic profile of the women of reproductive age in the study area has been published (26).

In addition, we collected five geographical variables using geographical information systems. There were three travel times to: (1) primary health facilities, (2) secondary health facilities, and (3) tertiary health facilities, using mixed transport modes for public transport and ambulances. Walking times to the nearest main road (4) were calculated to measure the degree to which communities were geographically isolated. Finally, an indicator for flood proneness (5) was designed based on flood and precipitation records from the previous year (28). These variables and other community-level estimates for the variables captured in the census were calculated for each locality in the study area as described in Table 1. Both the census and geographical data were aggregated into community-level averages at the locality level for each of the chosen variables, as ethical approval did not allow to analyse the location data at the level of the individual woman.

For peer review only

Statistical methods and variables

The primary outcome for this study was a combined maternal and perinatal outcome which included maternal, fetal and neonatal deaths. The denominator was the totalnumber of livebirths. A composite outcome was chosen as powering the study for maternal death alone would have required a prohibitively large sample size. There is clinical plausibility in combining the three outcomes as both fetal and early neonatal outcomes are related to the woman's condition during the antenatal and intrapartum periods, while her environment and socio-cultural circumstances have an impact on late neonatal outcomes (29,30). Furthermore, a significant proportion of miscarriages are related to placental dysfunction, as are stillbirths and neonatal deaths (31,32).

The spatial statistics module within ArcGIS software (33) was used for exploratory regression to further prioritize variables and to create the global ordinary least squares (OLS) regression model. The exploratory regression exercise evaluated different combinations of our explanatory variables for their fit for an OLS model, and how these explained trends in our outcome variable. This method implements the exploration by screening variables in a forward stepwise sequence, exploring how different combinations of variables fit and perform in the regression model. Using criteria that assessed *p* values significance, multi-collinearity measured by the variance inflation factor (VIF), normality of residuals, and clustering of residuals in space (Table 2), we selected the variables that best explained the outcome and met the criteria of a well specified regression model and explored these through a more rigorous OLS modelling exercise.

For peer review only

Table 1: Community level variables potentially associated with the rates of adverse maternal outcomes

Community level variable	Description *(Variables calculated for reproductive age women with completed pregnancies)*
**Census Variables**	
1. Age of reproductive age woman	Average age of reproductive age woman
2. Household head's education	Average number of years that household heads (man or woman) have spent in school *(No schooling = 0; At least Primary = 7; At least secondary = 12; At least a degree = 16; Graduate = 18, post grad = 20)*
3. Household head's availability	Percentage of households where the household head lives in the house
4. Water source score	Percentage of households that have an improved water source
5. Latrine score	Percentage of households that have an improved latrine
6. Private transportation score	Percentage of reproductive age women who live in a house where someone owns a private car
7. Reproductive age women's education	Average number of years that reproductive age women have spent in school *(No Schooling = 0, grade 5 = 5, grade 7 = 7, grade 10 = 10, grade 12 = 12, Bachelors = 16, Graduate = 18, post grad = 20)*
8. Fertility rate	Average number of children born to each woman in the community that had a completed pregnancy
9. Reproductive age women's marital status score	Percentage of reproductive age women in a marital union (monogamous or polygamous) relative to total with completed pregnancies
10. Reproductive age women's unemployment rate	Proportion of reproductive age women that do not work compared with total reproductive age women with a completed pregnancy
11. Family support	Percentage of reproductive age women that would receive financial, transport and emotional help from family or neighbors for a pregnancy related need
12. Community group support	Percentage of reproductive age women that would receive financial, transport and emotional help from a community-based group for a pregnancy related need
13. Financial autonomy in pregnancy	Percentage households where the reproductive age woman is empowered to make financial decisions concerning her pregnancy
**Geospatial variables**	
14. Access to primary health facilities	Average travel time to the nearest primary health facility, using public transport
15. Access to secondary health	Average travel time to the nearest secondary health facility, using a mix of public transport and an

For peer review only

facilities	ambulance
16. Access to tertiary health facilities	Average travel time to the nearest tertiary health facility, using a mix of public transport and an ambulance
17. Isolation	Average walking time to the nearest main road
18. Flood proneness	The difference between the road quality indicator (RoQI) score on a typical day in the dry season and on the worst day in the wet season. RoQI scores range between 0 and 100 and are a function of the quality of roads in a community

For peer review only

Table 2: Criteria for variable selection prior to regression modeling

Criteria	Description	Threshold
Coefficient p-value	The confidence interval required for p values of coefficients	< 0.05
Variance inflation factor	Measures redundancy of multi-collinearity between the explanatory variables	< 7.5
Jarque Bera p-value	Measures whether the model residuals are normally distributed.	> 0.1
Spatial autocorrelation p-value	Check for spatial clustering of model residuals	> 0.1

Global regression model

The performance of the OLS models chosen from the exploratory regression were assessed based on the magnitude of the adjusted R^2 values. In addition, we checked for significance of p values for the model coefficients. Multi-collinearity between different variables in a model was checked using the lower VIF threshold of five. The Koenker statistic ($p < 0.01$) was used to check if the relationships being modelled were consistent (either due to non-stationarity or heteroskadisticity), while the Wald statistic was used to assess overall model significance. The Jarque Bera test ($p < 0.01$) was used to check if model predictions were biased (i.e. if the model residuals were normally distributed). The model that performed best and met these criteria was selected for further analysis to create a locally-specified model.

Local regression model

The Geographically Weighted Regression (GWR) technique was used to develop a second model, which extended the output from OLS, to explore spatial non-stationarity of effect of the variables. This allowed for the new model to account for spatial structure in estimating local rather than global model parameters (34,35). We foresee this to be an important step to creating interventions that are locally specific, and an important part of more precisely targeting interventions. As part of the modelling process, the spatial weights based on the geographic proximity of observation are applied to give more weight to values that were closer together. GWR4 software (36) was used for this part of the project. The geographic variability test was conducted to assess if there was significant non-stationarity in the coefficients after applying GWR. This test compares the geographically varying parameter with those in the fixed global model, where a negative difference (abbreviated “DIFF OF CRITERION” in GWR4),

For peer review only

indicates significant variation in parameter estimates across space (36). We also assessed the performance of the GWR model using the newly calculated values of the adjusted R^2.

Patient and Public Involvement

Previous research (24) that elicited community perceptions of risk factors related to adverse maternal outcomes from women, their male counterparts and community leaders in the study area informed the choice for some of the variables. This study used focus group discussions and In depth interviews and has been published through other avenues. The same results have also been disseminated through an outreach workshop to the ministry of health in Mozambique. Further research that has received recent funding will communicate the identified risk factors through the use of a mobile App

Results

Community perspectives and choice of variables

The full list of community-level variables that were considered is presented in Table 1. These variables, gathered from the results of focus group discussions, semi-structured interviews, and the Delphi consensus, represent the local perspectives, expert views and *a priori* study knowledge. The community perspectives that informed some of our choice variables are reported in full separately (19,24).

Statistical analysis

Descriptive statistics

The geographic pattern for the rates of the combined adverse maternal and perinatal outcome is shown in Figure 2; where the clusters of Ilha Josina + Calanga, Mazivila, and Chissano had the localities with the highest rates of the combined outcome.

For women with completed pregnancies, community-level scores for census and geographical variables are summarized in Table 3. The average age of women with complete pregnancies was 26. Ninety-one percent of households reported that the head of household lived in the household. There was large variability in the percentage of households with an improved water source, with the lowest locality level score being 11.9% while the highest was 99%. The number of households with an improved toilet facility was very low, with the best locality level score for this variable being 31.6%. An average of 5.6% of all households reported owning a private vehicle. Most women reported being in a marital union (70.9%). The reported rates of

For peer review only

unemployment were surprisingly low (mean 11%), given how this was perceived as an important risk factors in the focus group discussions. The proportion of women who indicated that they would receive either financial, transport or emotional help from family or a neighbour in the event of a pregnancy-related need was 85.6%, while only 3.6% would receive the same from community groups. Twenty-two percent of households indicated that the woman was empowered to make financial decisions concerning her pregnancy.

The average travel time to the primary health facilities was 0.6 hours using public transport. For women who were referred to secondary facilities, this was calculated to take an average of 1.2 hours, assuming that they used public transport to primary facilities and an ambulance to secondary facilities. For tertiary facilities, it was calculated to take and average of 2.1 hours using the same combination of transport modes. The most isolated communities required women to walk 2.0 hours to the nearest main road, while the closest were less than 6 minutes (0.1 hours) away. The ease of travel through communities reduced by an average of 6.8% because of flooding and precipitation during the 12 months prior to the household census.

For peer review only

Table 3: Summary statistics

Community level variable	Min	Max	Mean	Std Dev
**Census Variables**				
1. Average age of Reproductive age women (Years)	24.30	28.00	26.36	0.90
2. Household head's education (Years)	3.60	7.30	5.47	0.88
3. Household head's availability (proportion)	0.80	1.00	0.91	0.07
4. Water source score (%)	11.90	99.00	54.68	25.97
5. Latrine score (%)	0.00	31.60	15.48	8.35
6. Private transportation score (%)	0.00	12.30	5.57	3.05
7. Reproductive age women's education (Years)	3.80	7.20	5.29	0.95
8. Fertility rate (No. per woman)	2.40	3.80	2.89	0.29
9. Reproductive age women's marital status score (%)	52.40	88.90	70.85	8.47
10. Reproductive age women's unemployment rate (proportion)	0.00	0.40	0.11	0.10
11. Family support (%)	61.30	100.00	85.61	10.46
12. Community support (%)	0.00	17.30	3.59	3.86
13. Financial autonomy in pregnancy (%)	0.00	45.90	22.15	8.43
**Geospatial variables**				
14. Access to primary health facilities (Hrs)	0.18	1.75	0.61	0.36
15. Access to secondary health facilities (Hrs)	0.29	2.80	1.20	0.54
16. Access to tertiary health facilities (Hrs)	0.97	4.32	2.08	0.74
17. Isolation (travel time to nearest main road in hours)	0.10	2.01	0.54	0.42
18. Flood proneness (%)	5.61	8.27	6.75	0.67
**Rate of adverse outcomes per live birth (Maternal + Neonatal + Miscarriages + Stillbirths)**	**0.01**	**0.21**	**0.10**	**0.05**

Global model

Through exploratory regression, we identified six variables that met pre-specified criteria for inclusion in the model (Supplementary file 1). The resulting OLS model is illustrated in Table 4. The adjusted R^2 was 0.69 (Supplementary file 2). A full record of the diagnosis for the OLS is provided as supplementary material. The graduated colour classification maps describing the magnitude of the variables across the study area is presented in figure 3.

For peer review only

Table 4: OLS model

Variable	Coefficient	Std Error	t-Statistic	VIF
Intercept	-0.194483	0.19802	-0.98214	--------
Isolation (Hrs)	0.033353*	0.01464	2.27805	2.06598
Flood proneness (%)	0.023936**	0.00754	3.17587	1.35311
Latrine score (%)	-0.003094***	0.00072	-4.28457	1.92347
Family support (%)	-0.001274*	0.00051	-2.48055	1.52648
Age of reproductive age woman (Years)	0.034653***	0.00826	4.19544	2.93335
Fertility rate (No. of children)	-0.222607***	0.02942	-7.56575	3.90853

* $p \leq 0.05$; ** $p \leq 0.01$; *** $p \leq 0.001$
Multiple $R^2 = 0.75$ Adj $R^2 = 0.69$

The OLS model shows that as the degree of isolation increases, there is an effect of increasing rates of adverse outcomes ($p \leq 0.05$). There is significantly more isolation in the western region of the study area, particularly for the Magude, Ilha Josina, and Calanga clusters, making women in these areas more vulnerable to the effect of isolation.

Communities that were shown to have more fragile and flood prone road infrastructure during the year prior to data collection were also shown to have elevated rates of the adverse outcome ($p \leq 0.01$). Regions in the Ilha Josina + Calanga, Mazivila and Chaimite clusters were the most affected by flooding and precipitation.

Higher rates of availability of an improved latrine are associated with lower rates of the adverse outcome ($p \leq 0.001$). Regions in the Ilha Josina + Calanga, Magude and Chaimite clusters had the lowest rates of improved latrines, while 3 de Fevereiro and Chongoene has the highest rates.

Family support emerged as an important characteristic in reducing the rates of adverse outcomes ($p \leq 0.05$). Although the levels of family support are relatively high for all localities (mean 85.6%) there is a north-south divide between the communities that have higher and lower rates of family support with Maluana + Maciana, Ilha Josina + Calanga and 3 de Fevereiro having the highest rates while Mazivila, Chissano and the northern region of Magude have lower rates.

Average age of women with a completed pregnancy was positively associated with rates of the adverse outcome ($p \leq 0.001$), while fertility rates are negatively associated with the rates of

For peer review only

adverse outcomes (p ≤ 0.001).. A closer look at the age aggregated data for age and fertility rates (Figure 4) reveals a bi-directional trend in the both relationships with the combined outcome. Rates of the combined outcome are relatively higher for the younger ages of 12 years old and decrease until age 20, where they begin to rise until age 49. A similar pattern exists for fertility rates with women who have lowest fertility rates experiencing higher rates of adverse outcomes that decline until fertility rates of approximately 2.2 before rising again for higher fertility rates.

Local model

The GWR model indicated that the effect on the combined outcome was geographically non-stationary for 3 of the 6 variables (isolation, latrine score and family support) as indicated by the negative DIFF OF CRITERION values in Table 5 (Supplementary file 3). There was no non-stationarity for the effect of flood proneness, age of the woman and fertility rate. The performance of the local model improved modestly from the global model to an adjusted R^2 value of 0.7, explaining a further 2% of the variability in the outcome. A graduated color classification was used to describe the magnitude of the effect in each of the localities. The transcripts of the GWR results are available as supplementary material.

Table 5: Results of the geographic variability test

VARIABLE	DIFF OF CRITERION
Intercept	0.176197
Isolation	-1.224025
Flood proneness	0.530483
Latrine score	-0.156581
Family support	-2.551618
Age of reproductive age woman	0.073263
Fertility rate	0.190761

The general direction of the effect in the global model is preserved in the local model (Figure 5). Isolation has a general effect of increasing risk for adverse outcomes across the entire study area. However, the effect is more pronounced by a factor of approximately 53% in the western region of Magude compared to the east in Chongoene. A similar pattern is observed for the effect of flood proneness on the combined outcome, with the western regions of the study area being at greater risk, though the magnitude of the differential effect is much smaller (8%). This

For peer review only

is also even though the same areas in the west had lower level of proneness to floods (Figure 3).

Proportion of household with an improved latrine was associated with decreasing the adverse outcomes for all regions under study, though this effect is greatest in magnitude for the eastern region of the study area by a factor of about 12%. Family support has a greater effect of reducing the rates of adverse outcomes in the eastern regions by a factor of about 45%. Average woman's age had an effect of increasing the outcome by up to 15% more in the west than the east. The variability in the association of fertility rates to the rates of adverse outcomes was also non-stationary with the highest effect 5% more than the lowest.

Discussion

This study has explored the place-specific factors that are associated with rates of adverse maternal and perinatal outcomes. Information gathered through focus group discussions and semi-structured interviews enabled us to measure the context specific determinants that were thought to be related to adverse maternal and perinatal outcomes. Some of the variables from the FGDs and interviews were indeed significantly associated with the rates of our combined outcome and include family support, geographic isolation and access to an improved latrine facility. Other noteworthy variables include flood proneness, average age of reproductive age women with completed pregnancy and fertility rates. The effect and the magnitude of the effect of these determinants of the outcome varied between communities though the direction of the effect was largely constant.

Fertility rates were the only variable where the direction of the effect is contrary to common expectation. However, fertility rate was the most significant variable both in the global model ($p \leq 0.001$) and as a single variable in the exploratory regression, so the observed pattern is unlikely a result of effect modification. Instead it is possible that there are other pervasive factors are at play. The graph for fertility rates plotted against the rates of adverse outcomes (Figure 4) indicated that adolescent women with completed pregnancies, also have lower fertility rates but tend have high rates of the adverse outcome (26,37). This trend could have possibly skewed the direction of the effect of this variable. This pattern would be possible as the most completed pregnancies (61%) were from women with fertility rates less than 2.2; therefore, community-level averages would naturally carry more weight from these women's records.

This is the first time that the place-specific socio-cultural and environmental factors related to adverse maternal outcomes have been explored in this region of Mozambique. Similar methods have been used in the USA (38). However, most of these studies emphasize health systems-related variables and how they relate to adverse maternal outcomes. This project's approach of going into communities to meet with local stakeholders is aligned with upcoming strategies within the SDGs for improving maternal health (United Nations, 2015). Core to these new global health strategies is an emphasis on multi-sectoral interventions that broadly consult multiple

For peer review only

local stakeholders to understand the context specific factors that may be related to population level health trends. The value of geographical techniques to these new strategies is demonstrated in two ways in this project.

First, we used GIS to design new indicators for some of the context specific variables perceived to be related to adverse maternal outcomes. Our measure of isolation for example (distance from highway) was designed based on local knowledge from the FGDs and interviews and implemented in a GIS. Other GIS variables on access to care were generated based on an understanding from the focus group discussions and relevant literature that most women would either walk or use public transport to primary facilities and be driven to higher level facilities (39–41) .

Second, the use of geographically-explicit techniques, such as GWR, enhances the ability to elucidate the spatial structure of the determinants of health and is in line with a drive within global health (expressed through the SDGs) to measure more granular subnational trends in health (2). Evidence from the local regression model developed for this project highlights that different determinants matter to different extents in different places. This understanding is valuable, particularly for designing and targeting population level interventions for improving maternal and child health because it allows for action in specific places to be informed by what is known to elevate risk the most. This is critical for achieving impact and improving health in a resource limited settings like Mozambique (42,43).

A key limitation for this study is that the results can only be applied at the population level and should not be used to predict outcomes at the individual woman level. This phenomenon is termed ecological fallacy (44) and is a key drawback for conducting population-level studies like this one. Furthermore, this project created a combined outcome for maternal, fetal and neonatal outcomes. The implication for this is that the results may not mirror the actual associations with any of the three outcomes in the combined outcome if considered separately. However, the observed patterns address more broadly, an approach to improving maternal health along the continuum of care that includes thinking about fetal, newborn and child survival together (5). Furthermore, the combined outcome better captures the true impact on families, as tragedies of death of mothers, fetuses and infants do not happen in isolation.

Conclusions

The geographic perspective contributes to new strategies for improving global maternal and perinatal health by providing the tools required to understand local contexts and associated determinants. It also helps to elucidate the geographic structure of associations between these determinants and adverse maternal and perinatal health outcomes. This is crucially important for targeting interventions and can help to operationalize some of the key strategies within the new SDG. While the patterns that characterize the findings of this project are specific to the region of southern Mozambique, and may not be transferable to other settings, this research design could certainly be translated to help with understanding the local factors that elevate

For peer review only

risk for adverse maternal and perinatal outcomes. It was outside the purview of this research to explore how this evidence could be translated into action on these community specific social determinants, and future work should address this knowledge gap.

Acknowledgements

A. contributor ship statement

PTM, CS, NS, PvD and TF made substantial contributions to conception and design of the project. FV, CS, KM, HB and ES led the acquisition of in country data. PTM, VUU, NS, TL, led the analysis and were involved together with all the authors in the interpretation of the results. PTM, LM, TF, NS and MV made substantive contributions to writing the first complete version of the article, and all the authors were involved in revising it critically for important intellectual content. All authors approve of the final version of the paper.

B. competing interests

NONE

C. funding

This work was part funded by Grand Challenges Canada- Stars in Global Health program (Grant 0197) and was conducted as part of the PRE-EMPT (Pre-eclampsia/Eclampsia, Monitoring, Prevention and Treatment) initiative supported by the Bill & Melinda Gates Foundation.

D. data sharing statement

All data that have been used in this research will be available upon request after the main papers from the CLIP trials have been published

CLIP Working Group: Eusébio Macete, Anifa Vala, Felizarda Amose, Rosa Pires, Zefanias Nhamirre, Marta Macamo, Rogério Chiaú, Analisa Matavele, Ariel Nhancolo, Silvestre Cutana, Ernesto Mandlate, Salésio Macuacua, Cassimo Bique, Sibone Mocumbi, Emília Gonçálves, Sónia Maculuve, Ana Ilda Biz, Dulce Mulungo, Orvalho Augusto, Paulo Filimone, Vivalde Nobela, Corsino Tchavana, Cláudio Nkumbula, Jeffrey Bone, Dustin Dunsmuir, Sharla K Drebit, Chirag Kariya, Mai-Lei Woo Kinshella, Jing Li, Mansun Lui, Beth A. Payne, Asif R Khowaja, Diane Sawchuck, Sumedha Sharma, Domena K. Tu, Ugochi V. Ukah,

For peer review only

References

1. United Nations. The Global strategy for women's, children's and adolescents' health (2016–2030) [Internet]. New York; 2015. Available from: http://www.who.int/life-course/partners/global-strategy/globalstrategyreport2016-2030-lowres.pdf?ua=1

2. United Nations. Transforming our world: the 2030 Agenda for Sustainable Development. See Relat Glob Initiat Transnatl Organ Crime"Organized Crime Cross-Cut Threat Sustain Dev January 2015. 2015;

3. Firoz T, Chou D, von Dadelszen P, Agrawal P, Vanderkruik R, Tunçalp O, et al. Measuring maternal health: focus on maternal morbidity. Bull World Health Organ. 2013;91(10):794–6.

4. Alvarez JL, Gil R, Hernández V, Gil A. Factors associated with maternal mortality in Sub-Saharan Africa: an ecological study. BMC Public Health. 2009;9(1):1–8.

5. World Health Organization. Strategies towards ending preventable maternal mortality (EPMM). 2015;

6. Olowonyo T, Oshin S, Obasanjo-Bello I. Registering in a health facility for delivery protects against maternal mortality in a developing country setting. J Obstet Gynaecol. 2005;25(7):638–41.

7. Wall LL. Dead Mothers and Injured Wives: The Social Context of Maternal Morbidity and Mortality among the Hausa of Northern Nigeria. Stud Fam Plann. 1998;29(4):341–59.

8. Ahmed S, Li Q, Liu L, Tsui AO. Maternal deaths averted by contraceptive use: an analysis of 172 countries. The Lancet. 2012;380(9837):111–25.

9. Conde-Agudelo A, Rosas-Bermúdez A, Kafury-Goeta AC. Effects of birth spacing on maternal health: a systematic review. Am J Obstet Gynecol. 2007;196(4):297–308.

10. Ronsmans C, Graham WJ. Maternal mortality: who, when, where, and why. The Lancet. 2006;368(9542):1189–200.

11. Grzybowski S, Stoll K, Kornelsen J. Distance matters: a population based study examining access to maternity services for rural women. BMC Health Serv Res. 2011;11(1):147.

12. Jambai A, MacCormack C. Maternal Health, War, and Religious Tradition: Authoritative Knowledge in Pujehun District, Sierra Leone. Med Anthropol Q. 1996;10(2):270–86.

13. O'Hare BAM, Southall DP. First do no harm: the impact of recent armed conflict on maternal and child health in Sub-Saharan Africa. JRSM. 2007 Dec 1;100(12):564–70.

For peer review only

14. Nour NN. Maternal health considerations during disaster relief. Rev Obstet Gynecol. 2011;4(1):22.

15. Evjen-Olsen B, Hinderaker SG, Lie RT, Bergsjø P, Gasheka P, Kvåle G. Risk factors for maternal death in the highlands of rural northern Tanzania: a case-control study. BMC Public Health. 2008;8(1):1.

16. Bailey PE, Keyes E, Moran AC, Singh K, Chavane L, Chilundo B. The triple threat of pregnancy, HIV infection and malaria: reported causes of maternal mortality in two nationwide health facility assessments in Mozambique, 2007 and 2012. BMC Pregnancy Childbirth. 2015;15(1):1.

17. David E, Machungo F, Zanconato G, Cavaliere E, Fiosse S, Sululu C, et al. Maternal near miss and maternal deaths in Mozambique: a cross-sectional, region-wide study of 635 consecutive cases assisted in health facilities of Maputo province. BMC Pregnancy Childbirth. 2014;14(1):1–8.

18. Ministerio da Saude (MISAU). INdEIaIII. MocËambique InqueÂ rito DemograÂ fico e de SauÂde 2011. Calverton, Maryland, USA:: MISAU, INE and ICFI., 2013. Calverton, Maryland, USA; 2011.

19. Munguambe K, Boene H, Vidler M, Bique C, Sawchuck D, Firoz T, et al. Barriers and facilitators to health care seeking behaviours in pregnancy in rural communities of southern Mozambique. Reprod Health. 2016;13(1):83–97.

20. Jamisse L, Songane F, Libombo A, Bique C, Faúndes A. Reducing maternal mortality in Mozambique: challenges, failures, successes and lessons learned. Int J Gynecol Obstet. 2004;85(2):203–12.

21. Audet CM, Chire YM, Vaz LM, Bechtel R, Carlson-Bremer D, Wester CW, et al. Barriers to male involvement in antenatal care in rural Mozambique. Qual Health Res. 2015;1049732315580302.

22. Boene H, Vidler M, Sacoor C, Nhama A, Nhacolo A, Bique C, et al. Community perceptions of pre-eclampsia and eclampsia in southern Mozambique. Reprod Health. 2016;13(1):27–37.

23. Kuruvilla S, Bustreo F, Kuo N, Mishra C, Taylor K, Fogstad H, et al. The Global strategy for women's, children's and adolescents' health (2016–2030): a roadmap based on evidence and country experience. Bull World Health Organ. 2016;94(5):398–400.

24. Firoz T, Vidler M, Makanga PT, Boene H, Chiaú R, Sevene E, et al. Community perspectives on the determinants of maternal health in rural southern Mozambique: a qualitative study. Reprod Health. 2016;13(2):123–31.

For peer review only

25. Hsu C-C, Sandford BA. The Delphi technique: making sense of consensus. Pract Assess Res Eval. 2007;12(10):1–8.

26. Sacoor C, Payne B, Augusto O, Vilanculo F, Nhacolo A, Vidler M, et al. Health and socio-demographic profile of women of reproductive age in rural communities of southern Mozambique. PLOS ONE. 2018 Feb 2;13(2):e0184249.

27. World Health Organization. Verbal autopsy standards: the 2012 WHO verbal autopsy instrument. Geneva WHO. 2012;

28. Global Flood Observatory. Dartmouth Flood Observatory - Space-based Measurement, Mapping, and Modeling of Surface Water For Research, Humanitarian, and Water Management Applications [Internet]. 2016 [cited 2016 Jan 17]. Available from: http://floodobservatory.colorado.edu/

29. Oza S, Lawn JE, Hogan DR, Mathers C, Cousens SN. Neonatal cause-of-death estimates for the early and late neonatal periods for 194 countries: 2000_2013. Bull World Health Organ. 2015;93:19–28.

30. Gardosi J, Madurasinghe V, Williams M, Malik A, Francis A. Maternal and fetal risk factors for stillbirth: population based study. BMJ [Internet]. 2013 Jan 24;346. Available from: http://www.bmj.com/content/346/bmj.f108.abstract

31. Roescher AM, Timmer A, Erwich JJHM, Bos AF. Placental Pathology, Perinatal Death, Neonatal Outcome, and Neurological Development: A Systematic Review. Kanellopoulos-Langevin C, editor. PLoS ONE. 2014;9(2):e89419.

32. Baschat AA, Cosmi E, Bilardo CM, Wolf H, Berg C, Rigano S, et al. Predictors of neonatal outcome in early-onset placental dysfunction. Obstet Gynecol. 2007;109(2):253–61.

33. ESRI. About ArcGIS. Envrionmental Systems Research Institute [Internet]. 2018 [cited 2018 Apr 5]; Available from: http://www.esri.com/arcgis/about-arcgis

34. Brunsdon C, Fotheringham AS, Charlton ME. Geographically weighted regression: a method for exploring spatial nonstationarity. Geogr Anal. 1996;28(4):281–98.

35. Fotheringham AS, Brunsdon C, Charlton M. Geographically weighted regression: the analysis of spatially varying relationships. John Wiley & Sons; 2003.

36. Nakaya T. GWR4 User Manual: Windows application for geographically weighted regression modelling [Internet]. 2014. Available from: https://geodacenter.asu.edu/drupal_files/gwr/GWR4manual.pdf

For peer review only

37. Pizzol D, Di Gennaro F, Boscardin C, Putoto G, Cuppini E, Pita G, et al. Teenage pregnancies in Mozambique: the experience of "Servicios Amigos dos Adolescentes" clinics in Beira. Afr J AIDS Res. 2018 Jan 2;17(1):32–6.

38. Shoff C, Yang T-C, Matthews S. What has geography got to do with it? Using GWR to explore place-specific associations with prenatal care utilization. GeoJournal. 2012 Jun 1;77(3):331–41.

39. Blanford JI, Kumar S, Luo W, MacEachren AM. It'sa long, long walk: accessibility to hospitals, maternity and integrated health centers in Niger. Int J Health Geogr. 2012;11(1):1–15.

40. Makanga PT, Schuurman N, Sacoor C, Boene HE, Vilanculo F, Vidler M, et al. Seasonal variation in geographical access to maternal health services in regions of southern Mozambique. Int J Health Geogr. 2017;16(1):1.

41. Munjanja SP, Magure T, Kandawasvika G. 11 Geographical Access, Transport and Referral Systems. Matern Perinat Health Dev Ctries. 2012;139.

42. Sartorius B, Sartorius K. Identifying and Targeting Mortality Disparities: A Framework for Sub-Saharan Africa Using Adult Mortality Data from South Africa. Plos One [Internet]. 2013 Aug;8(8). Available from: ://WOS:000323221500083

43. Friberg IK, Kinney MV, Lawn JE, Kerber KJ, Odubanjo MO, Bergh A-M, et al. Sub-Saharan Africa's mothers, newborns, and children: how many lives could be saved with targeted health interventions? PLoS Med. 2010;7(6):e1000295.

44. Dummer TJB. Health geography: supporting public health policy and planning. Can Med Assoc J. 2008 Apr 22;178(9):1177–80.

Figure Legend

Figure 1: Design overview

Figure 2: Geographic pattern for the rates of the combined adverse outcomes

Figure 3: Geographic patterns in values for the model variables

Figure 4: Age and fertility rates compared with adverse outcomes. The classic J shape graph for both

Figure 5: Geographic variation of beta coefficients

For peer review only

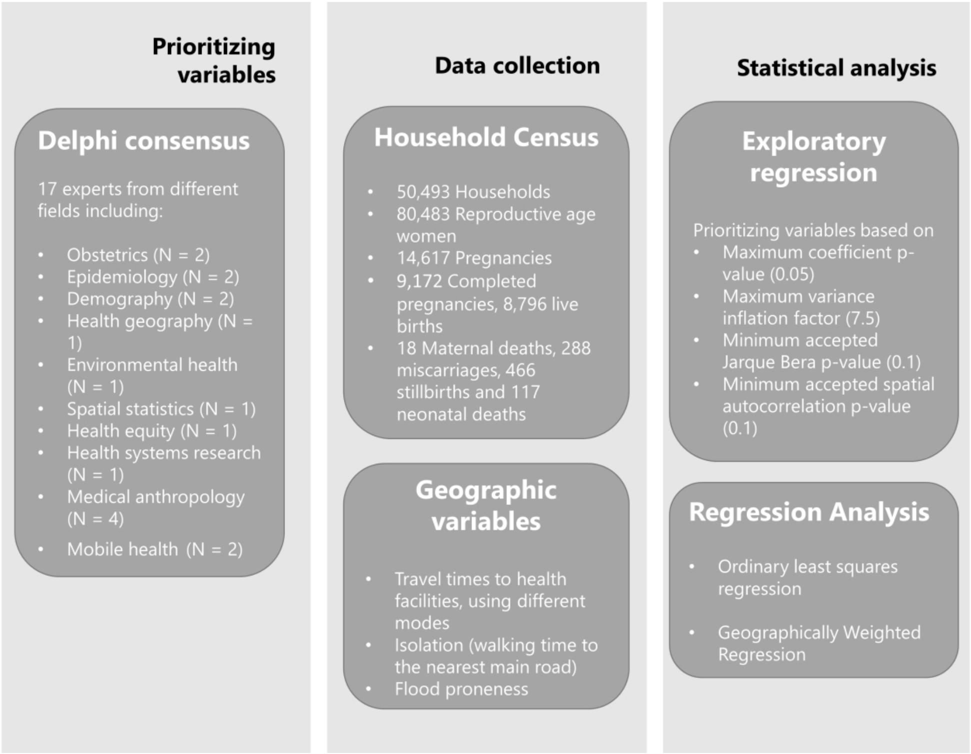

Figure 1: Design overview

313x235mm (300 x 300 DPI)

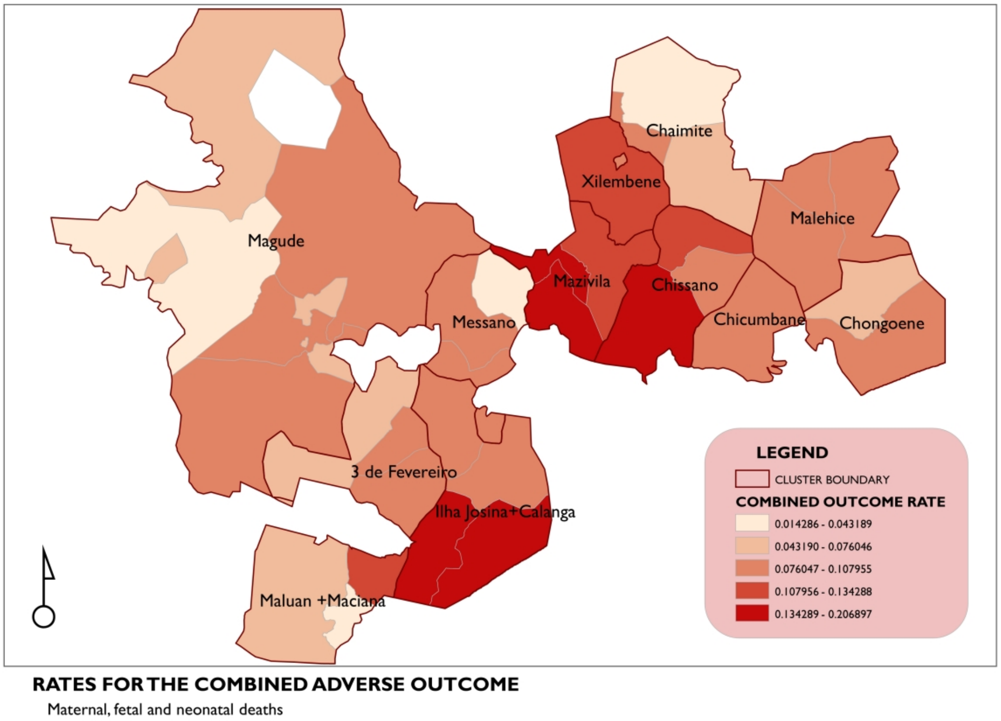

Figure 2: Geographic pattern for the rates of the combined adverse outcomes

312x227mm (300 x 300 DPI)

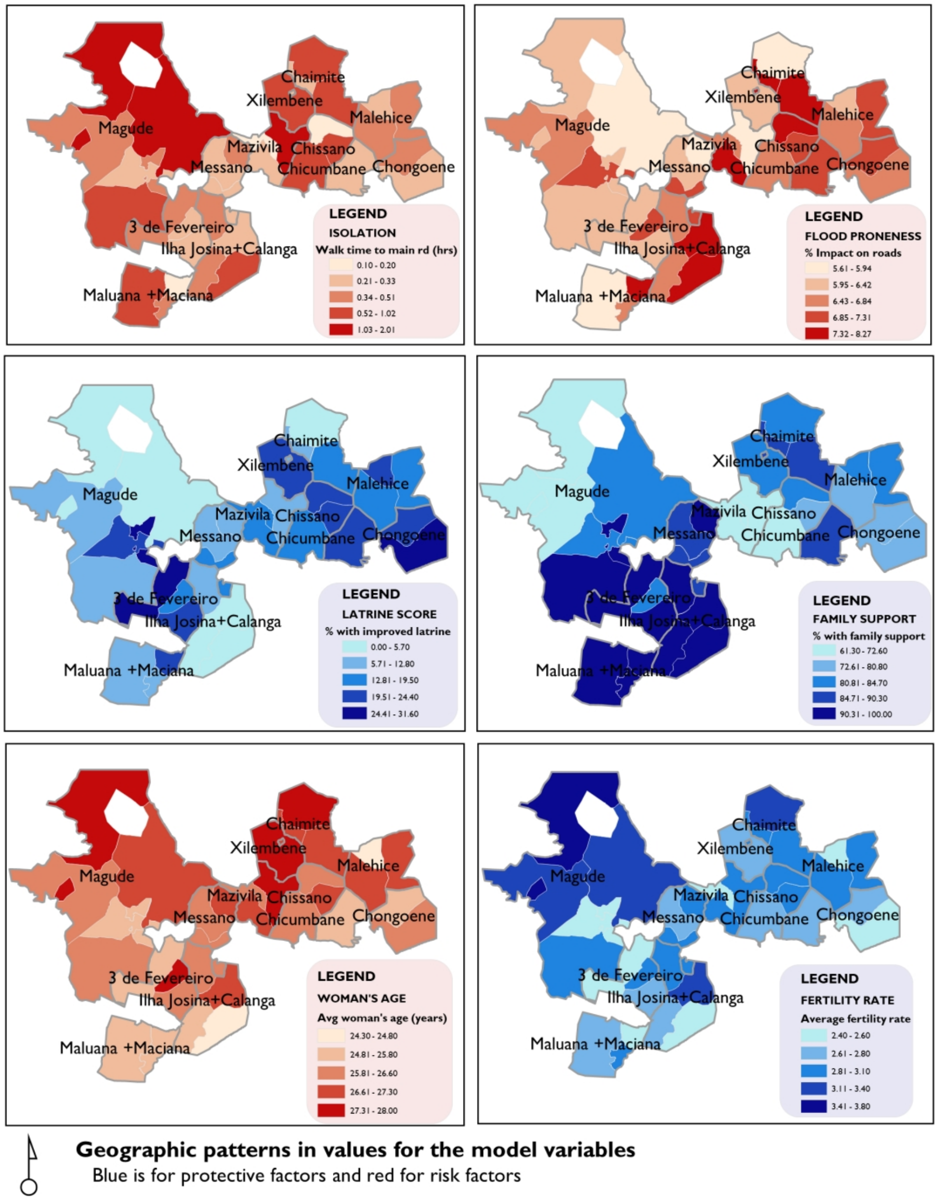

Figure 3: Geographic patterns in values for the model variables

169x217mm (300 x 300 DPI)

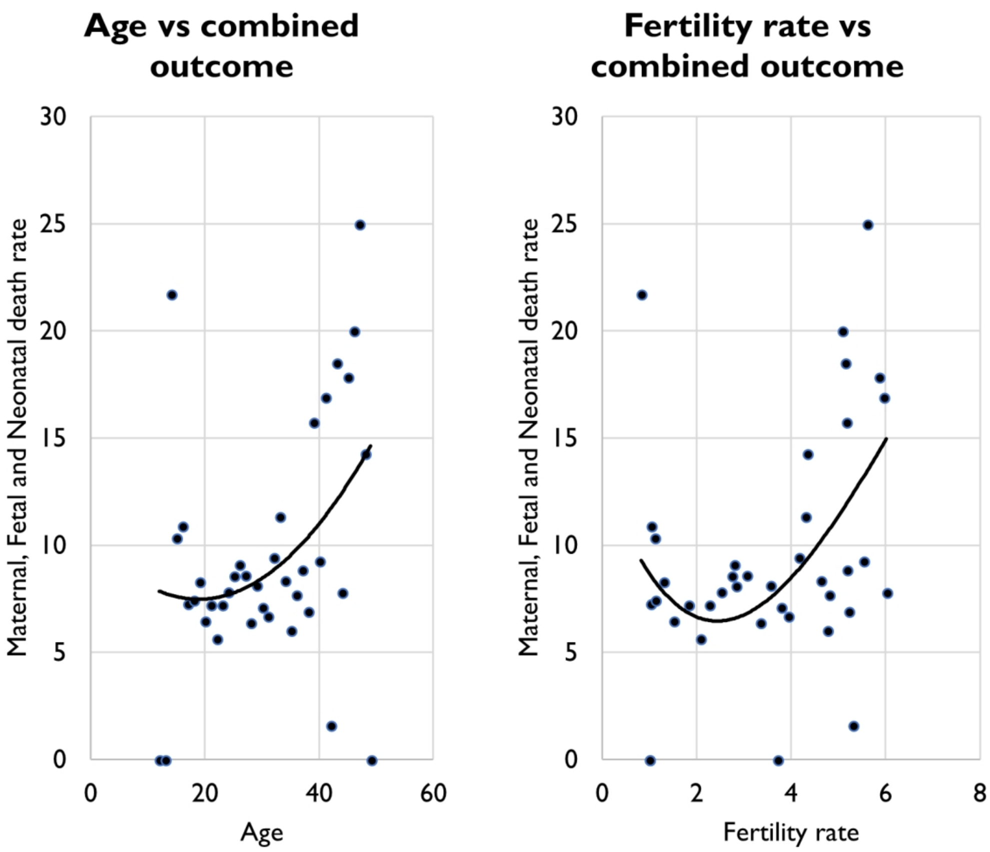

Figure 4: Age and fertility rates compared with adverse outcomes. The classic J shape graph for both

142x127mm (300 x 300 DPI)

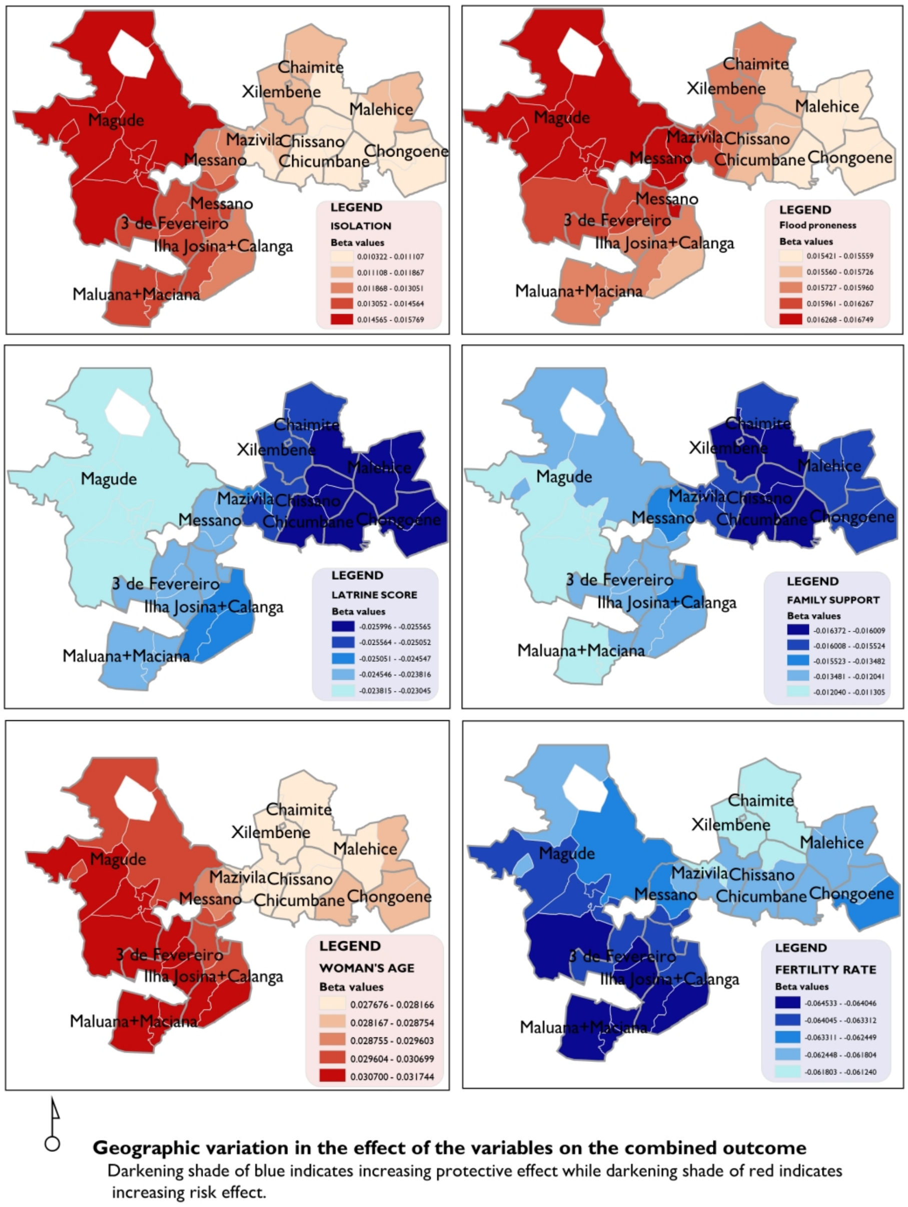

Figure 5: Geographic variation of beta coefficients

211x279mm (300 x 300 DPI)

```
Exploratory Regression.txt
VARIABLE DEFINITIONS:

Variable        Variable Name
FERTRATE        Fertility rate
FAMSUPP_RT      Family support
WRAAGE_RT       Average age of reproductive age woman
UNEMP_RT        Reproductive age women's unemployment rate
ROQI_IMPACT     Flood proneness
PRVTRANS_RT     Private transportation score
LATRINE_RT      Latrine score
WALK_MRD        Isolation
HHH_AVAIL       Household head’s availability
WATER_RT        Water source score
MARRIED_RT      Reproductive age women's marital status score
PUBDRV          Access to secondary health facilities
PUB             Access to primary health facilities
WRAEDUC         Reproductive age women’s education
PUBDRVDRV       Access to tertiary health facilities
HHHEDUC         Household head’s education
FINAUTPRE_RT    Financial autonomy in pregnancy
COMSUPP_RT      Community group support

******************************************************************************
Choose 1 of 18 Summary
     Highest Adjusted R-Squared Results
AdjR2    AICc   JB K(BP)  VIF   SA   Model
 0.16 -118.08 0.39  0.15 1.00 0.02  -FERTRATE***
 0.10 -115.69 0.18  0.76 1.00 0.04  +ROQI_IMPACT**
 0.08 -114.90 0.66  0.05 1.00 0.11  -UNEMP_RT**
       Passing Models
AdjR2 AICc JB K(BP) VIF SA   Model
******************************************************************************
Choose 2 of 18 Summary
            Highest Adjusted R-Squared Results
AdjR2    AICc   JB K(BP)  VIF   SA   Model
 0.35 -125.40 0.77  0.03 1.06 0.13  -UNEMP_RT***  -FERTRATE***
 0.29 -122.56 0.03  0.26 2.46 0.37  +WRAAGE_RT**  -FERTRATE***
 0.27 -121.23 0.91  0.35 1.05 0.01  -FAMSUPP_RT**  -FERTRATE***
       Passing Models
AdjR2 AICc JB K(BP) VIF SA   Model
******************************************************************************
Choose 3 of 18 Summary
                     Highest Adjusted R-Squared Results
AdjR2    AICc   JB K(BP)  VIF   SA   Model
 0.46 -130.66 0.26  0.64 3.68 0.71  -LATRINE_RT***  +WRAAGE_RT***  -FERTRATE***
 0.44 -129.33 0.56  0.55 1.50 0.08  -PRVTRANS_RT***  -FAMSUPP_RT***  -FERTRATE***
 0.44 -129.23 0.01  0.17 1.65 0.19  -LATRINE_RT***  -FAMSUPP_RT***  -FERTRATE***
       Passing Models
AdjR2 AICc JB K(BP) VIF SA   Model
******************************************************************************
Choose 4 of 18 Summary
                            Highest Adjusted R-Squared Results
AdjR2    AICc   JB K(BP)  VIF   SA   Model
 0.60 -139.23 0.53  0.04 3.69 0.64  -LATRINE_RT***  -FAMSUPP_RT***  +WRAAGE_RT***  -FERTRATE***
 0.52 -132.83 0.18  0.84 3.75 0.94  +ROQI_IMPACT**  -LATRINE_RT***  +WRAAGE_RT***  -FERTRATE***
 0.52 -132.54 0.46  0.82 1.65 0.15  +PUBDRV**  -PRVTRANS_RT***  -FAMSUPP_RT***  -FERTRATE***
                                                 Passing Models

AdjR2    AICc        JB       K(BP)    VIF      SA         Model
```


For peer review only

```
0.602028 -139.231246 0.531046 0.039391 3.694394 0.643045  -LATRINE_RT***  -FAMSUPP_RT***
+WRAAGE_RT***  -FERTRATE***
0.522176 -132.831155 0.175090 0.844169 3.754031 0.939831  +ROQI_IMPACT**  -LATRINE_RT***
+WRAAGE_RT***  -FERTRATE***
0.518237 -132.543818 0.458059 0.818336 1.650636 0.148576  +PUBDRV**  -PRVTRANS_RT***  -FAMSUPP_RT***
-FERTRATE***
0.515228 -132.325907 0.781122 0.499719 3.851258 0.772143  -LATRINE_RT**  -UNEMP_RT**  +WRAAGE_RT***
-FERTRATE***
0.513983 -132.236119 0.325629 0.334396 1.612012 0.103168  -WATER_RT**  -PRVTRANS_RT***
-FAMSUPP_RT***  -FERTRATE***
0.510264 -131.969343 0.166281 0.246145 1.982255 0.420674  -LATRINE_RT**  -PRVTRANS_RT**
-FAMSUPP_RT***  -FERTRATE***
******************************************************************************
Choose 5 of 18 Summary
                                    Highest Adjusted R-Squared Results

AdjR2    AICc   JB  K(BP)  VIF   SA   Model

 0.65 -141.61 0.54  0.02 3.77 0.67  +ROQI_IMPACT***  -LATRINE_RT***  -FAMSUPP_RT**  +WRAAGE_RT***
-FERTRATE***
 0.64 -140.61 0.63  0.35 3.91 0.98  +WALK_MRD***  +ROQI_IMPACT***  -LATRINE_RT***  +WRAAGE_RT***
-FERTRATE***
 0.63 -139.66 0.49  0.27 3.70 0.30  -LATRINE_RT***  -FAMSUPP_RT***  +WRAAGE_RT***  -FERTRATE***
+HHH_AVAIL*
                                                          Passing Models

AdjR2    AICc        JB       K(BP)    VIF      SA         Model

0.648492 -141.614920 0.536501 0.021780 3.774488 0.668519  +ROQI_IMPACT***  -LATRINE_RT***
-FAMSUPP_RT**  +WRAAGE_RT***  -FERTRATE***
0.638286 -140.613195 0.625085 0.345632 3.908376 0.976892  +WALK_MRD***  +ROQI_IMPACT***
-LATRINE_RT***  +WRAAGE_RT***  -FERTRATE***
0.615682 -138.491555 0.529587 0.076835 3.726831 0.442333  -LATRINE_RT***  +MARRIED_RT**
-FAMSUPP_RT***  +WRAAGE_RT***  -FERTRATE***
0.594995 -136.656610 0.367722 0.363750 3.541431 0.366122  +PUBDRV***  -PRVTRANS_RT***  -FAMSUPP_RT***
 +WRAAGE_RT**  -FERTRATE***
0.582505 -135.593509 0.556140 0.003931 3.357147 0.143597  -WATER_RT***  -PRVTRANS_RT***
-FAMSUPP_RT***  +WRAAGE_RT**  -FERTRATE***
0.568548 -134.442595 0.687727 0.113630 3.178226 0.277763  +WALK_MRD***  +ROQI_IMPACT***
+MARRIED_RT**  +WRAAGE_RT***  -FERTRATE***
0.564733 -134.134426 0.626979 0.464601 3.857918 0.255002  -LATRINE_RT**  -UNEMP_RT**  +WRAAGE_RT***
-FERTRATE***  +HHH_AVAIL**
0.563092 -134.002788 0.353655 0.096116 3.259366 0.415519  +PUBDRVDRV**  -PRVTRANS_RT***
-FAMSUPP_RT***  +WRAAGE_RT**  -FERTRATE***
0.552392 -133.155941 0.795824 0.613240 3.500445 0.197948  +PUBDRV***  +ROQI_IMPACT**  -UNEMP_RT**
+WRAAGE_RT***  -FERTRATE***
0.523659 -130.978388 0.575476 0.009866 3.319401 0.552068  -WATER_RT***  +MARRIED_RT**  -FAMSUPP_RT**
+WRAAGE_RT**  -FERTRATE***
******************************************************************************
Choose 6 of 18 Summary
                                    Highest Adjusted R-Squared Results

AdjR2    AICc   JB  K(BP)  VIF   SA   Model

 0.69 -144.18 0.38  0.55 3.91 0.92  +WALK_MRD**  +ROQI_IMPACT***  -LATRINE_RT***  -FAMSUPP_RT**
+WRAAGE_RT***  -FERTRATE***
 0.67 -142.16 0.70  0.10 3.78 0.21  +ROQI_IMPACT***  -LATRINE_RT***  -FAMSUPP_RT**  +WRAAGE_RT***
-FERTRATE***  +HHH_AVAIL**
```


For peer review only

Exploratory Regression.txt

```
 0.67 -141.32 0.49  0.49 4.07 0.94  +PUBDRV  +ROQI_IMPACT**  -LATRINE_RT***  -FAMSUPP_RT***
+WRAAGE_RT***  -FERTRATE***
                                                                    Passing Models

AdjR2    AICc        JB       K(BP)    VIF      SA         Model

0.692863 -144.175618 0.379263 0.549371 3.908531 0.923074  +WALK_MRD**  +ROQI_IMPACT***
-LATRINE_RT***  -FAMSUPP_RT**  +WRAAGE_RT***  -FERTRATE***
0.674630 -142.157235 0.695520 0.098209 3.778934 0.213202  +ROQI_IMPACT***  -LATRINE_RT***
-FAMSUPP_RT**  +WRAAGE_RT***  -FERTRATE***  +HHH_AVAIL**
0.635329 -138.166134 0.568820 0.898220 3.550214 0.205750  +PUBDRV***  +ROQI_IMPACT**  -PRVTRANS_RT***
 -FAMSUPP_RT***  +WRAAGE_RT***  -FERTRATE***
0.598989 -134.841346 0.651598 0.008895 3.478494 0.553360  -WATER_RT***  -PRVTRANS_RT**  +MARRIED_RT**
 -FAMSUPP_RT***  +WRAAGE_RT**  -FERTRATE***
0.597255 -134.690338 0.545878 0.001749 3.326038 0.523384  +WALK_MRD**  -PRVTRANS_RT***  +MARRIED_RT**
 -FAMSUPP_RT***  +WRAAGE_RT**  -FERTRATE***
0.592810 -134.306174 0.781534 0.033482 3.269749 0.690837  +ROQI_IMPACT***  -WATER_RT***
-FAMSUPP_RT**  +WRAAGE_RT***  -FERTRATE***  +HHH_AVAIL**
******************************************************************************
Choose 7 of 18 Summary
                                                 Highest Adjusted R-Squared Results

AdjR2    AICc   JB K(BP)  VIF   SA   Model

 0.71 -143.42 0.43  0.45 3.98 0.79  +WALK_MRD**  +ROQI_IMPACT***  -LATRINE_RT***  +MARRIED_RT
-FAMSUPP_RT**  +WRAAGE_RT***  -FERTRATE***
 0.70 -143.13 0.47  0.47 3.93 0.45  +WALK_MRD*  +ROQI_IMPACT***  -LATRINE_RT***  -FAMSUPP_RT**
+WRAAGE_RT***  -FERTRATE***  +HHH_AVAIL
 0.70 -142.49 0.35  0.68 4.31 0.64  +WALK_MRD**  +ROQI_IMPACT**  -LATRINE_RT***  -PRVTRANS_RT
-FAMSUPP_RT***  +WRAAGE_RT***  -FERTRATE***
                                                                           Passing Models

AdjR2    AICc        JB       K(BP)    VIF      SA         Model

0.645867 -136.803684 0.761213 0.081722 3.329961 0.135850  +WALK_MRD***  +ROQI_IMPACT**
-PRVTRANS_RT***  +MARRIED_RT**  -FAMSUPP_RT**  +WRAAGE_RT***  -FERTRATE***
******************************************************************************
Choose 8 of 18 Summary
                                                       Highest Adjusted R-Squared Results

AdjR2    AICc   JB K(BP)  VIF   SA   Model

 0.71 -141.63 0.41  0.29 4.38 0.96  +WALK_MRD**  +ROQI_IMPACT**  -LATRINE_RT**  -PRVTRANS_RT
+MARRIED_RT  -FAMSUPP_RT***  +WRAAGE_RT***  -FERTRATE***
 0.71 -141.02 0.43  0.39 4.04 0.43  +WALK_MRD**  +ROQI_IMPACT***  -LATRINE_RT***  +MARRIED_RT
-FAMSUPP_RT**  +WRAAGE_RT***  -FERTRATE***  +HHH_AVAIL
 0.70 -140.48 0.54  0.63 4.31 0.66  +WALK_MRD**  +ROQI_IMPACT**  -LATRINE_RT***  -PRVTRANS_RT
-FAMSUPP_RT**  +WRAAGE_RT***  -FERTRATE***  +HHH_AVAIL
       Passing Models
AdjR2 AICc JB K(BP) VIF SA   Model
******************************************************************************
Choose 9 of 18 Summary
                                                             Highest Adjusted R-Squared Results

AdjR2    AICc   JB K(BP)  VIF   SA   Model

 0.71 -138.32 0.47  0.33 4.39 0.63  +WALK_MRD**  +ROQI_IMPACT**  -LATRINE_RT***  -PRVTRANS_RT
+MARRIED_RT  -FAMSUPP_RT**  +WRAAGE_RT***  -FERTRATE***  +HHH_AVAIL
 0.71 -138.12 0.43  0.35 4.38 0.68  +WALK_MRD**  +ROQI_IMPACT**  -LATRINE_RT***  -PRVTRANS_RT
```

Page 3

For peer review only

Exploratory Regression.txt

```
+MARRIED_RT  -FAMSUPP_RT**  +WRAAGE_RT***  -FERTRATE***  +WRAEDUC
 0.71 -138.07 0.43  0.56 4.41 0.95  +WALK_MRD**  +PUBDRVDRV  +ROQI_IMPACT**  -LATRINE_RT**
-PRVTRANS_RT  +MARRIED_RT  -FAMSUPP_RT***  +WRAAGE_RT***  -FERTRATE***
       Passing Models
AdjR2 AICc JB K(BP) VIF SA   Model
******************************************************************************
Choose 10 of 18 Summary
                                                                   Highest Adjusted R-Squared
Results
AdjR2    AICc   JB K(BP)  VIF   SA   Model

 0.71 -135.47 0.51  0.49 6.61 0.80  +WALK_MRD***  +ROQI_IMPACT**  -LATRINE_RT**  -PRVTRANS_RT*
+MARRIED_RT*  -FAMSUPP_RT**  +WRAAGE_RT***  -FERTRATE***  -HHHEDUC  +WRAEDUC
 0.71 -134.98 0.43  0.43 4.39 0.80  +WALK_MRD**  +ROQI_IMPACT**  -LATRINE_RT***  -PRVTRANS_RT
+MARRIED_RT  -FAMSUPP_RT**  +WRAAGE_RT***  -FERTRATE***  +HHH_AVAIL  +WRAEDUC
 0.71 -134.76 0.44  0.59 4.67 0.74  +WALK_MRD**  +PUBDRVDRV  +ROQI_IMPACT**  -LATRINE_RT**
-PRVTRANS_RT  +MARRIED_RT  -FAMSUPP_RT***  +FINAUTPRE_RT  +WRAAGE_RT***  -FERTRATE***
       Passing Models
AdjR2 AICc JB K(BP) VIF SA   Model
******************************************************************************
************** Exploratory Regression Global Summary (NEW_OUTC) **************

              Percentage of Search Criteria Passed
                   Search Criterion Cutoff Trials # Passed % Passed
             Min Adjusted R-Squared > 0.50 199139    25678    12.89
            Max Coefficient p-value < 0.05 199139       61     0.03
                      Max VIF Value < 4.50 199139    99568    50.00
            Min Jarque-Bera p-value > 0.10 199139   191512    96.17
Min Spatial Autocorrelation p-value > 0.10     51       43    84.31

------------------------------------------------------------------------------

        Summary of Variable Significance
Variable        % Significant % Negative % Positive
FERTRATE                99.82     100.00       0.00
FAMSUPP_RT              44.71      99.88       0.12
WRAAGE_RT               43.32      41.63      58.37
UNEMP_RT                27.99      92.68       7.32
ROQI_IMPACT             23.78       0.00     100.00
PRVTRANS_RT             19.97      94.84       5.16
LATRINE_RT              14.39      67.86      32.14
WALK_MRD                10.09       5.22      94.78
HHH_AVAIL                3.28       2.27      97.73
WATER_RT                 2.87      74.80      25.20
MARRIED_RT               2.77      19.79      80.21
PUBDRV                   1.74      29.98      70.02
PUB                      1.39      81.69      18.31
WRAEDUC                  0.86      30.60      69.40
PUBDRVDRV                0.79      60.09      39.91
HHHEDUC                  0.24      34.71      65.29
FINAUTPRE_RT             0.10      92.44       7.56
COMSUPP_RT               0.01      45.72      54.28

------------------------------------------------------------------------------


 Summary of Multicollinearity

Variable          VIF Violations Covariates
```


For peer review only

Exploratory Regression.txt

```
PUB            8.80   34028    PUBDRV (77.21), PUBDRVDRV (31.02), HHHEDUC (12.71), WRAEDUC (9.33),
LATRINE_RT (6.14), FERTRATE (4.87), WALK_MRD (3.30), WATER_RT (3.03), FAMSUPP_RT (0.12), WRAAGE_RT
(0.03), PRVTRANS_RT (0.02)
WALK_MRD       5.89    1610    PUB (3.30), PUBDRVDRV (2.98), PUBDRV (2.34), WATER_RT (0.44),
HHHEDUC (0.21), WRAEDUC (0.21), LATRINE_RT (0.11), FERTRATE (0.05), PRVTRANS_RT (0.02), FAMSUPP_RT
(0.01)
PUBDRV        14.41   63274    PUBDRVDRV (90.54), PUB (77.21), HHHEDUC (22.77), WRAEDUC (16.51),
LATRINE_RT (12.61), FERTRATE (6.57), WATER_RT (6.36), WALK_MRD (2.34), FAMSUPP_RT (0.12), WRAAGE_RT
(0.03), PRVTRANS_RT (0.01)
PUBDRVDRV      8.68   37253    PUBDRV (90.54), PUB (31.02), HHHEDUC (14.17), WRAEDUC (10.57),
LATRINE_RT (7.69), WATER_RT (5.32), FERTRATE (3.03), WALK_MRD (2.98), FAMSUPP_RT (0.02), PRVTRANS_RT
(0.01), WRAAGE_RT (0.01)
ROQI_IMPACT    1.94       0    --------


WATER_RT       5.29    6578    LATRINE_RT (7.08), PUBDRV (6.36), PUBDRVDRV (5.32), PUB (3.03),
HHHEDUC (2.00), WRAEDUC (1.66), FERTRATE (1.13), WALK_MRD (0.44), FAMSUPP_RT (0.00), WRAAGE_RT (0.00)

LATRINE_RT     6.10   13218    PUBDRV (12.61), PUBDRVDRV (7.69), WATER_RT (7.08), HHHEDUC (7.08),
WRAEDUC (6.79), PUB (6.14), FERTRATE (5.99), WALK_MRD (0.11), WRAAGE_RT (0.02)

PRVTRANS_RT    5.04       8    PUB (0.02), WALK_MRD (0.02), PUBDRVDRV (0.01), PUBDRV (0.01),
FAMSUPP_RT (0.00)

MARRIED_RT     1.68       0    --------


UNEMP_RT       3.69       0    --------


FAMSUPP_RT     5.19      54    PUBDRV (0.12), PUB (0.12), HHHEDUC (0.04), WRAEDUC (0.04), PUBDRVDRV
(0.02), WALK_MRD (0.01), WATER_RT (0.00), PRVTRANS_RT (0.00)

COMSUPP_RT     1.86       0    --------


FINAUTPRE_RT   2.26       0    --------


WRAAGE_RT      4.75      38    HHHEDUC (0.10), WRAEDUC (0.10), FERTRATE (0.09), PUB (0.03), PUBDRV
(0.03), LATRINE_RT (0.02), PUBDRVDRV (0.01), WATER_RT (0.00)

FERTRATE       5.42    8320    PUBDRV (6.57), LATRINE_RT (5.99), PUB (4.87), WRAEDUC (3.78),
HHHEDUC (3.69), PUBDRVDRV (3.03), WATER_RT (1.13), WRAAGE_RT (0.09), WALK_MRD (0.05)

HHH_AVAIL      2.62       0    --------


HHHEDUC        8.78   28637    WRAEDUC (58.81), PUBDRV (22.77), PUBDRVDRV (14.17), PUB (12.71),
LATRINE_RT (7.08), FERTRATE (3.69), WATER_RT (2.00), WALK_MRD (0.21), WRAAGE_RT (0.10), FAMSUPP_RT
(0.04)
WRAEDUC        8.89   24552    HHHEDUC (58.81), PUBDRV (16.51), PUBDRVDRV (10.57), PUB (9.33),
LATRINE_RT (6.79), FERTRATE (3.78), WATER_RT (1.66), WALK_MRD (0.21), WRAAGE_RT (0.10), FAMSUPP_RT
(0.04)

------------------------------------------------------------------------------
```

Page 5

For peer review only

```
                              Exploratory Regression.txt
                                                          Summary of Residual Normality
(JB)
      JB    AdjR2       AICc    K(BP)      VIF       SA   Model

0.999981 0.011731  -92.024434 0.002361 4.176647 0.053210  -PUB  +WALK_MRD  -PUBDRVDRV  +ROQI_IMPACT
-WATER_RT  +MARRIED_RT  -FAMSUPP_RT  +COMSUPP_RT  -WRAAGE_RT  +HHHEDUC
0.999979 0.520437 -123.545871 0.883158 4.638126 0.884202  +PUBDRV  -LATRINE_RT*  -UNEMP_RT*
-FINAUTPRE_RT  +WRAAGE_RT***  -FERTRATE***  -HHHEDUC  +WRAEDUC
0.999975 0.099522 -106.528531 0.002699 2.039475 0.005442  -PUBDRVDRV  +ROQI_IMPACT*  -WATER_RT
-FAMSUPP_RT  -WRAAGE_RT  +HHHEDUC

------------------------------------------------------------------------------

                                                           Summary of Residual Spatial
Autocorrelation (SA)
      SA    AdjR2       AICc       JB    K(BP)      VIF   Model

0.976892 0.638286 -140.613195 0.625085 0.345632 3.908376  +WALK_MRD***  +ROQI_IMPACT***
-LATRINE_RT***  +WRAAGE_RT***  -FERTRATE***
0.958004 0.713906 -141.625323 0.409253 0.288293 4.380150  +WALK_MRD**  +ROQI_IMPACT**  -LATRINE_RT**
-PRVTRANS_RT  +MARRIED_RT  -FAMSUPP_RT***  +WRAAGE_RT***  -FERTRATE***
0.954297 0.708834 -138.071313 0.431612 0.559128 4.414522  +WALK_MRD**  +PUBDRVDRV  +ROQI_IMPACT**
-LATRINE_RT**  -PRVTRANS_RT  +MARRIED_RT -FAMSUPP_RT***  +WRAAGE_RT***  -FERTRATE***

------------------------------------------------------------------------------

Table Abbreviations
AdjR2 Adjusted R-Squared
AICc  Akaike's Information Criterion
JB    Jarque-Bera p-value
K(BP) Koenker (BP) Statistic p-value
VIF   Max Variance Inflation Factor
SA    Global Moran's I p-value
Model Variable sign (+/-)
Model Variable significance (* = 0.10, ** = 0.05, *** = 0.01)

------------------------------------------------------------------------------
```


For peer review only - http://bmjopen.bmj.com/site/about/guidelines.xhtml

Summary of OLS Results - Model Variables

Variable	Coefficient [a]	StdError	t-Statistic	Probability [b]	Robust_SE	Robust_t	Robust_Pr [b]	VIF [c]
Intercept	-0.194483	0.198020	-0.982142	0.334442	0.198192	-0.981289	0.334855	--------
WALK_MRD	0.033353	0.014641	2.278046	0.030557*	0.015198	2.194592	0.036652*	2.065976
ROQI_IMPACT	0.023936	0.007537	3.175867	0.003620*	0.006860	3.489274	0.001620*	1.353110
LATRINE_RT	-0.003094	0.000722	-4.284566	0.000194*	0.000677	-4.572118	0.000088*	1.923468
FAMSUPP_RT	-0.001274	0.000514	-2.480549	0.019399*	0.000602	-2.115857	0.043382*	1.526477
WRAAGE_RT	0.034653	0.008260	4.195438	0.000247*	0.008113	4.271314	0.000201*	2.933353
FERTRATE	-0.222607	0.029423	-7.565753	0.000000*	0.030839	-7.218404	0.000000*	3.908531

OLS Diagnostics

Input Features:	LOCALITY_COMP_PREG	Dependent Variable:	NEW_OUTC
Number of Observations:	35	Akaike's Information Criterion (AICc) [d]:	-144.175618
Multiple R-Squared [d]:	0.747063	Adjusted R-Squared [d]:	0.692863
Joint F-Statistic [e]:	13.783283	Prob(>F), (6,28) degrees of freedom:	0.000000*
Joint Wald Statistic [e]:	139.475044	Prob(>chi-squared), (6) degrees of freedom:	0.000000*
Koenker (BP) Statistic [f]:	4.956762	Prob(>chi-squared), (6) degrees of freedom:	0.549371
Jarque-Bera Statistic [g]:	1.939051	Prob(>chi-squared), (2) degrees of freedom:	0.379263

Notes on Interpretation

* An asterisk next to a number indicates a statistically significant p-value (p < 0.01).

[a] Coefficient: Represents the strength and type of relationship between each explanatory variable and the dependent variable.

[b] Probability and Robust Probability (Robust_Pr): Asterisk (*) indicates a coefficient is statistically significant (p < 0.01); if the Koenker (BP) Statistic [f] is statistically significant, use the Robust Probability column (Robust_Pr) to determine coefficient significance.

[c] Variance Inflation Factor (VIF): Large Variance Inflation Factor (VIF) values (> 7.5) indicate redundancy among explanatory variables.

[d] R-Squared and Akaike's Information Criterion (AICc): Measures of model fit/performance.

[e] Joint F and Wald Statistics: Asterisk (*) indicates overall model significance (p < 0.01); if the Koenker (BP) Statistic [f] is statistically significant, use the Wald Statistic to determine overall model significance.

[f] Koenker (BP) Statistic: When this test is statistically significant (p < 0.01), the relationships modeled are not consistent (either due to non-stationarity or heteroskedasticity). You should rely on the Robust Probabilities (Robust_Pr) to determine coefficient significance and on the Wald Statistic to determine overall model significance.

[g] Jarque-Bera Statistic: When this test is statistically significant (p < 0.01) model predictions are biased (the residuals are not normally distributed).

For peer review only - http://bmjopen.bmj.com/site/about/guidelines.xhtml

Variable Distributions and Relationships

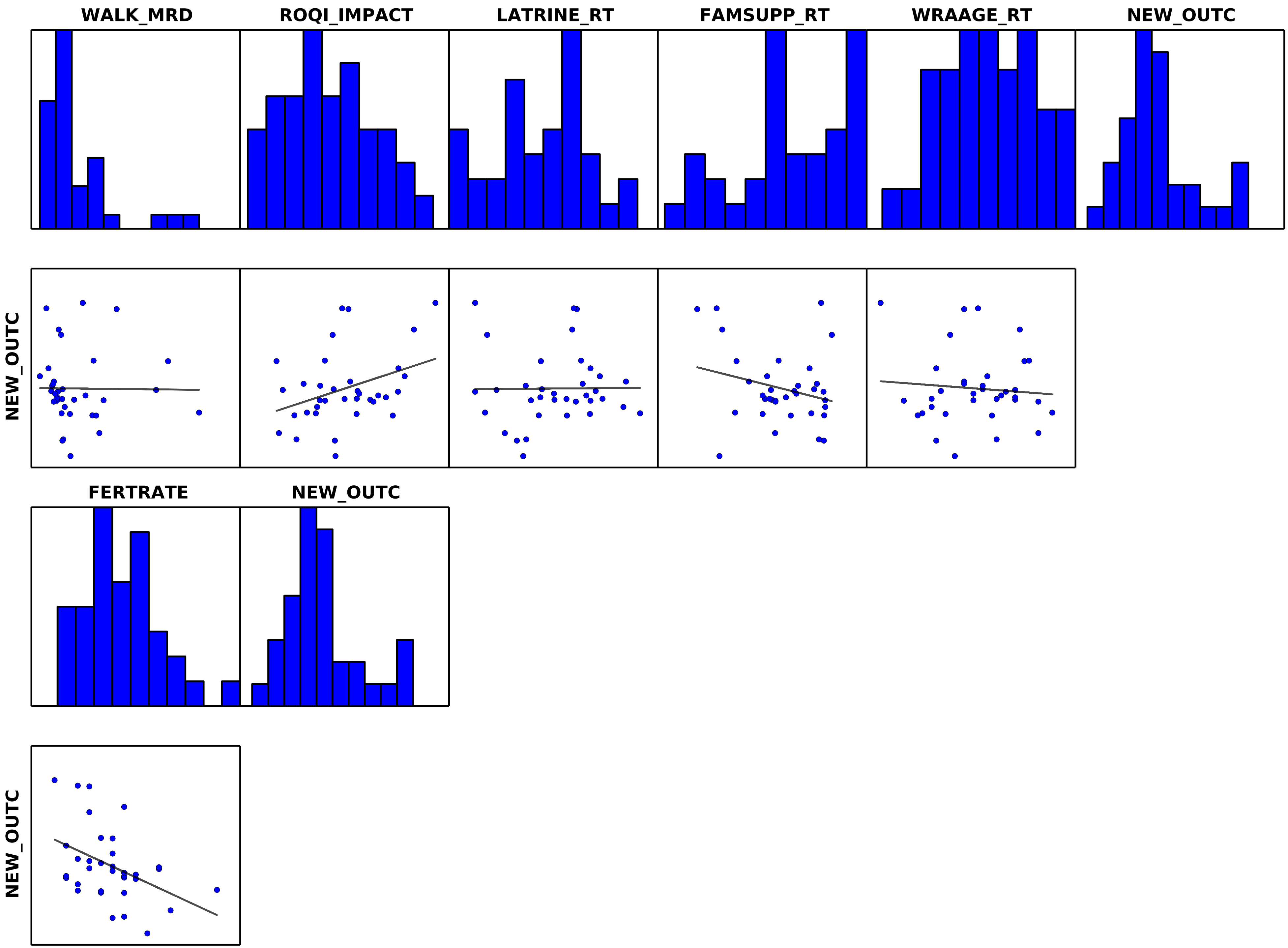

Variable Distributions and Relationships (Cont.)

The above graphs are Histograms and Scatterplots for each explanatory variable and the dependent variable. The histograms show how each variable is distributed. OLS does not require variables to be normally distributed. However, if you are having trouble finding a properly-specified model, you can try transforming strongly skewed variables to see if you get a better result.

Each scatterplot depicts the relationship between an explanatory variable and the dependent variable. Strong relationships appear as diagonals and the direction of the slant indicates if the relationship is positive or negative. Try transforming your variables if you detect any non-linear relationships. For more information see the Regression Analysis Basics documentation.

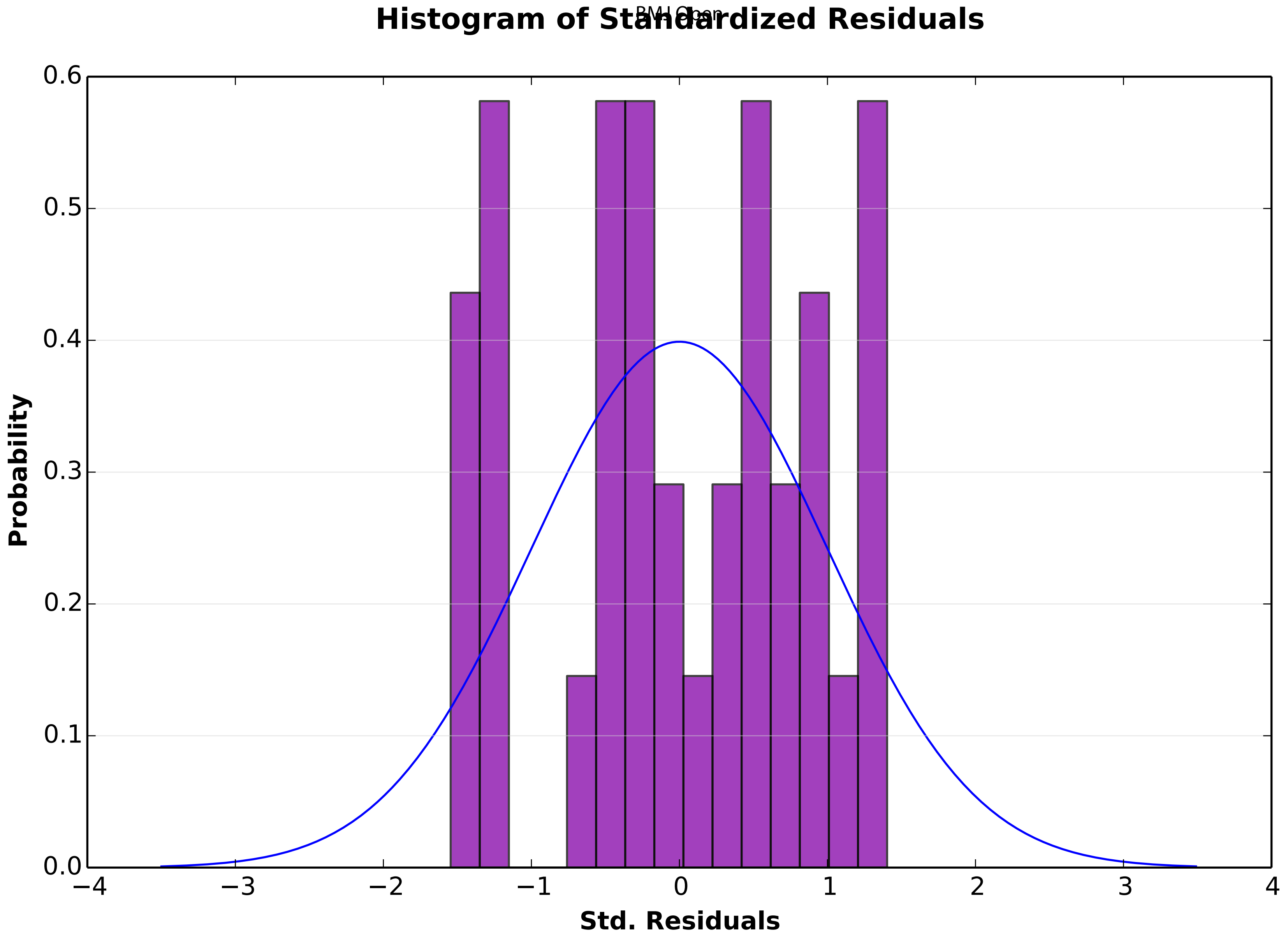

Ideally the histogram of your residuals would match the normal curve, indicated above in blue. If the histogram looks very different from the normal curve, you may have a biased model. If this bias is significant it will also be represented by a statistically significant Jarque-Bera p-value (*).

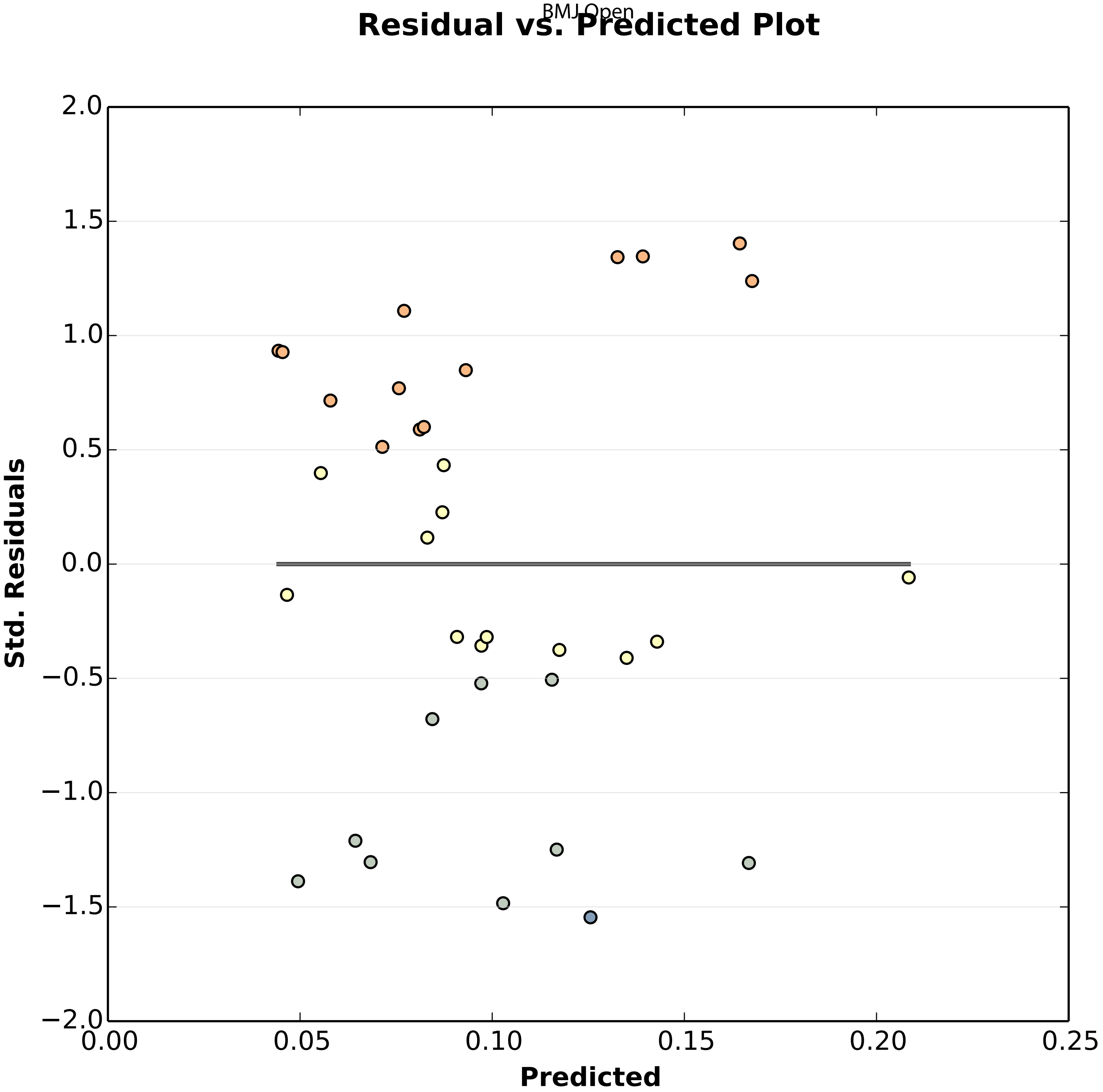

This is a graph of residuals (model over and under predictions) in relation to predicted dependent variable values. For a properly specified model, this scatterplot will have little structure, and look random (see graph on the right). If there is a structure to this plot, the type of structure may be a valuable clue to help you figure out what's going on.

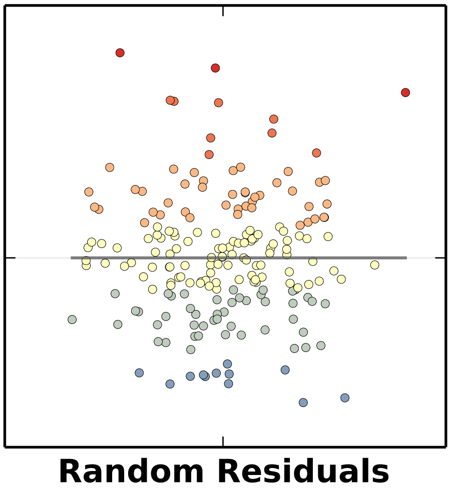

Ordinary Least Squares Parameters

Parameter Name	Input Value
Input Features	LOCALITY_COMP_PREG
Unique ID Field	UNI_ID
Output Feature Class	None
Dependent Variable	NEW_OUTC
Explanatory Variables	WALK_MRD
	ROQI_IMPACT
	LATRINE_RT
	FAMSUPP_RT
	WRAAGE_RT
	FERTRATE
Selection Set	False

```
                         Geographically Weighted Regression.txt
*****************************************************************************
*            Semiparametric Geographically Weighted Regression              *
*                       Release 1.0.90 (GWR 4.0.90)                         *
*                             12 May 2015                                   *
*               (Originally coded by T. Nakaya: 1 Nov 2009)                 *
*                                                                           *
*              Tomoki Nakaya(1), Martin Charlton(2), Chris Brunsdon (2)     *
*              Paul Lewis (2), Jing Yao (3), A Stewart Fotheringham (4)     *
*                       (c) GWR4 development team                           *
* (1) Ritsumeikan University, (2) National University of Ireland, Maynooth, *
*         (3) University of Glasgow, (4) Arizona State University           *
*****************************************************************************

Program began at 2018-04-07 9:13:44 AM

*****************************************************************************
Session: MOZ_GWR_2018
Session control file: C:\Users\Prestige\OneDrive\CANADA\UBC\PRE-EMPT\MOM\Papers\Spatial EPI of
Maternal and Neonatal deaths\DATA\GWR_moz_2018.ctl
*****************************************************************************
Data filename: C:\Users\Prestige\OneDrive\CANADA\UBC\PRE-EMPT\MOM\Papers\Spatial EPI of Maternal and
Neonatal deaths\ForGWR.csv
Number of areas/points: 35

Model settings---------------------------------
Model type: Gaussian
Geographic kernel: adaptive Gaussian
Method for optimal bandwidth search: fixed value
Criterion for optimal bandwidth: AICc
Number of varying coefficients: 7
Number of fixed coefficients:   0

Modelling options---------------------------------
Standardisation of independent variables: On
Testing geographical variability of local coefficients: On
Local to Global Variable selection: OFF
Global to Local Variable selection: OFF
Prediction at non-regression points: On

Variable settings---------------------------------
Area key: field1: OBJECTID
Easting (x-coord): field14 : POINT_X
Northing (y-coord): field15: POINT_Y
Cartesian coordinates: Euclidean distance
Dependent variable: field3: NEW_OUTC
Offset variable is not specified
Intercept: varying (Local) intercept
Independent variable with varying (Local) coefficient: field4: WALK_MRD
Independent variable with varying (Local) coefficient: field5: RoQI_IMPACT
Independent variable with varying (Local) coefficient: field6: LATRINE_RT
Independent variable with varying (Local) coefficient: field7: FAMSUPP_RT
Independent variable with varying (Local) coefficient: field8: WRAAGE_RT
Independent variable with varying (Local) coefficient: field9: FERTRATE
Number of prediction at non-regression points   98
*****************************************************************************

*****************************************************************************
  Global regression result
*****************************************************************************
```

Page 1

For peer review only

```
                                Geographically Weighted Regression.txt
  < Diagnostic information >
Residual sum of squares:                    0.018003
Number of parameters:                        7
 (Note: this num does not include an error variance term for a Gaussian model)
ML based global sigma estimate:             0.022680
Unbiased global sigma estimate:             0.025357
-2 log-likelihood:                       -165.714080
Classic AIC:                             -149.714080
AICc:                                    -144.175618
BIC/MDL:                                 -137.271295
CV:                                         0.000937
R square:                                   0.747063
Adjusted R square:                          0.681487

Variable                   Estimate    Standard Error      t(Est/SE)
-------------------- --------------- --------------- ---------------
Intercept                  0.100460        0.004298       23.374311
WALK_MRD                   0.014010        0.006150        2.278046
RoQI_IMPACT                0.015719        0.004950        3.175867
LATRINE_RT                -0.024529        0.005725       -4.284566
FAMSUPP_RT                -0.012849        0.005180       -2.480549
WRAAGE_RT                  0.030268        0.007214        4.195438
FERTRATE                  -0.063174        0.008350       -7.565753

*****************************************************************************
  GWR (Geographically weighted regression) bandwidth selection
*****************************************************************************

Bandwidth search <fixed value>:  30

*****************************************************************************
  GWR (Geographically weighted regression) result
*****************************************************************************
  Bandwidth and geographic ranges
Bandwidth size:                   30.000000
Coordinate                 Min              Max            Range
--------------- --------------- --------------- ---------------
X-coord          3616601.398000  3768895.758000   152294.360000
Y-coord         -2920426.694000 -2791357.504000   129069.190000

  Diagnostic information
Residual sum of squares:             0.015258
Effective number of parameters (model: trace(S)):                     8.181591
Effective number of parameters (variance: trace(S'S)):                7.198744
Degree of freedom (model: n - trace(S)):                             26.818409
Degree of freedom (residual: n - 2trace(S) + trace(S'S)):            25.835562
ML based sigma estimate:             0.020880
Unbiased sigma estimate:             0.024302
-2 log-likelihood:                -171.503374
Classic AIC:                      -153.140192
AICc:                             -145.606817
BIC/MDL:                          -138.859623
CV:                                  0.000880
R square:                            0.785624
Adjusted R square:                   0.706519

***********************************************************
 << Geographically varying (Local) coefficients >>
***********************************************************
                                                     Page 2
```

For peer review only

Geographically Weighted Regression.txt

Estimates of varying coefficients have been saved in the following file.
Listwise output file: C:\Users\Prestige\OneDrive\CANADA\UBC\PRE-EMPT\MOM\Papers\Spatial EPI of Maternal and Neonatal deaths\DATA\GWR_moz_2018_listwise.csv

Summary statistics for varying (Local) coefficients

Variable	Mean	STD
Intercept	0.101035	0.000664
WALK_MRD	0.012786	0.001721
RoQI_IMPACT	0.015974	0.000361
LATRINE_RT	-0.024667	0.000923
FAMSUPP_RT	-0.014028	0.001883
WRAAGE_RT	0.029602	0.001537
FERTRATE	-0.062852	0.001075

Variable	Min	Max	Range
Intercept	0.099979	0.101892	0.001913
WALK_MRD	0.010322	0.015769	0.005447
RoQI_IMPACT	0.015421	0.016749	0.001328
LATRINE_RT	-0.025996	-0.023045	0.002951
FAMSUPP_RT	-0.016372	-0.011305	0.005068
WRAAGE_RT	0.027676	0.031744	0.004068
FERTRATE	-0.064533	-0.061240	0.003293

Variable	Lwr Quartile	Median	Upr Quartile
Intercept	0.100351	0.101371	0.101613
WALK_MRD	0.011107	0.012520	0.014249
RoQI_IMPACT	0.015689	0.015875	0.016267
LATRINE_RT	-0.025567	-0.024590	-0.024033
FAMSUPP_RT	-0.015926	-0.013778	-0.012218
WRAAGE_RT	0.028076	0.029603	0.031222
FERTRATE	-0.063924	-0.062449	-0.061996

Variable	Interquartile R	Robust STD
Intercept	0.001262	0.000936
WALK_MRD	0.003142	0.002329
RoQI_IMPACT	0.000579	0.000429
LATRINE_RT	0.001534	0.001137
FAMSUPP_RT	0.003708	0.002748
WRAAGE_RT	0.003146	0.002332
FERTRATE	0.001927	0.001429

(Note: Robust STD is given by (interquartile range / 1.349))

****

GWR ANOVA Table

****

Source	SS	DF	MS	F
Global Residuals	0.018	28.000		
GWR Improvement	0.003	2.164	0.001	
GWR Residuals	0.015	25.836	0.001	2.147056

****

Geographical variability tests of local coefficients

****

Variable	F	DOF for F test	DIFF of Criterion

Page 3

For peer review only

Geographically Weighted Regression.txt

Intercept	2.000828	0.142	26.818	0.176197
WALK_MRD	7.975741	0.195	26.818	-1.224025
RoQI_IMPACT	0.748443	0.185	26.818	0.530483
LATRINE_RT	3.943637	0.125	26.818	-0.156581
FAMSUPP_RT	15.587840	0.164	26.818	-2.551618
WRAAGE_RT	2.703057	0.215	26.818	0.073263
FERTRATE	2.022504	0.157	26.818	0.190761

Note: positive value of diff-Criterion (AICc, AIC, BIC/MDL or CV) suggests no spatial variability in terms of model selection criteria.
F test: in case of no spatial variability, the F statistics follows the F distribution of DOF for F test.

****
Program terminated at 2018-04-07 9:13:45 AM

For peer review only
